# Synthesis of Novel Thiazole Derivatives Bearing β-Amino Acid and Aromatic Moieties as Promising Scaffolds for the Development of New Antibacterial and Antifungal Candidates Targeting Multidrug-Resistant Pathogens

**DOI:** 10.3390/molecules27010074

**Published:** 2021-12-23

**Authors:** Dovilė Malūkaitė, Birutė Grybaitė, Rita Vaickelionienė, Giedrius Vaickelionis, Birutė Sapijanskaitė-Banevič, Povilas Kavaliauskas, Vytautas Mickevičius

**Affiliations:** 1 Department of Organic Chemistry, Kaunas University of Technology, Radvilėnų Rd. 19, LT-50254 Kaunas, Lithuania; dovile.malukaite@ktu.edu (D.M.); birute.grybaite@ktu.lt (B.G.); giedrius.vaickelionis@ktu.lt (G.V.); birute.sapijanskaite@ktu.lt (B.S.-B.); pok4001@med.cornell.edu (P.K.); vytautas.mickevicius@ktu.lt (V.M.); 2 Weill Cornell Medicine of Cornell University, 527 East 68th Street, New York, NY 10065, USA; 3 Institute for Genome Sciences, School of Medicine, University of Maryland, 655 W. Baltimore Street, Baltimore, MD 21201, USA; 4 Biological Research Center, Veterinary Academy, Lithuanian University of Health Sciences, Tilžės Str. 18, LT-47181 Kaunas, Lithuania; 5 Institute of Infectious Diseases and Pathogenic Microbiology, Birštono Str. 38A, LT-59116 Prienai, Lithuania

**Keywords:** azole, thiazoles, β-amino acids, antimicrobial activity

## Abstract

Rapidly growing antimicrobial resistance among clinically important bacterial and fungal pathogens accounts for high morbidity and mortality worldwide. Therefore, it is critical to look for new small molecules targeting multidrug-resistant pathogens. Herein, in this paper we report a synthesis, ADME properties, and in vitro antimicrobial activity characterization of novel thiazole derivatives bearing β-amino acid, azole, and aromatic moieties. The in silico ADME characterization revealed that compounds **1**–**9** meet at least 2 Lipinski drug-like properties while cytotoxicity studies demonstrated low cytotoxicity to Vero cells. Further in vitro antimicrobial activity characterization showed the selective and potent bactericidal activity of **2a**–**c** against Gram-positive pathogens (MIC 1–64 µg/mL) with profound activity against *S. aureus* (MIC 1–2 µg/mL) harboring genetically defined resistance mechanisms. Furthermore, the compounds **2a**–**c** exhibited antifungal activity against azole resistant *A. fumigatus,* while only **2b** and **5a** showed antifungal activity against multidrug resistant yeasts including *Candida auris*. Collectively, these results demonstrate that thiazole derivatives **2a**–**c** and **5a** could be further explored as a promising scaffold for future development of antifungal and antibacterial agents targeting highly resistant pathogenic microorganisms.

## 1. Introduction

Increasing antimicrobial resistance (AR) results in high morbidity and mortality worldwide [1]. The highest impact of AR is often more evident in developing countries, although AR remains a major global healthcare challenge. The World Health Organization in 2015 released the action plan aimed to fight the antimicrobial resistance among bacterial and fungal pathogens, although despite the efforts the resistance remains one of the leading threats [1,2]. Therefore, it is crucial to explore novel scaffolds leading to the development of future antimicrobial compounds.

Heterocycles being the widest division of organic chemistry occupy a unique place not only in nature but provide large-scale biologically active synthetic compounds with wide variety of properties and this number is permanently increasing. The majority of pharmaceuticals are from four-, up to eight-membered heterocycles or their fused derivatives with one or more the same or different heteroatoms in the structure. Straightforward synthesis and the large scale of biological properties of heterocyclic compounds is the best offer for the designing and development of the biologically active molecules [3].

The importance of heterocycles is well illustrated by their presence in many natural drugs such as atropine, quinine, theophylline, reserpine, papaverine, morphine, lodopyridone, and many others [4,5,6], and green pigment chlorophyll, hemoglobin, antibiotic penicillin are heterocyclic compounds.

One or several nitrogen heteroatoms containing cycles have been largely described to have many pharmacological actions such as anticancer [7], antiviral [8], anti-inflammatory [9], antidiabetic [10], antihypertensive [11], antitubercular [12], bronchodilator, antimicrobial [13], anticonvulsant [14], are also used in the treatment of Alzheimer’s disease [15] etc.

Oxygen-containing heterocycles as those of the nitrogen analogues are widely present in various kinds of natural products, such as carbohydrates, polyketides, peptides, and terpenoids, which show large potential of diverse pharmacological properties [16,17,18,19,20,21,22]. Nature-derived O-heterocycle pharmaceuticals salinomycin and Taxol are used to treat cancer, artemisinin is effective antimalarial agent, digoxin is a cardiac glycoside, and has inotropic effects in addition to effects on cardiac output, and codeine and morphine are opioid drugs [23].

Other heterocycles, especially S-heterocycles, are no less attractive than N-analogues. Those derivatives are known to possess anticancer, antidiabetic, anti-depressant, bronchodilator, anti-platelet, diuretic, anti-inflammatory, antiviral, anti-ulcer, and many other properties [24,25]. The high volatility and reactivity of sulfur determines that many sulfur-containing compounds also are used in flavoring of food products [26].

The importance of N-, O-, and S-containing heterocyclic compounds is obvious; however, the value of other heterocycles cannot be ruled out. Organoselenium and organophosphorus compounds indicate the power in the construction of molecules of medicinal interest and represent an important class of compounds with large potential for pharmaceutic applications [27,28].

Azoles have been long known as an important core in designing of various pharmaceutical agents. They can also be used as intermediates for the synthesis pharmaceuticals, for example, ambrisentan, a drug for the treatment of pulmonary hypertension [29]. The synthetic azole antimycotics constitute the largest group of antifungal agents currently widely used in clinical practice [30]. The antifungal activity of azoles against clinically important fungi is the fungal wall directed and mediated by the inhibition of ergosterol synthesis [31]. Due to strong and broad-spectrum antifungal activity, azole antifungal drugs are a first-line choice for the treatment and prevention of invasive fungal infections.

The overuse of azoles in the clinical field as well as the use of azole moieties containing herbicides in agriculture lead to the development of antifungal resistance among the clinical and environmental strains [32]. Therefore, novel compounds with selective antifungal activity and good tolerability are needed to overcome the rapidly growing problem.

Various antifungal azoles such as miconazole, clotrimazole, and ketoconazole demonstrate fungistatic activity. Azoles can inhibit fungal growth and virulence but not directly target the viability. Therefore, azoles alone are often used to treat superficial mycoses [30]. Newer generation of azole containing antifungals was developed and successfully demonstrated to be active against systemic fungal infections using both animal models and clinical trials [33]. Systemic triazoles such as fluconazole, itraconazole, isavuconazole, posaconazole, and voriconazole were developed and used to treat infections caused by *Candida, Aspergillus*, and other yeasts and mold [34]. Despite that, increasing resistance and resistance-associated treatment failures are now often observed [35,36,37].

Azole moiety-containing compounds also have promising anticonvulsant [38], antimicrobial [39,40,41,42], antiurease [43], anti-inflammatory [44], and antioxidant [45], as well as analgesic [46] properties. The combining of steroid with azole pharmacophore generated potential lead compounds with superior anticancer properties [47].

The various azole-based molecules, such as benzimidazole, imidazoles, pyrazole, triazole, thiazole, and others with a wide variety of functionalizations and coordination modes, can be an effective tool for the preparation of metal complexes [48]. Azoles in combination with metals provide a new alternative of efficient drugs, even against drug-resistant pathogens [49,50,51]. Although the complexes may demonstrate different anti-microbial activity [52,53,54], anticancer activity of such compounds is more predominant. Therefore, it is crucial to consider various pharmacological, excretion, metabolism, and toxicity properties while designing novel compounds [55].

Profound biological activity of azole derivatives makes them as attractive building blocks to develop novel antimicrobial candidates for the further pre-clinical development [55,56,57,58,59,60]. In this paper we describe the synthesis and in vitro antimicrobial activity characterization of novel thiazole derivatives bearing N-acyl hydrazone, pyrrole, pyrazole, and triazole.

## 2. Results

### 2.1. Synthesis

**Scheme 1:**
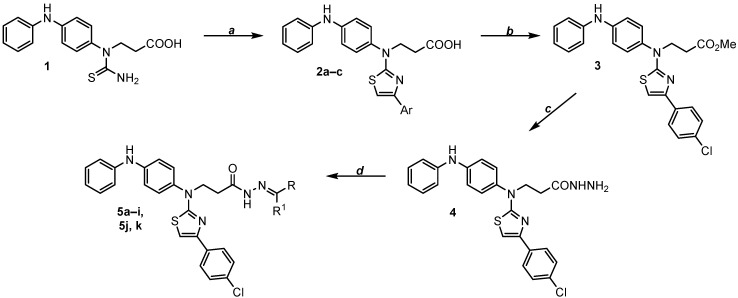
Synthesis of thiazoles **2**–**5**. **2a,** Ar = 4-FC_6_H_4_; **2b,** Ar = 4-ClC_6_H_4_; **2c,** Ar = 4-BrC_6_H_4_; **5a,** R = C_6_H_5_; **5b,** R = 4-FC_6_H_4_; **5c,** R = 4-ClC_6_H_4_; **5d,** R = 4-BrC_6_H_4_; **5e,** R = 4-O_2_NC_6_H_4_; **5f,** R = 4-Me_2_NC_6_H_4_; **5g,** R = 4-MeC_6_H_4_; **5h,** R = thien-2-yl; **5i,** R = 5-nitrothien-2-yl; **5a**–**i,** R^1^ = H; **5j,** R = R^1^ = Me; **5k,** R = Et, R^1^ = Me. Reagents and conditions: (**a**) 10% Na_2_CO_3_, MeOH, 2-bromo-4′-substituted acetophenone**,** Δ, 2 h (**a,b**), 4 h (**c**), 30% AcOH to pH 6, 68–88%; (**b**) MeOH, H_2_SO_4_, Δ, 5 h, 10% Na_2_CO_3_, 80%; (**c**) N_2_H_4_∙H_2_O, 2-PrOH, Δ, 18 h, H_2_O, 73%; (**d**) RCHO, AcOH, 2-PrOH, Δ, 2 h (**5a**–**i**), Me_2_CO (**5j**) or MEK (**5k**), Δ, 5 h, 68–95%.

In this study, the prepared compound **1** [61,62] was used as a starting precursor for the preparation of thiazoles **2a**–**c** by the Hantsch method by combining thioureido acid **1** with the corresponding acetophenones (Scheme 1). The structures of the obtained compounds **2** were confirmed by the data of the ^1^H and ^13^C NMR spectra (Supplementary material, Figures S3–S8). Using the reactivity of carboxylic group, the esterification of compound **2b** and subsequent hydrazinolysis of the resulting ester **3** was performed. In the NMR spectra for ester **3**, proton signals of the methoxy group are visible as an intense singlet at 3.54 ppm (^1^H), and signal of carbon of the same group resonate at 51.43 (^13^C) ppm. The spectra of the obtained hydrazide **4** showed the characteristic signals for the CONHNH_2_ fragment – two singlets at 9.10 and 4.17 ppm (^1^H), respectively, and the resonance line at 170.03 ppm for carbon of the C=O group (^13^C).

**Scheme 2:**
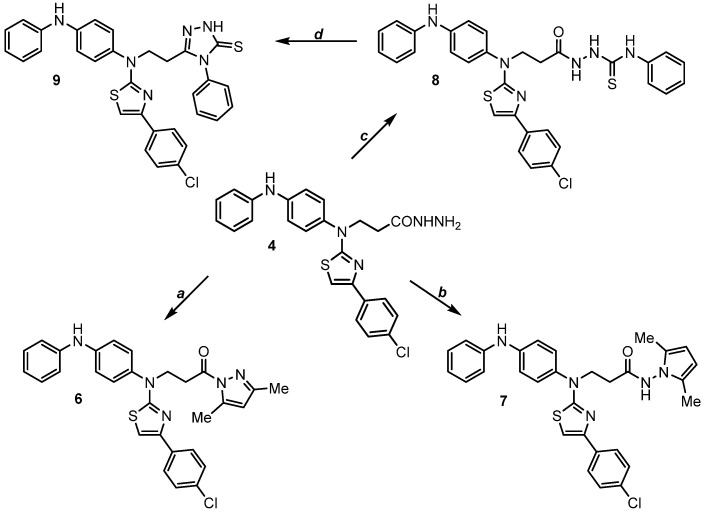
Synthesis of azoles **6**–**9**. Reagents and conditions: (**a**) 2-PrOH, 2,4-PD (dropwise), HCl, Δ, 9 h, 74%; (**b**) 2-PrOH, 2,5-HD (dropwise), AcOH, Δ, 3 h, 80%; (**c**) MeOH, PhNCS (dropwise), Δ, 2 h, H_2_O, 93%; (**d**) 4% NaOH, Δ, 3 h, AcOH to pH 6, 83%.

Then the obtained hydrazide **4** was used as a starting material for the synthesis of hydrazones **5** and azoles **6**, **7**, as well as **9**, which was prepared through the thiosemicarbazide intermediate **8** (Scheme 2). Condensation of compound **4** with aromatic aldehydes or carbaldehydes in propan-2-ol and using a catalytic amount of acetic acid led to the formation of N’-benzylidene hydrazides **5a**–**i**, while heating of hydrazide **4** at reflux with acetone or 2-butanone (MEK) yielded N′-(propan- or butan-2-ylidene)propanehydrazides **5j**, **k**. The stereochemistry of the synthesized hydrazones was ascribed by ^1^H NMR spectra. The NMR spectra of **5a**–**g** exhibited double sets of signals of the CONH group protons which is caused by a restricted rotation around the amide bond. The splitting of the proton signals indicates the formation of the Z/E isomers mixture in DMSO-*d_6_* solutions, where usually Z-form predominates [63]. The intense ratio of the signals of the isomers was found to be 65:35. For compounds **5h**, **i**, this ratio was observed 55:45 and 60:40, respectively. It is noteworthy that the substitution at the azomethine fragment in compounds **5j**, **k** resulted in the formation of a mixture of geometrical isomers, the presence of which is clearly reflected in the ^1^H NMR spectra of the compounds [64] (Supplementary Material, Figures S31–S34). Accordingly, hydrazones **5j**, **k** in DMSO-*d_6_* solutions exist as a mixture of geometrical isomers and amide conformers. The ^1^H NMR spectrum of derivative **5k** (Supplementary Material, Figure S33) showed the splitting of the characteristic signals of the ethyl and methyl protons in the intervals of 0.76–1.01, 2.11–2.24 ppm for CH_3_CH_2_, and singlet peaks at 1.79, 1.83, and 1.87 ppm for CH_3_ which were integrated for 3, 2, and 3 protons, respectively, as well as NH protons of the amide bond resonated as four singlets at 9.99, 10.07, 10.12, and 10.16 ppm which were totally integrated for 1 proton assigned for the amide (CONH) proton.

In the next step of the study, considering the immense biological properties of various azoles, we chose to obtain several five-membered heterocyclic derivatives and to evaluate their biological properties. Condensation of hydrazide **4** with the corresponding diketone—2,4-pentanedione (2,4-PD) and 2,5-hexanedione (2,5-HD) catalyzed by hydrochloric or acetic acid, respectively, gave 3,5-dimethylpyrazole derivative **6** and 2,5-dimethylpyrrole **7** (Scheme 2). The formation of the appropriate desired ring was approved by the NMR spectra of these compounds. Characteristic intense singlets at 2.16, 2.36, and 6.26 ppm (^1^H) and resonances at 15.79, 15.85, and 110.10 (^13^C) were assigned to the protons and carbons of the methyl groups and the CH fragment of the pyrazole cycle for compound **6**, respectively. The presence of a pyrrole ring was proven by the signals at 1.94, 10.91, and 11.02 ppm (^1^H, ^13^C, 2CH_3_) as well as 5.61 and 102.90 ppm (^1^H, ^13^C, CH-CH_pyr_) which are typical for such a structure.

Triazole derivative **9** was obtained in a two-step manner, by addition of phenyl isothiocyanate to a solution of hydrazide **4** in methanol to give carbothioamide **8**, which was then subjected to a cyclization step in basic conditions (4% NaOH aqueous solution) to afford 1,2,4-triazole-3-thione compound 9 in an 83% yield. In the ^13^C NMR spectrum of a linear structure, compound **8** carbon signal of the C=S fragment resonated at 180.82 ppm, while in the spectrum of a cyclic-form **9**, the resonance line of the C=S was observed upfield (167.90 ppm). Noteworthy, in the ^1^H NMR spectrum of triazole **9**, the singlet of NH group proton was characteristically shifted downfield (13.70 ppm) in comparison with the spectrum of non-cyclic compound **8**.

All the compounds were characterized by the ^1^H, ^13^C NMR, and IR spectroscopy and the data of elemental analysis. The NMR spectra are presented in the Supplementary material, Figures S1–S42.

### 2.2. Characterization of Absorption, Distribution, Metabolism, and Excretion (ADME) Properties

Generating poly-bioactive compounds with favorable bioavailability is paramount in medicinal chemistry. After successfully synthesizing and characterizing the compounds, we used in silico prediction tool based on Lipinski drug-likeness rule of 5 to characterize the ADME properties of the compounds **1**–**9 [65,66,67]**. The compounds were considered as drug-like when they met the following criteria: lipophilicity: XLOGP3 between −0.7 and +5.0, size: MW between 150 and 500 g/mol, polarity: TPSA between 20 and 130 Å^2^, solubility: log *S* not higher than 6, saturation: fraction of carbons in the sp^3^ hybridization not less than 0.25, and flexibility: no more than 9 rotatable bonds. We first evaluated the physicochemical properties on compounds **1**–**9** (Table 1). Based on physicochemical properties, most of the analyzed compounds met at least 2 rules of drug-likeness (Table 1).

Compounds **1**–**9** demonstrated good drug-like properties based on the numbers of hydrogen bond acceptors and donors (Table 1) while only compounds **1**–**2c** met molecular refractivity criteria.

After characterizing the physicochemical properties of the synthesized compounds, we further determined the pharmacokinetic, excretion, and metabolism profiles of compounds **1**–**9** (Table 2).

All compounds demonstrated good, predicted skin permeability suggesting the possibility to use the most potent compounds as possible topical antimicrobial agents. All compounds except thioureido acid were predicted to be non-absorbable in the gastrointestinal tract.

P glycoprotein 1-mediated drug transport is an important parameter often limiting cellular drug intake. Compounds **3** (ester)**, 5j** (aliphatic hydrazone), and **5k** (aliphatic hydrazone) were predicted to be a substrates of P glycoprotein 1. Finally, structure-dependent interactions with cytochrome metabolism system components were predicted for the novel thiazole derivatives bearing β-amino acid and aromatic moieties. Further in vitro and in vivo studies are needed to validate the in silico ADME results.

### 2.3. Antimicrobial Activity of Compounds **1**–**9**

After characterizing in silico ADME properties of the compounds **1**–**9** and demonstrating the good skin permeability of the compounds, we further hypothesized weather the compounds could be used as topical antimicrobials targeting skin colonizing multidrug-resistant microbial pathogens.

We used a panel of bacterial (*Staphylococcus aureus*, *Enterococcus spp*.*, Klebsiella pneumoniae*, *Stenotrophomonas maltophilia*, *Pseudomonas aeruginosa*) and fungal (*Candida auris*, *Candida albicans*, *Aspergillus fumigatus*) pathogens to evaluate the antimicrobial activity of the thiazole derivatives bearing β-amino acid and aromatic moieties.

First, we screened our compounds on Gram-positive and Gram-negative bacterial pathogens harboring genetically defined resistance mechanisms. Only compounds **2a**–**c** demonstrated an antimicrobial activity against Gram-positive bacterial pathogens. The highest antimicrobial activity was observed against *S. aureus* (MIC 1–64 µg/mL) while weak antibacterial activity (MIC 32–64 µg/mL) was observed when *Enterococcus* *spp*. were used for the assay (Table 3). Compounds **2a** (4F-C_6_H_4_) and **2b** (4Cl-C_6_H_4_) demonstrated the highest antibacterial activity against *S. aureus* with genetically defined resistance mechanisms (MIC 1–2 µg/mL), moreover compound **2a** was also efficient against multidrug-resistant *Enterococcus* spp. (MIC 32–64 µg/mL).

Notably, the antimicrobial activity of compounds **2a**, **b** against methicillin resistant *S. aureus* (MRSA) was similar to vancomycin (MIC 2 µg/mL). Strikingly, **2b** demonstrated profound activity against vancomycin resistant *S. aureus* (Table 3). Furthermore, none of the compounds exhibited antimicrobial activity against any Gram-negative bacterial pathogen that was used for the study, suggesting that the novel thiazole derivatives bearing 4-ClC_6_H_4_ and 4-FC_6_H_4_ substitutions are critical for the selective, Gram-positive bacteria directed antibacterial activity. Numerous thiazoles have been previously explored as antifungal agents [68,69,70]. Therefore, we further evaluated the antifungal activity of compounds **1**–**9** against azole resistant *A. fumigatus* harboring L98H, TR_34_ and F495I, L98H, S297T, and TR34 mutation in CYP51A protein conferring resistance to azole antifungals [71,72].

Compounds **2a**–**c** demonstrated the structure-dependent antifungal activity on azole-resistant *A. fumigatus* while low antifungal activity was observed on wild type (Wt), pan-susceptible *A. fumigatus* (Table 4). These results suggest that thiazole derivatives might be targeting upstream or downstream of mutated CYP51A pathway, due to the lack of susceptible phenotype in Wt *A. fumigatus* strain with pan-susceptible phenotype. Therefore **2a**–**c** scaffold could be potentially further optimized to generate the compounds targeting azole-resistant *A. fumigatus*.

We further decided to step forward and evaluate the in vitro antifungal activity of compounds **1**–**9** against extensively resistant strains of *Candida* including multidrug-resistant priority pathogen *Candida auris* (Table 5) [73,74].

When compounds **1**–**9** were screened against azole resistant *Candida auris*, *C. duobushaemulonii*, *C. krusei*, and *C. albicans*, only compounds **2b** and **5a** exhibited antifungal activity against multidrug-resistant yeasts.

Compound **2b** showed antifungal activity against *C. auris* and *C. duobushaemulonii* but not *C. krusei* or *C. albicans* suggesting that the 4-ClC_6_H_4_ substituent is critical for the antifungal activity against *C. auris*. Interestingly, the addition of phenyl substituent (compound **5a**) resulted in decreased antifungal activity against *C. auris* and *C. duobushaemulonii* in comparison to compound **2b** but restored activity against *C. krusei* (Table 5).

Collectively, these data demonstrate the wide spectrum antimicrobial activity of compounds **2a**–**c** and **5a.** The structure–activity relationship study (SAR) revealed that the 4-ClC_6_H_4_ and the 4-FC_6_H_4_ substituents are critical for the antibacterial and antifungal activity, while additional substitution in phenyl ring can affect the antifungal potency and species spectrum against clinically important and multidrug-resistant yeasts.

### 2.4. The Cytotoxicity Evaluation of Compounds **1**–**9**

**Figure 1:**
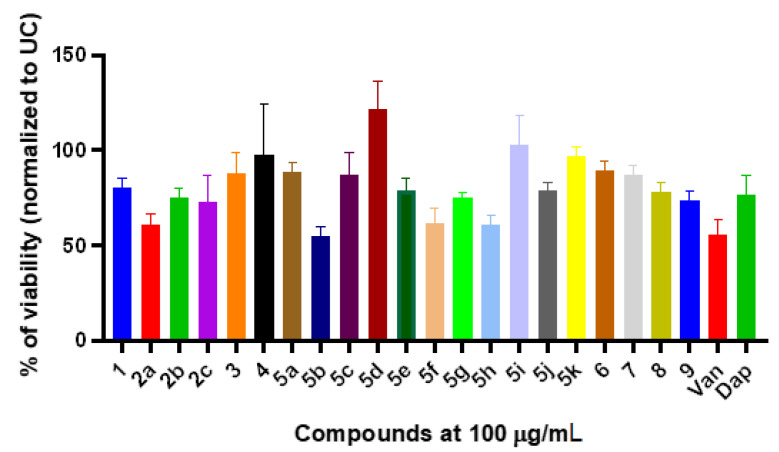
The cytotoxicity evaluation of compounds **1**–**9** in Vero African green monkey kidney cells. The Vero cells were exposed with fixed (100 µg/mL) concentration of each compound or vancomycin (Van) and daptomycin (Dap) that served as a comparator drug. The cells were incubated with test compounds for 48 h and the resulting viability was measured using MTT assay. Data provided in the graphs are mean ± SD from 3 experimental replicas.

After characterizing the in vitro antimicrobial activity of we further characterized the in vitro cytotoxic activity of compounds **1**–**9** and compared it to the cytotoxicity of two clinically approved drugs used for treatment of the infections caused by Gram-positive pathogens. We used non-cancerous Vero African green monkey kidney cells to measure the cytotoxicity of the compounds **1**–**9** as well as vancomycin (Van) and daptomycin (Dap) that served as a control. The compounds **1**–**9** demonstrated the structure-depended cytotoxic activity on Vero cells, although the compound induced toxicity was similar or lower than comparator drugs, that was comparable to the activity of vancomycin and daptomycin (Figure 1). Vancomycin (Van) exhibited the highest cytotoxic activity on Vero cells, by reducing the viability to 56% after 48-h exposure (Figure 1). Daptomycin demonstrated lower cytotoxicity to Vero cells with resulting 76.7% viability after 48-h exposure.

The compound **5b** bearing 4-fluorophenyl substituent demonstrated the highest cytotoxicity to Vero cells by decreasing the viability to 54.8%. Notably, the most promising compounds **2a**–**c** exhibiting good antimicrobial activity demonstrated similar or lower cytotoxicity in comparison to Van or Dap (Figure 1). The 48-h exposure with compound **2a** resulted in 61.2% viability while compounds **2b** and **2c** resulted in 75.1% and 72.3% viability respectively (Figure 1). These results demonstrate that the most promising compounds **2a**–**c** are exerting similar or lower cytotoxicity profiles than standard care drugs (Van or Dap).

### 2.5. The Compounds **2a**–**c** Demonstrates Bactericidal Activity on Staphylococcus Aureus with Genetically Defined Resistance Mechanisms

**Figure 2:**
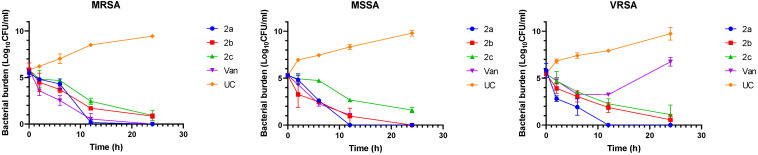
The in vitro time-kill studies of most potent compounds **2a**–**c** against *Staphylococcus aureus* harboring genetically defined resistance mechanisms. The representative *S. aureus* strains (MRSA, MSSA, VRSA) were exposed at MIC concentration of compounds **2a**–**c** or vancomycin for 24 h. The viable bacterial numbers in treatment groups or untreated control (UC) were calculated after dilution and plating. Data provided in the graphs are mean ± SD from 3 experimental replicas.

We further characterized the *S. aureus*-directed in vitro antimicrobial activity of the most promising compounds **2a**–**c** against representative strains with genetically defined resistance mechanisms. By using the time kill assay, we characterized the mode and kinetics of compounds-mediated *S. aureus* killing in vitro by exposing the bacteria at MIC concentration of the compounds or control drug (vancomycin; Van) (Figure 2). The time-kill assay demonstrated that compounds **2a**–**c** can rapidly kill the *S. aureus* in comparison to untreated control (UC) (*p <* 0.05). The compounds did not demonstrate the killing dependence on already pre-existing *S. aureus* resistance mechanisms. The most potent compound **2a** bearing the 4-fluorophenyl moiety demonstrated the highest bactericidal activity against *S. aureus* and was able to rapidly kill MSSA, MRSA, and VRSA in 6 h, suggesting the importance of 4-fluorophenyl substitution. The **2a** bactericidal activity against MSSA and MRSA was comparable to vancomycin (*p <* 0.05) (Figure 2). Moreover, the compound **2a** exhibited a significantly better bactericidal activity against VRSA in comparison to vancomycin (*p <* 0.05). Compound **2b** was able to completely kill the MSSA in 24 h but did not fully cleared MRSA and VRSA *S. aureus.* The compound **2c** demonstrated the time-depended antimicrobial activity against all *S. aureus* strains but failed to fully clear the viable bacteria. These data demonstrate that the in vitro antimicrobial activity of novel thiazole derivatives **2a, b** is mostly bactericidal and highly depended on the halophenyl substitution.

### 2.6. The Activity of the Compounds **2a**–**c** on the Staphylococcus Aureus Biofilm Integrity

**Figure 3:**
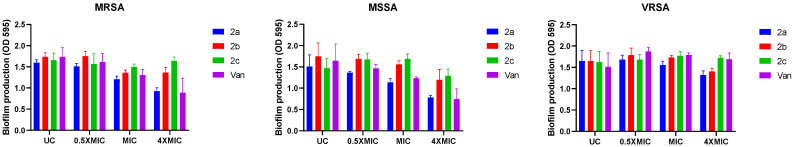
The activity of compounds **2a**–**c** on the *Staphylococcus aureus* biofilm integrity. The *S. aureus* biofilms were exposed with increasing concentrations of compounds **2a**–**c** or vancomycin (Van) for 24 h. After exposure with the test compounds, the biofilm integrity was evaluated by crystal violet assay. Data provided in the graphs are mean ± SD from 3 experimental replicas.

We further hypothesized that rapid killing effect of the most promising compounds **2a**–**c** could be further explored as a *S. aureus* biofilm disrupting strategy. We exposed the *S. aureus* biofilms with increasing concentrations of compounds **2a**–**c** or vancomycin (Van) for 24 h and evaluated the effect on *S. aureus* biofilm integrity by using crystal violet assay. The compound **2a, b** was able to disrupt the *S. aureus* biofilm integrity at the MIC or 4X MIC concentrations for all *S. aureus* strains. The compound **2a** demonstrated the highest biofilm disrupting activity that was comparable to vancomycin (Figure 3). Collectively, these results demonstrate that novel thiazole derivatives **2a**–**c** can alter *S. aureus* biofilm integrity, while compound **2a** demonstrates the highest biofilm disrupting activity that is comparable or greater to vancomycin (Figure 3).

## 3. Materials and Methods

### 3.1. Synthesis

Reagents and solvents were obtained from Sigma–Aldrich (St. Louis, MO, USA) and used without further purification. The reaction course and purity of the synthesized compounds were monitored by TLC using aluminum plates precoated with Silica gel with F254 nm (Merck KGaA, Darmstadt, Germany). Melting points were determined with a B-540 melting point analyzer (Büchi Corporation, New Castle, DE, USA) and were uncorrected. NMR spectra were recorded on a Brucker Avance III (400, 101 MHz) spectrometer (Bruker BioSpin AG, Fällanden, Switzerland). Chemical shifts were reported in (*δ*) ppm relative to tetramethylsilane (TMS) with the residual solvent as internal reference (DMSO-*d_6_*, *δ* = 2.50 ppm for ^1^H and *δ* = 39.5 ppm for ^13^C). Data were reported as follows: chemical shift, multiplicity, coupling constant (Hz), integration, and assignment. IR spectra (ν, cm^−1^) were recorded on a Perkin–Elmer Spectrum BX FT–IR spectrometer (Perkin–Elmer Inc., Waltham, MA, USA) using KBr pellets. Elemental analyses (C, H, N) were conducted using the Elemental Analyzer CE-440 (Exeter Analytical, Inc., Chelmsford, MA, USA); their results were found to be in good agreement (±0.3%) with the calculated values.

*3-(1-(4-(phenylamino)phenyl)thioureido)propanoic acid* **(1).** A solution of thioxo tetrahydro pyrimidinone [35] (0.1 mol, 29.7 g) in aqueous 10% sodium hydroxide (200 mL) was boiled, then left to cool down to room temperature, filtered off, and the obtained filtrate was acidified with acetic acid to pH 6. The formed precipitate was filtered off, washed with water, and dried to give the title compound **1** (white solid, yield 20.2 g (64%), m.p. 195 °C (decomp.).

^1^H-NMR (400 MHz, DMSO-*d_6_*): *δ* = 2.55 (t, *J* = 7.9 Hz, 2H, CH_2_CO), 4.18 (t, *J* = 7.9 Hz, 2H, NCH_2_), 6.45–7.68 (m, 11H, H_Ar_ + NH_2_), 8.35 (s, 1H, NH), 12.24 (s, 1H, COOH) ppm.

^13^C-NMR (101 MHz, DMSO-*d_6_*): *δ* = 32.69 (*C*H_2_CO), 50.91 (NCH_2_), 117.47, 117.61, 118.05, 120.86, 128.74, 128.92, 129.20, 129.59, 129.67, 133.00, 143.23, 143.89, 172.97, 182.18 (C_Ar_, C=O, C=S) ppm.

IR (KBr): ν_max_ = 1704 (C=O); 3287 (NH); 3407 (OH) cm^−1^.

Calcd. for C_16_H_17_N_3_O_2_S, %: C 60.93; H 5.43; N 13.32. Found, %: C 60.74; H 5.52; N 13.17.

*General procedure for the preparation of thiazoles***2a**–**c**. A suspension of compound **1** (2.2 mmol) in aqueous 5% sodium carbonate solution (10 mL) was dissolved in methanol (6 mL), and the appropriate acetophenone (2.7 mmol) was added. The mixture was refluxed for 2 h (**a**, **b**) or 4 h (**c**), the cooled down and acidified with diluted (30%) acetic acid to pH 6. The formed precipitate was filtered off, washed with water and recrystallized from propan-2-ol to give the title compounds **2a** (white solid, yield 0.65 g, 68%, m. p. 124–125 °C), **2b** (white solid, yield 0.87 g, 88%, m. p. 130–132 °C), and **2c** (white solid, yield 0.87 g, 80%, m.p. 120–122 °C).

*3-((4-(4-Fluorophenyl)thiazol-2-yl)(4-(phenylamino)phenyl)amino)propanoic acid***(2a)**. ^1^H-NMR (400 MHz, DMSO-*d_6_*): *δ* = 2.62 (t, *J* = 7.4 Hz, 2H, CH_2_CO), 4.11 (t, *J* = 7.4 Hz, 2H, NCH_2_), 6.88 (t, *J* = 7.3, 1H, H_Ar_), 7.08–7.17 (m, 5H, SCH, H_Ar_), 7.22–7.30 (m, 4H, H_Ar_), 7.44 (d, *J* = 8.5 Hz, 2H, H_Ar_), 7.88 (d, *J* = 8.5 Hz, 2H, H_Ar_), 8.39 (s, 1H, NH) ppm.

^13^C-NMR (101 MHz, DMSO-*d_6_*): *δ* = 32.78 (*C*H_2_CO), 48.75 (NCH_2_), 102.21 (SCH), 115.24, 115.45, 116.99, 117.56, 120.40, 127.56, 127.64, 128.59, 129.25, 131.39, 135.83, 142.73, 143.26, 149.38, 160.35, 162.77 170.03, 172.92 (C_Ar_, C=N, C=O) ppm.

IR (KBr): ν_max_ = 1518 (C=N); 1710 (C=O); 3051–3105 (NH); 3389 (OH) cm^−1^.

Calcd. for C_24_H_20_FN_3_O_2_S, %: C 66.50; H 4.63; N 9.69. Found, %: C 66.73; H 4.60; N 9.40.

*3-((4-(4-Chlorophenyl)thiazol-2-yl)(4-(phenylamino)phenyl)amino)propanoic acid***(2b).**^1^H-NMR (400 MHz, DMSO-*d_6_*): *δ* = 2.62 (t, *J* = 7.4 Hz, 2H, CH_2_CO), 4.11 (t, *J* = 7.4 Hz, 2H, NCH_2_), 6.88 (t, *J* = 7.3, 1H, H_Ar_), 7.05–7.16 (m, 5H, SCH, H_Ar_), 7.16–7.39 (m, 4H, H_Ar_), 7.44 (d, *J* = 8.5 Hz, 2H, H_Ar_), 7.88 (d, *J* = 8.5 Hz, 2H, H_Ar_), 8.39 (s, 1H, NH) ppm.

^13^C-NMR (101 MHz, DMSO-*d_6_*): *δ* = 32.99 (*C*H_2_CO), 48.86 (NCH_2_), 103.24 (SCH), 117.00, 117.57, 120.41, 127.34, 128.54, 128.60, 129.26, 131.81, 133.66, 135.79, 142.73, 143.28, 149.19, 170.10, 173.07 (C_Ar_, C=N, C=O) ppm.

IR (KBr): ν_max_ = 1514 (C=N); 1698 (C=O); 3031–3106 (NH); 3376 (OH) cm^−1^.

Calcd. for C_24_H_20_ClN_3_O_2_S, %: C 64.07; H 4.48; N 9.34. Found, %: C 64.41; H 4.46; N 9.57.

*3-((4-(4-Bromophenyl)thiazol-2-yl)(4-(phenylamino)phenyl)amino)propanoic acid***(2c)**. ^1^H-NMR (400 MHz, DMSO-*d_6_*): *δ* = 2.44, 2.57 (2t, *J* = 7.8, 7.6 Hz, 2H, CH_2_CO), 4.09 (t, *J* = 7.6 Hz, 2H, NCH_2_), 6.75–7.34 (m, 10H, SCH, H_Ar_), 7.58 (d, *J* = 8.2 Hz, 4H, H_Ar_), 7.81 (d, *J* = 8.2 Hz, 4H, H_Ar_), 8.33, 8.36 (2s, 1H, NH) ppm.

^13^C-NMR (101 MHz, DMSO-*d_6_*): *δ* = 33.27 (*C*H_2_CO), 49.04 (NCH_2_), 103.28 (SCH), 116.99, 117.48, 117.54, 120.28, 120.38, 127.64, 128.60, 129.20, 129.25, 131.44, 134.00, 142.73, 143.24, 149.22, 170.10, 173.21 (C_Ar_, C=N, C=O) ppm.

IR (KBr): ν_max_ = 1513 (C=N); 1707 (C=O); 3031–3285 (NH); 3372 (OH) cm^−1^.

Calcd. for C_24_H_20_BrN_3_O_2_S, %: C 58.30; H 4.08; N 8.50. Found, %: C 58.22; H 4.21; N 8.61.

*Methyl 3-((4-(4-chlorophenyl)thiazol-2-yl)(4-(phenylamino)phenyl)amino)propanoate***(3)**. To a boiling solution of compound **2b** (11.1 mmol, 5 g) in methanol (100 mL), a catalytic amount of sulfuric acid (0.6 mL) was added, and the reaction mixture was refluxed for 5 h. Then the volatile fractions were evaporated under reduced pressure, the residue was poured with aqueous 5% sodium carbonate solution (150 mL) and boiled. After cooling, the formed precipitate was filtered off, washed with water and recrystallized from the mixture of methanol and water (1:1) to give the title compound **4** (white solid, yield 4.12 g, 80%, m.p. 73–74 °C).

^1^H-NMR (400 MHz, DMSO-*d_6_*): *δ* = 2.75 (t, *J* = 7.0 Hz, 2H, CH_2_CO), 3.54 (s, 3H, OCH_3_), 4.17 (t, *J* = 7.0 Hz, 2H, NCH_2_), 6.89 (t, *J* = 7.3 Hz, 1H, H_Ar_), 7.08–7.20 (m, 6H, SCH, H_Ar_), 7.24–7.29 (m, 4H, H_Ar_), 7.46 (d, *J* = 8.2 Hz, 2H, H_Ar_), 7.88 (d, *J* = 8.2 Hz, 2H, H_Ar_) ppm.

^13^C-NMR (101 MHz, DMSO-*d_6_*): *δ* = 32.28 (*C*H_2_CO), 48.52 (NCH_2_), 51.43 (OCH_3_), 103.49 (SCH), 116.97, 117.60, 120.47, 127.34, 128.55, 128.61, 129.26, 131.89, 133.45, 135.51, 142.65, 143.43, 148.97, 170.10, 171.60 (C_Ar_, C=N, C=O) ppm.

IR (KBr): ν_max_ = 1596 (C=N); 1734 (C=O); 3107–3193 (NH) cm^−1^.

Calcd. For C_25_H_22_ClN_3_O_2_S, %: C 64.72; H 4.78; N 9.06. Found, %: C 64.67; H 4.79; N 9.17.

*3-((4-(4-Chlorophenyl)thiazol-2-yl)(4-(phenylamino)phenyl)amino)propanehydrazide***(4).** To a solution of ester **3** (20 mmol, 7.12 g) in propan-2-ol (150 mL), hydrazine monohydrate (60 mmol, 3 g) was added dropwise and the mixture was heated at reflux for 18 h. After completion of the reaction the mixture was cooled down and diluted with water (100 mL). The obtained precipitate was filtered off, washed with water, and recrystallized from propan-2-ol to give the title compound **4** (yellow solid, yield 7.52 g, 73%, m.p. 104–105 °C).

^1^H-NMR (400 MHz, DMSO-*d_6_*): *δ* = 2.31–2.50 (2H, CH_2_CO; the signal overlaps with the signal of the DMSO-*d_6_*), 4.12 (t, *J* = 7.4 Hz, 2H, NCH_2_), 4.17 (s, 2H, NH_2_), 6.88 (t, *J* = 7.3 Hz, 1H, H_Ar_), 7.11–7.16 (m, 5H, SCH, H_Ar_), 7.24–7.29 (m, 4H, H_Ar_), 7.45 (d, *J* = 8.2 Hz, 2H, H_Ar_), 7.90 (d, *J* = 8.2 Hz, 2H, H_Ar_), 8.38 (s, 1H, NH), 9.10 (s, 1H, N*H*NH_2_) ppm.

^13^C-NMR (101 MHz, DMSO-*d_6_*): *δ* = 31.99 (*C*H_2_CO), 49.22 (NCH_2_), 103.26 (SCH), 116.97, 117.55, 120.40, 127.38, 128.50, 128.57, 129.25, 131.80, 133.64, 135.85, 142.71, 143.25, 149.19, 169.44, 170.03 (C_Ar_, C=N, C=O) ppm.

IR (KBr): ν_max_ = 1510 (C=N); 1651 (C=O); 3106–3180 (NH) cm^−1^.

Calcd. For C_24_H_22_ClN_5_OS, %: C 62.13; H 4.78; N 15.09. Found, %: C 61.26; H 4.87; N 14.88.

*General procedure for the preparation of hydrazones***5a**–**i.** To a boiling solution of hydrazide **4** (5 mmol, 2.32 g) in propan-2-ol (35 mL) the corresponding aromatic aldehyde (6 mmol) and a catalytic amount of acetic acid (0.4 mL) were added, and the mixture was heated at reflux for 2 h after, cooled down, the formed solid was filtered off, washed with propan-2-ol, diethyl ether, and recrystallized from propan-2-ol to give the appropriate title compound **5**.

*N-benzylidene-3-((4-(4-chlorophenyl)thiazol-2-yl)(4-(phenylamino)phenyl)amino)propanehydrazide***(5a).** White solid, yield 2.24 g, 81%, m.p. 162–164 °C.

^1^H-NMR (400 MHz, DMSO-*d_6_*): *δ* = (*Z/E* 65/35), 2.67, 3.10 (2t, *J* = 7.1, 7.4 Hz, 2H, CH_2_CO), 4.24 (q, *J* = 7.7 Hz, 2H, NCH_2_), 6.85–6.91 (m, 1H, H_Ar_), 7.08–7.48 (m, 16H, SCH, H_Ar_), 7.86–8.13 (m, 3H, H_Ar_+N=CH), 8.38, 8.40 (2s, 1H, NH), 11.35, 11.45 (2s, 1H, NH) ppm.

^13^C-NMR (101 MHz, DMSO-*d_6_*): *δ* = 31.04, 33.01, 48.99 (*C*H_2_CO, NCH_2_), 103.25 (SCH), 116.88, 116.95, 117.54, 117.65, 120.45, 126.63, 126.97, 127.31, 128.47, 128.64, 128.67, 129.23, 129.60, 129.89, 129.92, 131.74, 131.79, 133.61, 133.64, 134.18, 135.78, 135.85, 142.67, 142.77, 143.33, 146.06, 149.18, 166.65, 169.98, 170.09, 172.47 (C_Ar_, CH=N, C=O) ppm.

IR (KBr): ν_max_ = 1513 (C=N); 1660 (C=O); 3031–3180 (NH) cm^−1^.

Calcd. for C_31_H_26_ClN_5_OS, %: C 67.44; H 4.75; N 12.69. Found, %: C 66.57; H 4.67; N 12.46.

*3-((4-(4-Chlorophenyl)thiazol-2-yl)(4-(phenylamino)phenyl)amino)-N′-(4-fluorobenzylidene)propanehydrazide* (**5b**). White solid, yield 2.39 g, 84%, m.p. 178–180 °C.

^1^H-NMR (400 MHz, DMSO-*d_6_*): *δ* = (*Z/E* 65/35), 2.67, 3.10 (2t, *J* = 7.1, 7.4 Hz, 2H, CH_2_CO), 4.16–4.32 (m, 2H, NCH_2_), 6.84–6.91 (m, 1H, H_Ar_), 7.08–7.17 (m, 6H, SCH, H_Ar_), 7.21–7.35 (m, 5H, H_Ar_), 7.41 (dd, *J* = 16.7, 8.2 Hz, 2H, H_Ar_), 7.52–7.73 (m, 2H, H_Ar_), 7.86–8.14 (m, 3H, H_Ar_+N=CH), 8.38, 8.42 (2s, 1H, NH), 11.37, 11.46 (2s, 1H, NH) ppm.

^13^C-NMR (101 MHz, DMSO-*d_6_*): *δ* = 30.99, 33.00, 48.90 49.01 (*C*H_2_CO, NCH_2_), 103.26 (SCH), 115.54, 115.75, 116.84, 116.95, 117.54, 117.66, 120.48, 127.30, 128.47, 128.58, 128.65, 128.68, 128.77, 129.23, 130.78, 131.77, 133.60, 133.63, 135.82, 141.60, 142.62, 142.67, 143.35, 144.93, 149.19, 161.53, 163.49, 166.68, 170.08, 172.44 (C_Ar_, CH=N, C=O) ppm.

IR (KBr): ν_max_ = 1509 (C=N); 1652 (C=O), 3030–3068 (NH) cm^−1^.

Calcd. for C_31_H_25_ClFN_5_OS, %: C 65.31; H 4.42; N 12.29. Found, %: C 65.17; H 4.58; N 12.05.

*3-((4-(4-Chlorophenyl)thiazol-2-yl)(4-(phenylamino)phenyl)amino)-N′-(4-chlorobenzylidene)propanehydrazide* (**5c**). White solid, yield 2.58 g, 88%, m.p. 201–203 °C.

^1^H-NMR (400 MHz, DMSO-*d_6_*): *δ* = (*Z/E* 65/35), 2.68, 3.10 (2t, *J* = 7.1, 7.4 Hz, 2H, CH_2_CO), 4.15–4.30 (m, 2H, NCH_2_), 6.85–6.92 (m, 1H, H_Ar_), 7.12–7.68 (m, 15H, SCH, H_Ar_), 7.84–8.13 (m, 2H, H_Ar_+N=CH), 8.37, 8.42 (2s, 1H, NH), 11.42, 11.52 (2s, 1H, NH) ppm.

^13^C-NMR (101 MHz, DMSO-*d_6_*): *δ* = 30.99, 33.02, 48.99 (*C*H_2_CO, NCH_2_), 103.28 (SCH), 116.80, 116.94, 117.54, 117.70, 120.40, 120.50, 127.30, 127.37, 128.20, 128.47, 128.58, 128.67, 128.70, 128.82, 129.23, 131.78, 133.10, 133.26, 133.62, 134.04, 134.31, 135.77, 141.46, 142.59, 143.38, 144.72, 149.19, 166.76, 169.95, 170.07, 172.50 (C_Ar_, CH=N, C=O) ppm.

IR (KBr): ν_max_ = 1509 (C=N); 1656 (C=O); 3029–3065 (NH) cm^−1^.

Calcd. for C_31_H_25_Cl_2_N_5_OS, %: C 63.48; H 4.30; N 11.94. Found, %: C 63.17; H 4.50; N 11.81.

*N′-(4-bromobenzylidene)-3-((4-(4-chlorophenyl)thiazol-2-yl)(4-(phenylamino)phenyl)amino)propanehydrazide* (**5d**) White solid, yield 2.78 g, 88%, m.p. 182–184 °C.

^1^H-NMR (400 MHz, DMSO-*d_6_*): *δ* = (*Z/E* 70/30), 2.68, 3.09 (2t, *J* = 7.1, 7.5 Hz, 2H, CH_2_CO), 4.16–4.29 (m, 2H, NCH_2_), 6.86–6.91 (m, 1H, H_Ar_), 7.10–7.45 (m, 14H, SCH, H_Ar_), 7.61 (s, 1H, H_Ar_), 7.84–8.12 (m, 3H, H_Ar_, N=CH), 8.37, 8.43 (2s, 1H, NH), 11.42, 11.52 (2s, 1H, NH) ppm.

^13^C-NMR (101 MHz, DMSO-*d_6_*): *δ* = 30.98, 33.03, 48.99 (*C*H_2_CO, NCH_2_), 103.28 (SCH), 116.80, 116.94, 117.54, 120.90, 122.81, 127.30, 128.43, 128.47, 128.58, 128.67, 128.82, 129.24, 131.62, 131.73, 131.78, 133.43, 133.62, 135.76, 141.56, 142.58, 143.38, 144.80, 149.19, 166.77, 169.96, 172.50 (C_Ar_, CH=N, C=O) ppm.

IR (KBr): ν_max_ = 1509 (C=N); 1656 (C=O); 3029–3063 (NH) cm^−1^.

Calcd. for C_31_H_25_BrClN_5_OS, %: C 59.01; H 3.99; N 11.10. Found, %: C 59.03; H 3.22; N 10.88.

*3-((4-(4-Chlorophenyl)thiazol-2-yl)-N′-(4-nitrobenzylidene)-(4-(phenylamino)phenyl)amino)propanehydrazide* (**5e**). White solid, yield 2.36 g, 79%, m.p. 205–207 °C.

^1^H-NMR (400 MHz, DMSO-*d_6_*): *δ* = (*Z/E* 65/35), 2.71, 3.14 (2t, *J* = 7.0, 7.3 Hz, 2H, CH_2_CO), 4.12–4.36 (m, 2H, NCH_2_), 6.87 (t, *J* = 7.4 Hz, 1H, H_Ar_), 6.98–7.52 (m, 11H, SCH, H_Ar_), 7.73 (d, *J* = 8.5 Hz, 1H, H_Ar_), 7.82–8.45 (m, 7H, H_Ar_, N=CH, NH), 11.67, 11.75 (2s, 1H, NH) ppm.

^13^C-NMR (101 MHz, DMSO-*d_6_*): *δ* = 30.83, 32.47, 48.96 (*C*H_2_CO, NCH_2_), 103.33 (SCH), 116.86, 117.53, 117.60, 120.46, 123.82, 123.96, 127.29, 127.36, 127.46, 127.86, 128.47, 128.63, 129.21, 131.79, 133.60, 135.74, 140.46, 142.59, 143.35, 147.46, 149.18, 169.96, 172.91 (C_Ar_, CH=N, C=O) ppm.

IR (KBr): ν_max_ = 1513 (C=N); 1666 (C=O); 3029–3076 (NH) cm^−1^.

Calcd. for C_31_H_25_ClN_6_O_3_S, %: C 62.36; H 4.22; N 14.08. Found, %: C 62.25; H 4.25; N 13.97.

*3-((4-(4-Chlorophenyl)thiazol-2-yl)-N′-(4-(dimethylamino)benzylidene)-(4-(phenylamino)phenyl)amino)propanehydrazide* (**5f**). White solid, yield 2.38 g, 80%, m.p. 191–193 °C.

^1^H-NMR (400 MHz, DMSO-*d_6_*): *δ* = (*Z/E* 65/35), 2.63, 3.07 (2t, *J* = 7.0, 7.3 Hz, 2H, CH_2_CO), 2.85, 2.95 (2s, 6H, 2CH_3_), 4.15–4.28 (m, 2H, NCH_2_), 6.56, 6.72 (2d, *J* = 8.4 Hz, 2H, H_Ar_), 6.87 (t, *J* = 7.2 Hz, 1H, H_Ar_), 7.11–7.46 (m, 13H, SCH, H_Ar_), 7.80–8.00 (m, 3H, H_Ar_, N=CH), 8.38, 8.44 (2s, 1H, NH), 11.06, 11.15 (2s, 1H, NH) ppm.

^13^C-NMR (101 MHz, DMSO-*d_6_*): *δ* = 31.12, 32.94, 39.63, 49.09 (*C*H_2_CO, NCH_2_, 2CH_3_), 103.23 (SCH), 111.65, 111.75, 117.01, 117.37, 117.54, 120.31, 121.63, 127.32, 127.38, 127.87, 128.28, 128.48, 128.58, 128.67, 129.23, 131.72, 133.66, 135.92, 142.70, 143.20, 143.52, 146.91, 149.20, 151.11, 165.97, 169.93, 171.78 (C_Ar_, CH=N, C=O) ppm.

IR (KBr): ν_max_ = 1511 (C=N); 1668 (C=O); 3055–3169 (NH) cm^−1^.

Calcd. for C_33_H_31_ClN_6_OS, %: C 66.60; H 5.25; N 14.12. Found, %: C 66.29; H 5.22; N 13.88.

*3-((4-(4-Chlorophenyl)thiazol-2-yl)-N′-(4-methylbenzylidene)-(4-(phenylamino)phenyl)amino)propanehydrazide* (**5g**). White solid, yield 2.69 g, 95%, m.p. 176–178 °C.

^1^H-NMR (400 MHz, DMSO-*d_6_*): *δ* = (*Z/E* 65/35), 2.25, 2.32 (2s, 3H, CH_3_), 2.67, 3.08 (2t, *J* = 6.9, 7.5 Hz, 2H, CH_2_CO), 4.22 (q, *J* = 6.4 Hz, 2H, NCH_2_), 6.88 (t, *J* = 7.1 Hz, 1H, H_Ar_), 7.08–7.17 (m, 6H, SCH, H_Ar_), 7.22–7.55 (m, 9H, H_Ar_), 7.84–8.10 (m, 3H, H_Ar_, N=CH), 8.37, 8.42 (2s, 1H, NH), 11.28, 11.38 (2s, 1H, NH) ppm.

^13^C-NMR (101 MHz, DMSO-*d_6_*): *δ* = 20.96, 31.09, 32.89, 49.01 (CH_3_, *C*H_2_CO, NCH_2_), 103.25 (SCH), 116.85, 116.95, 117.54, 117.62, 120.43, 126.59, 126.95, 127.32, 127.38, 128.49, 128.58, 128.67, 129.23, 129.28, 129.35, 131.47, 131.74, 133.64, 135.81, 139.31, 142.64, 142.86, 143.34, 149.19,169.95, 172.28 (C_Ar_, CH=N, C=O) ppm.

IR (KBr): ν_max_ = 1511 (C=N); 1652 (C=O); 3029–3126 (NH) cm^−1^.

Calcd. for C_32_H_28_ClN_5_OS, %: C 67.89; H 4.99; N 12.37. Found, %: C 67.78; H 5.11; N 12.35.

*3-((4-(4-Chlorophenyl)thiazol-2-yl)(4-(phenylamino)phenyl)amino)-N’-(thiophen-2-ylmethylene)propanehydrazide* (**5h**). White solid, yield 1.90 g, 68%, m.p. 178–180 °C.

^1^H-NMR (400 MHz, DMSO-*d_6_*): *δ* = (*Z/E* 55/45), 2.65, 3.00 (2t, *J* = 7.1, 7.3 Hz, 2H, CH_2_CO), 4.18–4.28 (m, 2H, NCH_2_), 6.87 (t, *J* = 7.3 Hz, 1H, H_Ar_), 7.08–7.17 (m, 6H, SCH, H_Ar_), 7.23–7.32 (m, 4H, H_Ar_), 7.36–7.45 (m, 3H, H_Ar_), 7.52, 7.63 (2d, *J* = 5.1 Hz, 1H, H_Ar_), 7.84–7.92 (m, 2H, H_Ar_), 8.14, 8.36 (2s, 1H, N=CH), 8.38 (s, 1H, NH), 11.32, 11.40 (2 s, 1H, NH) ppm.

^13^C-NMR (101 MHz, DMSO-*d_6_*): *δ* = 30.84, 33.00, 48.62, 48.87 (*C*H_2_CO, NCH_2_), 103.23, 103.35 (SCH), 116.94, 117.04, 117.48, 117.55, 120.35, 127.34, 127.75, 127.84, 128.02, 128.44, 128.49, 128.60, 128.68, 129.23, 129.93, 130.67, 131.73, 131.79, 133.60, 133.64, 135.78, 137.87, 139.06, 139.08, 141.30, 142.66, 142.73, 143.24, 143.28, 149.17, 166.51, 170.07, 172.09 (C_Ar_, CH=N, C=O) ppm.

IR (KBr): ν_max_ = 1510 (C=N); 1687 (C=O), 3029–3116 (NH) cm^−1^.

Calcd. for C_29_H_24_ClN_5_OS_2_, %: C 62.41; H 4.33; N 12.55. Found, %: C 62.39; H 4.30; N 12.59.

*3-((4-(4-Chlorophenyl)thiazol-2-yl)(4-(phenylamino)phenyl)amino)-N’-((5-nitrothiophen-2-yl)methylene)propanehydrazide* (**5i**). White solid, yield 2.20 g, 73%, m.p. 186–188 °C.

^1^H-NMR (400 MHz, DMSO-*d_6_*): *δ* = (*Z/E* 60/40), 2.69, 3.06 (2t, *J* = 7.0, 7.1 Hz, 2H, CH_2_CO), 4.24 (q, *J* = 7.4 Hz, 2H, NCH_2_), 6.87 (t, *J* = 7.3 Hz, 1H, H_Ar_), 7.05–7.54 (m, 13H, SCH, H_Ar_), 7.82–7.91 (m, 2H, H_Ar_), 8.04, 8.09 (2d, *J* = 4.3, 4.4 Hz, 1H, H_Ar_), 8.13 (s, 0.6H, N=CH), 8.36, 8.37, 8.40 (3s, 1H, NH+0.4H, N=CH), 11.76, 11.81 (2s, 1H, NH) ppm.

^13^C-NMR (101 MHz, DMSO-*d_6_*): *δ* = 30.73, 33.21, 48.67 (*C*H_2_CO, NCH_2_), 103.26, 103.36 (SCH), 116.90, 117.53, 117.55, 120.37, 127.28, 127.35, 128.39, 128.47, 128.54, 128.77, 129.19, 129.35, 130.41, 131.75, 131.79, 133.56, 133.60, 135.71, 136.13, 139.64, 142.66, 143.29, 146.70, 149.18, 150.28, 167.35, 170.03, 172.80 (C_Ar_, CH=N, C=O) ppm.

IR (KBr): ν_max_ = 1508 (C=N); 1681 (C=O), 3028–3107 (NH) cm^−1^.

Calcd. for C_29_H_23_ClN_6_O_3_S_2_, %: C 57.75; H 3.84; N 13.93. Found, %: C 57.59; H 3.85; N 13.76.

*General procedure for the preparation of hydrazones***5j**, **k.** A mixture of hydrazide **4** (5 mmol, 2.32 g) and the corresponding ketone (acetone or 2-butanone) (35 mL) was heated at reflux for 5 h. After completion of the reaction, the mixture was cooled down, diluted with water (35 mL), and left in refrigerator for 24 h. Then the formed precipitate was filtered off, washed with acetone, diethyl ether and recrystallized from acetone to give the title compound **5j**. Product **5k** was separated from the reaction mixture by evaporating the volatile fractions under reduced pressure, diluting the residue with diethyl ether, filtering the obtained solid, and recrystallizing it from 2-butanone to give the title compound **5k**.

*3-((4-(4-Chlorophenyl)thiazol-2-yl)(4-(phenylamino)phenyl)amino)-N’-(propan-2-ylidene)propanehydrazide* (**5j**). White solid, yield 2.07 g, 82%, m.p. 76–78 °C.

^1^H-NMR (400 MHz, DMSO-*d_6_*): *δ* = (*Z/E* 60/40), 1.80, 1.85, 1.89 (3s, 6H, 2CH_3_), 2.66, 2.95 (2t, *J* = 7.3, 7.5 Hz, 2H, CH_2_CO), 4.17 (t, *J* = 7.5 Hz, 2H, NCH_2_), 6.88 (t, *J* = 7.3 Hz, 1H, H_Ar_), 7.09–7.17 (m, 5H, SCH, H_Ar_), 7.23–7.31 (m, 4H, H_Ar_), 7.44 (d, *J* = 8.3 Hz, 2H, H_Ar_), 7.84–7.93 (m, 2H, H_Ar_), 8.37, 8.38 (2s, 1H, NH), 10.02, 10.06 (2s, 1H, NH) ppm.

^13^C-NMR (101 MHz, DMSO-*d_6_*): *δ* = 17.01, 17.49, 24.95, 25.14, 31.01, 32.46, 48.72, 48.89 (2CH_3_, *C*H_2_CO, NCH_2_), 103.15, 103.26(SCH), 116.97, 117.01, 117.47, 117.52, 120.35, 127.30, 127.37, 128.43, 128.50, 128.56, 129.24, 131.79, 133.64, 135.76, 135.91, 142.76, 143.13, 143.24, 149.15, 150.19, 154.83, 166.49, 169.89, 170.10, 172.48 (C_Ar_, C=N, C=O) ppm.

IR (KBr): ν_max_ = 1513 (C=N); 1667 (C=O), 3031–3107 (NH) cm^−1^.

Calcd. for C_27_H_26_ClN_5_OS, %: C 64.34; H 5.20; N 13.89. Found, %: C 64.56; H 5.28; N 13.94.

*N’-(butan-2-ylidene)-3-((4-(4-chlorophenyl)thiazol-2-yl)(4-(phenylamino)phenyl)amino)propanehydrazide* (**5k**). White solid, yield 1.76 g, 68%, m.p. 79–81 °C.

^1^H-NMR (400 MHz, DMSO-*d_6_*): *δ* = (*Z/E* 60/40), 0.76–1.01 (m, 3H, CH_2_C*H*_3_), 1.79, 1.83, 1.87 (3s, 3H, CH_3_), 2.11–2.24 (m, 2H, CH_3_C*H*_2_), 2.67, 2.96 (2t, *J* = 7.4, 7.5 Hz, 2H, CH_2_CO), 4.13–4.24 (m, 2H, NCH_2_), 6.89 (t, *J* = 7.3 Hz, 1H, H_Ar_), 7.10–7.16 (m, 5H, SCH, H_Ar_), 7.27 (t, *J* = 7.8 Hz, 4H, H_Ar_), 7.44 (d, *J* = 8.2 Hz, 2H, H_Ar_), 7.88 (t, *J* = 8.2 Hz, 2H, H_Ar_), 8.37, 8.38 (2s, 1H, NH), 9.99, 10.07, 10.12, 10.16 (4s, 1H, NH) ppm.

^13^C-NMR (101 MHz, DMSO-*d_6_*): *δ* = 10.37, 10.83, 13.97, 15.78, 15.90, 20.60, 22.07, 24.79, 26.35, 30.96, 31.40, 31.53, 32.52, 33.93, 34.20, 48.72, 48.92 (2CH_3_, CH_3_*C*H_2_, *C*H_2_CO, NCH_2_), 103.15 (SCH), 117.00, 117.45, 117.52, 120.34, 127.29, 127.37, 128.48, 128.54, 129.23, 131.78, 133.63, 133.67, 135.86, 142.76, 143.17, 149.16, 153.55, 158.35, 166.55, 169.98, 172.67 (C_Ar_, C=N, C=O) ppm.

IR (KBr): ν_max_ = 1512 (C=N); 1667 (C=O), 3031–3099 (NH) cm^−1^.

Calcd. for C_28_H_28_ClN_5_OS, %: C 64.91; H 5.45; N 13.52. Found, %: C 64.57; H 5.37; N 13.88.

*3-((4-(4-Chlorophenyl)thiazol-2-yl)(4-(phenylamino)phenyl)amino)-1-(3,5-dimethyl-1H-pyrazol-1-yl)propan-1-one* (**6**). To a solution of hydrazide **4** (5 mmol, 2.32 g) in propan-2-ol (35 mL), 2,4-pentanedione (10 mmol, 1 g) and hydrochloric acid (2 drops) were added dropwise and the mixture was refluxed for 9 h, then cooled down, and the formed precipitate was filtered off washed with propan-2-ol and recrystallized from propan-2-ol to give the title compound **7** (white solid, 1.95 g, 74%, m.p. 105–106 °C).

^1^H-NMR (400 MHz, DMSO-*d_6_*): *δ* = 2.16, 2.36 (2s, 6H, 2CH_3_), 2,72–3.02 (m, 2H, CH_2_CO), 4.05–4.35 (m, 2H, NCH_2_), 6.14, 6.26 (2s, 1H, CH), 6.88 (t, *J* = 6.8 Hz, 1H, H_Ar_), 7.09–7.19 (m, 5H, SCH, H_Ar_), 7.24–7.32 (m, 4H, H_Ar_), 7.45 (d, *J* = 8.0 Hz, 2H, H_Ar_), 7.88 (d, *J* = 8.0 Hz, 2H, H_Ar_), 8.37 (s, 1H, NH) ppm.

^13^C-NMR (101 MHz, DMSO-*d_6_*): *δ* = 15.79, 15.85 (2CH_3_); 32.88 (*C*H_2_CO), 48.57 (NCH_2_); 103.16 (SCH), 110.10 (CH), 117.01, 117.49, 120.37, 127.28, 128.52, 129.24, 131.79, 133.65, 135.82, 142.74, 143.17, 149.10, 154.21, 168.29, 169.97 (C_Ar_, C=N, C=O) ppm

IR (KBr): ν_max_ = 1513 (C=N); 1647 (C=O), 3031–3110 (NH) cm^−1^.

Calcd. for C_29_H_26_ClN_5_OS, %: C 65.96; H 4.96; N 13.26. Found, %: C 66.03; H 5.07; N 13.34.

*2-(3-((4-(4-Chlorophenyl)thiazol-2-yl)(4-(phenylamino)phenyl)amino)-N-(2,5-dimethyl-1H-pyrrol-1-yl)propanamide* (**7**). To a solution of hydrazide **4** (5 mmol, 2.32 g) in propan-2-ol (45 mL) 2,5-hexanedione (9 mmol, 1.03 g) and acetic acid (dropwise, 0.75 mL) were added and the mixture was refluxed for 3 h, then cooled down and diluted with water (30 mL). The formed crystalline solid was filtered off, washed with water and recrystallized from propan-2-ol to give the title compound **7** (white solid, 2.17 g, 80%, m.p. 146–147 °C).

^1^H-NMR (400 MHz, DMSO-*d_6_*): *δ* = 1.94, 2.01 (2s, 6H, 2CH_3_), 2.77 (t, *J* = 6.9 Hz, 2H, CH_2_CO), 4.25 (t, *J* = 6.8 Hz, 2H, NCH_2_), 5.61 (s, 2H, CH-CH), 6.89 (t, *J* = 7.1 Hz, 1H, H_Ar_), 7.12–7.34 (m, 10H, SCH, H_Ar_), 7.45 (d, *J* = 8.1 Hz, 2H, H_Ar_), 7.91 (d, *J* = 8.1 Hz, 2H, H_Ar_), 8.37, 8.40 (2s, 1H, NH), 10.68 (s, 1H, NH) ppm.

^13^C-NMR (101 MHz, DMSO-*d_6_*): *δ* = 10.91, 11.02 (2CH_3_), 31.75 (*C*H_2_CO), 48.66 (NCH_2_), 102.90 (CH-CH), 103.46 (SCH), 116.99, 117.45, 117.55, 120.43, 126.69, 127.36, 128.49, 128.54, 129.25, 131.84, 133.61, 135.73, 142.69, 143.32, 149.13, 169.71, 170.10 (C_Ar_, C=N, C=O) ppm.

IR (KBr): ν_max_ = 1513 (C=N); 1679 (C=O), 3028–3105 (NH) cm^−1^.

Calcd. for C_30_H_28_ClN_5_OS, %: C 66.47; H 5.21; N 12.92. Found, %: C 66.36; H 5.31; N 12.75.

*2-(3-((4-(4-Chlorophenyl)thiazol-2-yl)(4-(phenylamino)phenyl)amino)propanoyl)-N-phenylhydrazine-1-carbothioamide* (**8**). To a solution of hydrazide **4** (5 mmol, 2.32 g) solution in methanol (100 mL), a solution of phenyl isothiocyanate (5 mmol, 0.6 mL) in methanol (5 mL) was added dropwise and the mixture was refluxed for 2 h. After completion of the reaction, the mixture was cooled down and diluted with water (80 mL). The formed solid was filtered off, washed with water, and recrystallized from propan-2-ol to give the title compound **8** (white solid, 2.79 g, 93%, m.p. 116–118 °C).

^1^H-NMR (400 MHz, DMSO-*d_6_*): *δ* = 2.68 (t, *J* = 7.5 Hz, 2H, CH_2_CO), 4.18 (t, *J* = 6.8 Hz, 2H, NCH_2_), 6.89 (t, *J* = 7.2 Hz, 1H, H_Ar_), 7.09–7.20 (m, 6H, SCH, H_Ar_), 7.22–7.41 (m, 8H, H_Ar_), 7.45 (d, *J* = 8.1 Hz, 2H, H_Ar_), 7.90 (d, *J* = 8.1 Hz, 2H, H_Ar_), 8.39 (s, 1H, NH), 9.54 (s, 1H, NH), 9.59 (br. s, 1H, NH), 9.99 (s, 1H, NH) ppm.

^13^C-NMR (101 MHz, DMSO-*d_6_*): *δ* = 31.73 (*C*H_2_CO), 48.41 (NCH_2_), 103.35 (SCH), 117.01, 117.57, 120.44, 125.14, 125.19, 125.86, 126.00, 127.35, 128.03, 128.51, 128.71, 129.26, 131.83, 133.61, 135.76, 139.10, 142.68, 143.36, 149.19, 170.16, 180.82 (C_Ar_, C=N, C=O, C=S) ppm.

IR (KBr): ν_max_ = 1512 (C=N); 1681 (C=O), 3029–3108 (NH) cm^−1^.

Calcd. for C_31_H_27_ClN_6_OS_2_, %: C 62.14; H 4.54; N 14.03. Found, %: C 61.98; H 4.64; N 13.91.

*5-(2-((4-(4-Chlorophenyl)thiazol-2-yl)(4-(phenylamino)phenyl)amino)ethyl)-4-phenyl-2,4-dihydro-3H-1,2,4-triazole-3-thione* (**9**). A mixture of compound **8** (5 mmol, 3 g) and aqueous 4 % NaOH solution (150 mL) was heated at reflux for 3 h, then cooled down and acidified with acetic acid to pH 6. The formed precipitate was filtered off, washed with water and recrystallized from propan-2-ol to give the title compound **9** (white solid, 2.41 g, 83%, m.p. 119–121 °C).

^1^H-NMR (400 MHz, DMSO-*d_6_*): *δ* = 2.93 (t, *J* = 6.8 Hz, 2H, CH_2_), 3.98 (t, *J* = 6.8 Hz, 2H, NCH_2_), 6.88 (t, *J* = 7.2 Hz, 1H, H_Ar_), 7.05–7.15 (m, 7H, SCH, H_Ar_), 7.25–7.32 (m, 4H, H_Ar_), 7.43–7.53 (m, 5H, H_Ar_), 7.77 (d, *J* = 8.2 Hz, 2H, H_Ar_), 8.40 (s, 1H, NH), 13.70 (br. s, 1H, NH) ppm.

^13^C-NMR (101 MHz, DMSO-*d_6_*): *δ* = 23.97 (CH_2_), 49.57 (NCH_2_), 103.45 (SCH), 117.00, 117.58, 120.48, 127.35, 128.20, 128.38, 128.51, 129.26, 129.35, 129.39, 131.84, 133.47, 133.73, 135.38, 142.63, 143.35, 149.18, 149.94, 167.70, 169.82 (C_Ar_, C=N, C=S) ppm.

IR (KBr): ν_max_ = 1090 (C=S); 1529 (C=N); 3037–3108 (NH) cm^−1^.

Calcd. for C_31_H_25_ClN_6_S_2_, %: C 64.07; H 4.34; N 14.46. Found, %: C 63.93; H 4.32; N 14.78.

### 3.2. In Silico ADME Prediction

The in silico absorption distribution, metabolism, and excretion (ADME) properties were predicted by using SwissADME software [65]. The SMILE structures were generated and used for ADME prediction. The physicochemical properties such as molecular weight, number of aromatic heavy atoms, Csp3 fraction, number of rotatable bonds, number of hydrogen bond acceptors and donors, molar refractivity, and total polar surface (TPSA) were predicted. The pharmacological properties, such as consensus lipophilicity (Log P_o/w_), water solubility (mol/L), gastrointestinal absorption, permeability through the brain–blood barrier, interactions with P-glycoprotein 1 (Pgp) and cytochromes, and skin permeability (Log Kp) were estimated.

### 3.3. Microbial Strains and Culture Conditions

The in vitro antimicrobial activity of compounds **1**–**9** was determined using laboratory collection and medically important bacterial and fungal (Supporting Information, Table S1) pathogens as well as Center for Disease Control ARisolate bank panels. Prior to the study, all strains were maintained in commercial cryopreservation systems at −80 °C. Bacterial strains were subcultured on Columbia Sheep Blood agar (Becton Dickenson, Franklin Lakes, NJ, United States). Fungal pathogens were cultured on Sabouraud Chloramphenicol agar (Liofilchem, Roseto degli Abruzz, Italy).

### 3.4. Minimal Inhibitory Concentration (MIC) Determination

The antimicrobial activity of compounds **1**–**9** was evaluated by using the broth microdilution method as suggested by Clinical Laboratory Standards Institute (CLSI), with brief modifications [75]. Briefly, compounds were dissolved in dimethylsulfoxide (DMSO) to achieve a final concentration of 25–30 mg/mL. Series of dilutions were prepared in deep 96-well microplates to achieve 2× concentration of 0.25, 0.5, 1, 2, 4, 8, 16, and 64 µg/mL using CAMHB (for bacteria), RPMI/MOPS (for fungal pathogens). The microplates, containing series of dilutions, were inoculated with fresh cultures of each tested organism to achieve the final concentration of 5 × 10^4^ CFU of test organism in media containing 1% DMSO and 1× drug concentration in a volume of 200 µL per well. The inoculated wells, containing media with 1% DMSO, were used as a positive control. The microplates were sealed with plate-sealing tape and incubated at 35 ± 1 °C, for 18 ± 2 h, 24 h, and 48 h for fungal pathogens.

After incubation, the plates were read using manual microplate viewer (Sesititre Manual Viewbox, Thermo Fisher Scientific, Waltham, MA, United States). Minimal inhibitory concentration (MIC) was defined as lowest concentration (µg/mL) of a tested drug that fully inhibits growth of the test organism. All experiments were performed in repeatedly with three technical replicates.

### 3.5. Evaluation of Cytotoxicity in Vero Cells

Cytotoxicity of the compounds was evaluated using MTT assay. Vero African green monkey kidney cells were exposed to the 100 µg/mL of each compound or vancomycin and daptomycin (control) for 48 h (37 °C; 5% CO_2_) in Eagle’s minimum essential medium supplemented with 10% FBS, 1X PenStrept, 1X Glutamax. After incubation, the MTT was added, and viability was determined by measuring the absorbance of the reduced formazan at 570 and 600 nm. The absorbance measured at 600 nm was automatically subtracted from the absorbance at 570 nm to eliminate the effects of nonspecific absorption. The percent cytotoxicity achieved by the compounds was calculated according to standard methods and normalized to untreated control (UC).

### 3.6. In Vitro Time-Kill Study

In vitro time-kill studies were performed using 5 mL of representative *Staphylococcus aureus* strains harboring genetically defined resistance mechanisms. The bacteria were cultured overnight in 10 mL of CAMHB. The inoculum was further diluted (1:10) with fresh CAMBH and incubated for 2 h to achieve the logarithmic growth phase. The bacterial inoculums were adjusted spectrophotometrically to reach approx. 10^5^ CFU/mL. Then the selected test compounds or vancomycin (control) were added to achieve the MIC concentration. The samples were further incubated at 37 °C with continuous agitation for 24 h. At the selected time points, the aliquots (100 µL) were taken, diluted, and plated in Tryptic Soy agar. The CFU were calculated after 24 h of incubation. The experiments were performed in triplicate.

### 3.7. Biofilm Formation Assay

The overnight culture of *Staphylococcus aureus* was adjusted with fresh media to reach OD_600 nm_ = 0.3 and was dispensed in flat bottomed 96-well microplates (100 µL). The biofilms were grown at 37 °C, 48 h and then the 2X dilutions of the compounds or vancomycin were added to each well to reach a final concentration of 0.5X MIC, MIC or 4X MIC of each compound or comparator drug. The biofilms were exposed to the test compounds for 24 h at 37 °C. After incubation, the media was gently removed, biofilms were washed 3 times using 200 µL of PBS, and then fixed for 3 h with 5% buffered paraformaldehyde. After fixation, the paraformaldehyde was removed, plates were washed 1 time with 200 µL of PBS and biofilms were stained using 0.5% crystal violet for 1 h at room temperature. After staining, plates were washed with deionized water and air-dried overnight. The biofilm absorbed crystal violet was solubilized using 30% acetic acid and quantified spectrophotometrically at OD 595 nm.

## 4. Conclusions

A series of thiazole derivatives bearing β-amino acid and aromatic moieties in the structure were synthesized, spectrally characterized, and evaluated for their antimicrobial activity using a panel of multidrug-resistant bacterial and fungal pathogens. The in vitro antimicrobial properties of the obtained compounds were evaluated against priority pathogens harboring genetically defined resistance mechanisms. The results revealed that thiazoles **2a**–**c** with the 4-halophenyl fragment possess the profound bactericidal activity against Gram-positive pathogens, which was mostly evident with *Staphylococcus aureus* (MIC of 1–2 μg/mL) harboring defined resistance mechanisms (Pan-S, MRSA, and VRSA). The compounds demonstrated low cytotoxicity using Vero cell culture model suggesting the applicability for the further pre-clinical hit to lead optimization. The compounds also exhibited promising antifungal properties against azole-resistant fungal strain of *Aspergillus fumigatus* and **2a**, **b** were found to be the best candidates. Further studies are needed to better understand the molecular basis of Gram-positive bacteria-directed bactericidal activity of thiazoles **2a**–**c** and selective activity of compounds **2a**, **b** against azole-resistant fungal pathogens as well as to validate the in vivo tolerance and bioavailability of the most active compounds.

## Figures and Tables

**Table 1:** The in silico physicochemical properties of thiazole derivatives bearing β-amino acid and aromatic moieties.

Compound	MW	No of Heavy Atoms	No of Aromatic Heavy Atoms	Fraction Csp^3^	Rotatable Bonds	H-Bond Acceptors	H-Bond Donors	Molar Refractivity	TPSA
**1**	315.39	22	12	0.12	7	2	3	92.08	110.68
**2a**	433.5	31	23	0.08	8	4	2	122.88	93.7
**2b**	449.95	31	23	0.08	8	3	2	127.93	93.7
**2c**	494.4	31	23	0.08	8	3	2	130.62	93.7
**3**	463.98	32	23	0.12	9	3	1	132.25	82.7
**4**	463.98	32	23	0.08	9	3	3	131.87	111.52
**5a**	552.09	39	29	0.06	11	3	2	162.14	97.86
**5b**	570.08	40	29	0.06	11	4	2	162.1	97.86
**5c**	586.53	40	29	0.06	11	3	2	167.15	97.86
**5d**	630.99	40	29	0.06	11	3	2	169.84	97.86
**5e**	597.09	42	29	0.06	12	5	2	170.97	143.68
**5f**	595.16	42	29	0.12	12	3	2	176.35	101.1
**5g**	566.12	40	29	0.09	11	3	2	167.11	97.86
**5h**	558.12	38	28	0.07	11	3	2	160.02	126.1
**5i**	603.11	41	28	0.07	12	5	2	168.84	171.92
**5j**	504.05	35	23	0.15	10	3	2	147.27	97.86
**5k**	518.07	36	23	0.18	11	3	2	152.08	97.86
**6**	528.07	37	28	0.14	9	3	1	153.08	91.29
**7**	542.09	38	28	0.13	10	2	2	158.85	90.43
**8**	599.17	41	29	0.06	13	2	4	173.39	141.65
**9**	581.15	40	34	0.06	9	2	2	168.15	122.1

Abbreviations: TPSA—total polar surface area, MW—molecular weight.

**Table 2:** Pharmacokinetic and drug excretion properties of thiazole derivatives bearing β-amino acid and aromatic moieties.

Comp-ound	Log Kp (cm/s)	GI Absorption	BBB Permeant	P-Gp Substrate	Inhibition of Cytochrome P450 System				
					CYP1A2	CYP2C19	CYP2C9	CYP2D6	CYP3A4
**1**	−6.56	High	No	No	No	No	Yes	No	No
**2a**	−4.85	Low	No	No	No	Yes	Yes	Yes	Yes
**2b**	−4.57	Low	No	No	No	Yes	Yes	Yes	Yes
**2c**	−4.8	Low	No	No	No	Yes	Yes	Yes	Yes
**3**	−4.43	Low	No	Yes	No	Yes	Yes	Yes	Yes
**4**	−5.37	Low	No	No	Yes	Yes	Yes	Yes	Yes
**5a**	−4.21	Low	No	No	No	Yes	No	Yes	Yes
**5b**	−4.25	Low	No	No	No	Yes	No	Yes	Yes
**5c**	−3.98	Low	No	No	No	Yes	No	Yes	Yes
**5d**	−4.21	Low	No	No	No	Yes	No	Yes	Yes
**5e**	−4.61	Low	No	No	No	Yes	No	No	Yes
**5f**	−4.39	Low	No	No	No	Yes	No	Yes	Yes
**5g**	−4.04	Low	No	No	No	Yes	No	Yes	Yes
**5h**	−4.24	Low	No	No	No	No	Yes	Yes	Yes
**5i**	−4.4	Low	No	No	No	Yes	No	No	Yes
**5j**	−4.94	Low	No	Yes	No	Yes	Yes	Yes	Yes
**5k**	−4.7	Low	No	Yes	No	Yes	Yes	Yes	Yes
**6**	−4.25	Low	No	No	No	Yes	Yes	No	Yes
**7**	−4.22	Low	No	No	No	Yes	No	Yes	Yes
**8**	−4.6	Low	No	No	No	Yes	No	No	Yes
**9**	−4.14	Low	No	No	No	No	No	No	Yes

Abbreviations: log Kp (cm/s)—skin permeability, P-gp—P glycoprotein 1; BBB—brain–blood barrier; GI—gastrointestinal; CYP1A2—cytochrome P450 1A2; CYP2C19—cytochrome P450 2C19; CYP2C9—cytochrome P450 2C9; CYP2D6—cytochrome P450 2D6; CYP3A4—cytochrome P450 3A4.

**Table 3:** The antibacterial activity expressed as minimal inhibitory concentration (MIC, μg/mL) of compounds **1**–**9** and comparator drugs (vancomycin, daptomycin, and meropenem) against panel of multidrug-resistant Gram-positive and Gram-negative bacterial pathogens harboring different resistance profiles.

Compound	Gram-Positive						Gram-Negative		
	*S. a* MRSA	*S. a* MSSA	*S. a* VRSA	*E. fec*	*E. fae* VREF	*E. raffi*	*K. pn* NDM-1	*S. m* R	*P. ae* AmpC
**1**	64<	64<	64<	64<	64<	64<	64<	64<	64<
**2a**	2	2	2	32	32	64<	64<	64<	64<
**2b**	1	1	2	64<	64<	64<	64<	64<	64<
**2c**	64	32	32	32	32	64	64<	64<	64<
**3**	64<	64<	64<	64<	64<	64<	64<	64<	64<
**4**	64<	64<	64<	64<	64<	64<	64<	64<	64<
**5a**	64<	64<	64<	64<	64<	64<	64<	64<	64<
**5b**	64<	64<	64<	64<	64<	64<	64<	64<	64<
**5c**	64<	64<	64<	64<	64<	64<	64<	64<	64<
**5d**	64<	64<	64<	64<	64<	64<	64<	64<	64<
**5e**	64<	64<	64<	64<	64<	64<	64<	64<	64<
**5f**	64<	64<	64<	64<	64<	64<	64<	64<	64<
**5g**	64<	64<	64<	64<	64<	64<	64<	64<	64<
**5h**	64<	64<	64<	64<	64<	64<	64<	64<	64<
**5i**	64<	64<	64<	64<	64<	64<	64<	64<	64<
**5j**	64<	64<	64<	64<	64<	64<	64<	64<	64<
**5k**	64<	64<	64<	64<	64<	64<	64<	64<	64<
**6**	64<	64<	64<	64<	64<	64<	64<	64<	64<
**7**	64<	64<	64<	64<	64<	64<	64<	64<	64<
**8**	64<	64<	64<	64<	64<	64<	64<	64<	64<
**9**	64<	64<	64<	64<	64<	64<	64<	64<	64<
Vancomycin	2	0.5	64<	0.5	32	32	n.a	n.a	n.a
Daptomycin	0.5<	0.5<	0.5	1	2	2	n.a	n.a	n.a
Meropenem	4	1	4	n.a	n.a	n.a	32	16	16

Abbreviations: *S. a* MRSA—MRSA *Staphylococcus aureus* USA 300 TCH-1516, *S. a* MSSA—*Staphylococcus aureus* ATCC 25923 PVL+, *S. a* VRSA *Staphylococcus aureus* USA 100 DE-11, *E. fec*—*Enterococcus faecalis* ATCC 29212, *E. fae*—*Enterococcus faecium* ATCC 51299 VREF, *E. raff*—*Enterococcus raffinosus*, *K. pn* NDM-1—*Kp. pneumoniae* NDM-1 producing strain, *S. m*—Multidrug-resistant *Stenotrophomonas maltophilia*, *P. ae* AmpC—Inducible AmpC overexpressing *Pseudomonas aeruginosa*, n.a—not applicable.

**Table 4:** The antifungal activity expressed as minimal inhibitory concentration (MIC, µg/mL) of compounds **1**–**9** and comparator drugs (voriconazole, itraconazole) against azole-resistant *Aspergillus fumigatus* with genetically defined resistance mechanisms.

Compound	*A. fumigatus* Azole Resistance Phenotype		
	L98H, TR34	F495I, L98H, S297T, TR34	Wt
**1**	64<	64<	64<
**2a**	32	32	64<
**2b**	32	32	64<
**2c**	64	64<	64
**3**	64<	64<	64<
**4**	64<	64<	64<
**5a**	64<	64<	64<
**5b**	64<	64<	64<
**5c**	64<	64<	64<
**5d**	64<	64<	64<
**5e**	64<	64<	64<
**5f**	64<	64<	64<
**5g**	64<	64<	64<
**5h**	64<	64<	64<
**5i**	64<	64<	64<
**5j**	64<	64<	64<
**5k**	64<	64<	64<
**6**	64<	64<	64<
**7**	64<	64<	64<
**8**	64<	64<	64<
**9**	64<	64<	64<
Voriconazole	8	1	0.5
Itraconazole	2	16<	1

**Table 5:** The antifungal activity expressed as minimal inhibitory concentration (MIC, µg/mL) of compounds **1**–**9** and comparator drugs (fluconazole, itraconazole) of **1**–**9** against extensively multidrug-resistant *Candida* spp.

Compound	*C. auris* 381	*C. auris* 382	*C. auris* 383	*C. auris* 384	*C. duobushaemulonii 394*	*C. krusei* *397*	*C. albicans 1214*
**1**	64<	64<	64<	64<	64<	64<	64<
**2a**	64<	64<	64<	64<	64<	64<	64<
**2b**	32	16	32	32	16	64<	64<
**2c**	64<	64<	64<	64<	64<	64<	64<
**3**	64<	64<	64<	64<	64<	64<	64<
**4**	64<	64<	64<	64<	64<	64<	64<
**5a**	64	64	64<	64<	64<	32	64<
**5b**	64<	64<	64<	64<	64<	64<	64<
**5c**	64<	64<	64<	64<	64<	64<	64<
**5d**	64<	64<	64<	64<	64<	64<	64<
**5e**	64<	64<	64<	64<	64<	64<	64<
**5f**	64<	64<	64<	64<	64<	64<	64<
**5g**	64<	64<	64<	64<	64<	64<	64<
**5h**	64<	64<	64<	64<	64<	64<	64<
**5i**	64<	64<	64<	64<	64<	64<	64<
**5j**	64<	64<	64<	64<	64<	64<	64<
**5k**	64<	64<	64<	64<	64<	64<	64<
**6**	64<	64<	64<	64<	64<	64<	64<
**7**	64<	64<	64<	64<	64<	64<	64<
**8**	64<	64<	64<	64<	64<	64<	64<
**9**	64<	64<	64<	64<	64<	64<	64<
Fluconazole	4	16	64<	64<	8	64	32
Itraconazole	0.125	1	0.5	0.5	0.5	1	1

## Data Availability

Not applicable.

## Supplementary Materials

The following are available online, Figure S1: ^1^H-NMR of compound **1**, Figure S2: ^13^C-NMR of compound **1**, Figure S3: ^1^H-NMR of compound **2a**, Figure S4: ^13^C-NMR of compound **2a**, Figure S5: ^1^H-NMR of compound **2b**, Figure S6: ^13^C-NMR of compound **2b**, Figure S7: ^1^H-NMR of compound **2c**, Figure S8: ^13^C-NMR of compound **2c**, Figure S9: ^1^H-NMR of compound **3**, Figure S10: ^13^C-NMR of compound **3**, Figure S11: ^1^H-NMR of compound **4**, Figure S12: ^13^C-NMR of compound **4**, Figure S13: ^1^H-NMR of compound **5a**, Figure S14: ^13^C-NMR of compound **5a**, Figure S15: ^1^H-NMR of compound **5b**, Figure S16: ^13^C-NMR of compound **5b**, S17: ^1^H-NMR of compound **5c**, Figure S18: ^13^C-NMR of compound **5c**, Figure S19: ^1^H-NMR of compound **5d**, Figure S20: ^13^C-NMR of compound **5d**, Figure S21: ^1^H-NMR of compound **5e**, Figure S22: ^13^C-NMR of compound **5e**, Figure S23: ^1^H-NMR of compound **5f**, Figure S24: ^13^C-NMR of compound **5f**, Figure S25: ^13^C-NMR of compound **5g**, Figure S26: ^13^C-NMR of compound **5g**, Figure S27: ^1^H-NMR of compound **5h**, Figure S28: ^13^C-NMR of compound **5h**, Figure S29: ^1^H-NMR of compound **5i**, Figure S30: ^13^C-NMR of compound **5i**, Figure S31: ^1^H-NMR of compound **5j**, Figure S32: ^13^C-NMR of compound **5j**, Figure S33: ^1^H-NMR of compound **5k**, Figure S34: ^13^C-NMR of compound **5k**, Figure S35: ^1^H-NMR of compound **6**, Figure S36: ^13^C-NMR of compound **6**, Figure S37: ^1^H-NMR of compound **7**, Figure S38: ^13^C-NMR of compound **7**, Figure S39: ^1^H-NMR of compound **8**, Figure S40: ^13^C-NMR of compound **8**, Figure S41: ^1^H-NMR of compound **9**, Figure S42: ^13^C-NMR of compound **9**. Table S1: The list of fungal strains used for the study.

## Sample Availability

Samples of the compounds are not available from the authors.

## References

[1] Kakkar A.K., Shafiq N., Singh G., Ray P., Gautam V., Agarwal R., Muralidharan J., Arora P. (2020). Antimicrobial Stewardship Programs in Resource Constrained Environments: Understanding and Addressing the Need of the Systems. Front. Public Heal..

[2] Pierce J., Apisarnthanarak A., Schellack N., Cornistein W., Al Maani A., Adnan S., Stevens M.P. (2020). Global Antimicrobial Stewardship with a Focus on Low- and Middle-Income Countries: A position statement for the international society for infectious diseases. Int. J. Infect. Dis..

[3] Shrivastava S., Sonwane S.K., Srivastava S.K. (2011). Pharmacological Significance of Synthetic Heterocycles Scaffold: A Review. Adv. Biol. Res..

[4] Koehn F.E., Carter G.T. (2005). The evolving role of natural products in drug discovery. Nat. Rev. Drug Discov..

[5] Chin Y.-W., Balunas M.J., Chai H.B., Kinghorn A.D. (2006). Drug discovery from natural sources. AAPS J..

[6] Radin N.S. (2008). Drug design: Hiding in full view. Drug Dev. Res..

[7] Ma Z., Hano Y., Nomura T., Chen Y. (2004). Novel quinazoline–quinoline alkaloids with cytotoxic and DNA topoisomerase II inhibitory activities. Bioorganic Med. Chem. Lett..

[8] Santana A.C., Filho R.C.S., Menezes J.C., Allonso D., Campos V.R. (2020). Nitrogen-Based Heterocyclic Compounds: A Promising Class of Antiviral Agents against Chikungunya Virus. Life.

[9] Sioriki E., Lordan R., Nahra F., Van Hecke K., Zabetakis I., Nolan S.P. (2018). In vitro Anti-atherogenic Properties of N-Heterocyclic Carbene Aurate(I) Compounds. ChemMedChem.

[10] Kaur R., Dahiya L., Kumar M. (2017). Fructose-1,6-bisphosphatase inhibitors: A new valid approach for management of type 2 diabetes mellitus. Eur. J. Med. Chem..

[11] Goshain O., Ahmed B. (2019). Antihypertensive activity, toxicity and molecular docking study of newly synthesized xanthon derivatives (xanthonoxypropanolamine). PLoS ONE..

[12] Chaudhari K., Surana S., Jain P., Patel H.M. (2016). Mycobacterium Tuberculosis (MTB) GyrB inhibitors: An attractive approach for developing novel drugs against TB. Eur. J. Med. Chem..

[13] Reddy D.S., Kutateladze A.G. (2019). Photoinitiated Cascade for Rapid Access to Pyrroloquinazolinone Core of Vasicinone, Luotonins, and Related Alkaloids. Org. Lett..

[14] Kucukguzel I., Kucukquzel S.G., Rollas S., Otuk-Sanis G., Ozdemir O., Bayrak I., Altug T., Stables J.P. (2004). Synthesis of some 3-(arylalkylthio)-4-alkyl/aryl-5-(4-aminophenyl)-4H-1,2,4-triazole derivatives and their anticonvulsant activity. Farmaco.

[15] Sameem B., Saeedi M., Mahdavi M., Shafiee A. (2016). A review on tacrine-based scaffolds as multi-target drugs (MTDLs) for Alzheimer’s disease. Eur. J. Med. Chem..

[16] Madhasu M., Doda S.R., Begari P.K., Dasari K.R., Thalari G., Kadari S., Yadav J.S. (2021). Concise total synthesis of antiarrhythmic drug dronedarone via a conjugate addition followed intramolecular heck cyclization. J. Heterocycl. Chem..

[17] Meng S., Tang G.-L., Pan H.-X. (2018). Enzymatic Formation of Oxygen-Containing Heterocycles in Natural Product Biosynthesis. ChemBioChem.

[18] Wang J.-L., Liu D., Zhang Z.-J., Shan S., Han X., Srinivasula S.M., Croce C.M., Alnemri E.S., Huang Z. (2000). Structure-based discovery of an organic compound that binds Bcl-2 protein and induces apoptosis of tumor cells. Proc. Natl. Acad. Sci. USA.

[19] Zhang G., Zhang Y., Yan J., Chen R., Wang S., Ma Y., Wang R. (2012). One-Pot Enantioselective Synthesis of Functionalized Pyranocoumarins and 2-Amino-4H-chromenes: Discovery of a Type of Potent Antibacterial Agent. J. Org. Chem..

[20] Gourdeau H., Leblond L., Hamelin B., Desputeau C., Dong K., Kianicka I., Custeau D., Boudreau C., Geerts L., Cai S.-X. (2004). Antivascular and antitumor evaluation of 2-amino-4-(3-bromo-4,5-dimethoxy-phenyl)-3-cyano-4H-chromenes, a novel series of anticancer agents. Mol. Cancer Ther..

[21] Salehian F., Nadri H., Jalili-Baleh L., Youseftabar-Miri L., Bukhari S.N.A., Foroumadi A., Küçükkilinç T.T., Sharifzadeh M., Khoobi M. (2020). A review: Biologically active 3,4-heterocycle-fused coumarins. Eur. J. Med. Chem..

[22] Sharma D., Kumar M., Das P. (2020). Application of cyclohexane-1,3-diones for six-membered oxygen-containing heterocycles synthesis. Bioorganic Chem..

[23] Petersen F., Amstutz R. (2008). Natural Compounds as Drugs.

[24] Pathania S., Narang R., Rawal R.K. (2019). Role of sulphur-heterocycles in medicinal chemistry: An update. Eur. J. Med. Chem..

[25] García-Valverde M., Torroba T. (2005). Sulfur-Nitrogen Heterocycles. Molecules.

[26] Qian M.C., Fan X., Mahattanatawee K. (2011). Volatile Sulfur Compounds in Food.

[27] Ninomiya M., Garud D.R., Koketsu M. (2011). Biologically significant selenium-containing heterocycles. Coord. Chem. Rev..

[28] Demkowicz S., Rachon J., Daśko M., Kozak W. (2016). Selected organophosphorus compounds with biological activity. Applications in medicine. RSC Adv..

[29] Madhu M., Doda S.R., Begari P.K., Dasari K.R., Thalari G., Kadari S., Yadav J.S. (2020). Enantioselective epoxidation by the chiral auxiliary approach: Asymmetric total synthesis of (+)-Ambrisentan. J. Heterocycl. Chem..

[30] Allen D., Wilson D., Drew R., Perfect J. (2015). Azole antifungals: 35 years of invasive fungal infection management. Expert Rev. Anti-infective Ther..

[31] Campoy S., Adrio J.L. (2017). Antifungals. Biochem. Pharmacol..

[32] Pristov K.E., Ghannoum M.A. (2019). Resistance of Candida to azoles and echinocandins worldwide. Clin. Microbiol. Infect..

[33] Petraitiene R., Petraitis V., Maung B.W., Naing E., Kavaliauskas P., Walsh T.J. (2020). Posaconazole Alone and in Combination with Caspofungin for Treatment of Experimental Exserohilum rostratum Meningoencephalitis: Developing New Strategies for Treatment of Phaeohyphomycosis of the Central Nervous System. J. Fungi..

[34] Girmenia C. (2009). New generation azole antifungals in clinical investigation. Expert Opin. Investig. Drugs.

[35] Denning D.W., Cadranel J., Beigelman-Aubry C., Ader F., Chakrabarti A., Blot S., Ullmann A.J., Dimopoulos G., Lange C., Dimopoulos, Christoph Lange on behalf of the European Society for Clinical Microbiology and Infectious Diseases and European Respiratory Society (2016). Chronic pulmonary aspergillosis: Rationale and clinical guidelines for diagnosis and management. Eur. Respir. J..

[36] Schweer K.E., Bangard C., Hekmat K., Cornely O.A. (2013). Chronic pulmonary aspergillosis. Mycoses.

[37] Vermeulen E., Maertens J., De Bel A., Nulens E., Boelens J., Surmont I., Mertens A., Boel A., Lagrou K. (2015). Nationwide Surveillance of Azole Resistance in Aspergillus Diseases. Antimicrob. Agents Chemother..

[38] Valipour M., Naderi N., Heidarli E., Shaki F., Motafeghi F., Amiri F.T., Emami S., Irannejad H. (2021). Design, synthesis and biological evaluation of naphthalene-derived (arylalkyl)azoles containing heterocyclic linkers as new anticonvulsants: A comprehensive in silico, in vitro, and in vivo study. Eur. J. Pharm. Sci..

[39] Sui Y.-F., Li D., Wang J., Bheemanaboina R.R.Y., Ansari M.F., Gan L.-L., Zhou C.-H. (2020). Design and biological evaluation of a novel type of potential multi-targeting antimicrobial sulfanilamide hybrids in combination of pyrimidine and azoles. Bioorganic Med. Chem. Lett..

[40] Lv P.-C., Sun J., Luo Y., Yang Y., Zhu H.-L. (2010). Design, synthesis, and structure–activity relationships of pyrazole derivatives as potential FabH inhibitors. Bioorganic Med. Chem. Lett..

[41] Foroumadi A., Mirzaei M., Shafiee A. (2001). Antituberculosis agents, I: Synthesis and antituberculosis activity of 2-aryl-1,3,4-thiadiazole derivatives. Die Pharm..

[42] Zhan P., Li D., Chen X., Liu X., De Clercq E. (2011). Functional Roles of Azoles Motif in Anti-HIV Agents. Curr. Med. Chem..

[43] Mermer A., Bayrak H., Şirin Y., Emirik M., Demirbaş N. (2019). Synthesis of novel Azol-β-lactam derivatives starting from phenyl piperazine and investigation of their antiurease activity and antioxidant capacity comparing with their molecular docking studies. J. Mol. Struct..

[44] Palaska E., Şahin G., Kelicen P., Durlu N., Altinok G. (2002). Synthesis and anti-inflammatory activity of 1-acylthiosemicarbazides, 1,3,4-oxadiazoles, 1,3,4-thiadiazoles and 1,2,4-triazole-3-thiones. IL Farm..

[45] Tiperciuc B., Pârvu A., Tamaian R., Nastasă C.M., Ionuţ I., Oniga O. (2013). New anti-inflammatory thiazolyl-carbonyl-thiosemicarbazides and thiazolyl-azoles with antioxidant properties as potential iNOS inhibitors. Arch. Pharmacal Res..

[46] Khodairy A., Ahmed E.A., Ismael M., Mohamed K.M., Thabet S.A. (2019). Design and Synthesis of Some New Analgesic Azole Derivatives Containing Tramadol Moiety. J. Heterocycl. Chem..

[47] Hou Y., Shang C., Meng T., Lou W. (2021). Anticancer potential of cardiac glycosides and steroid-azole hybrids. Steroids.

[48] Bhaumik P.K., Ghosh K., Chattopadhyay S. (2021). Synthetic strategies, crystal structures and biological activities of metal complexes with the members of azole family: A review. Polyhedron.

[49] Hurtado J., Ibarra L., Yepes D., García-Huertas P., Macías M.A., Triana-Chavez O., Nagles E., Suescun L., Muñoz-Castro A. (2017). Synthesis, crystal structure, catalytic and anti-Trypanosoma cruzi activity of a new chromium(III) complex containing bis(3,5-dimethylpyrazol-1-yl)methane. J. Mol. Struct..

[50] Mohamed G.G., Zayed M., Abdallah S. (2010). Metal complexes of a novel Schiff base derived from sulphametrole and varelaldehyde. Synthesis, spectral, thermal characterization and biological activity. J. Mol. Struct..

[51] Bello-Vieda N.J., Pastrana H.F., Garavito M.F., Ávila A.G., Celis A.M., Muñoz-Castro A., Restrepo S., Hurtado J.J. (2018). Antibacterial Activities of Azole Complexes Combined with Silver Nanoparticles. Molecules.

[52] Castillo K.F., Bello N., Nuñez-Dallos N., Pastrana H., Celis A.M., Restrepo S., Hurtado J., Ávila A.G. (2016). Metal Complex Derivatives of Azole: A Study on Their Synthesis, Characterization, and Antibacterial and Antifungal Activities. J. Braz. Chem. Soc..

[53] Gagini T., Colina-Vegas L., Villarreal W., Borba-Santos L.P., Pereira C.D.S., Batista A.A., Fleury M.K., de Souza W., Rozental S., Costa L.A.S. (2018). Metal–azole fungistatic drug complexes as anti-Sporothrix spp. agents. New J. Chem..

[54] Sharma M., Prasher P. (2019). An epigrammatic status of the ‘azole’-based antimalarial drugs. RSC Med. Chem..

[55] Elsayed S.A., Harrypersad S., Sahyon H.A., Abu El-Magd M., Walsby C.J. (2020). Ruthenium(II)/(III) DMSO-Based Complexes of 2-Aminophenyl Benzimidazole with In Vitro and In Vivo Anticancer Activity. Molecules.

[56] Sapijanskaitė-Banevič B., Palskys V., Vaickelionienė R., Šiugždaitė J., Kavaliauskas P., Grybaitė B., Mickevičius V. (2021). Synthesis and Antibacterial Activity of New Azole, Diazole and Triazole Derivatives Based on *p*-Aminobenzoic Acid. Molecules.

[57] Grybaitė B., Vaickelionienė R., Stasevych M., Komarovska-Porokhnyavets O., Kantminienė K., Novikov V., Mickevičius V. (2019). Synthesis and Antimicrobial Activity of Novel Thiazoles with Reactive Functional Groups. Chem. Select..

[58] Parašotas I., Anusevičius K., Vaickelionienė R., Jonuškienė I., Stasevych M., Zvarych V., Komarovska-Porokhnyavets O., Novikov V., Belyakov S., Mickevičius V. (2018). Synthesis and evaluation of the antibacterial, antioxidant activities of novel func-tionalized thiazole and bis(thiazol-5-yl)methane derivatives. Arkivoc.

[59] Tumosienė I., Peleckis A., Jonuškienė I., Vaickelionienė R., Kantminienė K., Šiugždaitė J., Beresnevičius Z.J., Mickevičius V. (2018). Synthesis of novel 1,2- and 2-substituted benzimidazoles with high antibacterial and antioxidant activity. Mon. Chem. Chem..

[60] Balandis B., Ivanauskaitė G., Smirnovienė J., Kantminienė K., Matulis D., Mickevičius V., Zubrienė A. (2020). Synthesis and structure–affinity relationship of chlorinated pyrrolidinone-bearing benzenesulfonamides as human carbonic anhydrase inhibitors. Bioorganic Chem..

[61] Beresnevicius Z.J., Mickevicius V., Rutkauskas K., Kantminiene K. (2003). Synthesis of hexahydropyrimidine derivatives and their polymer stabilizing properties. Pol. J. Chem. Technol..

[62] Stasevych M., Lubenets V., Musyanovych R., Novikov V., Mickevicius V., Beresnevicius Z.I., Rutkauskas K. (2011). Novel thi-azolone derivatives of N-aryl-β-alanines. Chem. Heterocycl. Compd..

[63] Kantminienė K., Parašotas I., Urbonavičiūtė E., Anusevičius K., Tumosienė I., Jonuškienė I., Vaickelionienė R., Mickevičius V. (2017). Synthesis and Biological Evaluation of Novel Di- and Trisubstituted Thiazole Derivatives. Heterocycles.

[64] Tumosienė I., Beresnevičius Z.J., Kantminienė K., Mikulskienė G. (2008). Synthesis of 3-{[2-(N1-alkylidenehydrazinocarbonyl)ethyl](4-alkoxyphenyl)amino}propanohydrazide derivatives and analysis of their isomer composition. Chemija.

[65] Daina A., Zoete V. (2019). Application of the SwissDrugDesign Online Resources in Virtual Screening. Int. J. Mol. Sci..

[66] Kavaliauskas P., Grybaite B., Mickevicius V., Petraitiene R., Grigaleviciute R., Planciuniene R., Gialanella P., Pockevicius A., Petraitis V. (2020). Synthesis, ADMET Properties, and In Vitro Antimicrobial and Antibiofilm Activity of 5-Nitro-2-thiophenecarbaldehyde N-((E)-(5-Nitrothienyl)methylidene)hydrazone (KTU-286) against *Staphylococcus aureus* with Defined Resistance Mechanisms. Antibiotics.

[67] Lipinski C.A. (2004). Lead- and drug-like compounds: The rule-of-five revolution. Drug Discov. Today Technol..

[68] De Sá N.P., Pôssa A.P., Perez P., Ferreira J.M.S., Fonseca N.C., Lino C.I., Cruz L.B., De Oliveira R.B., Rosa C.A., Borelli B.M. (2019). Antifungal Activity Directed Toward the Cell Wall by 2-Cyclohexylidenhydrazo- 4-Phenyl-Thiazole Against Candida albicans. Infect. Disord. Drug Targets..

[69] Turel O. (2011). Newer antifungal agents. Expert Rev. Anti-Infect. Ther..

[70] Pricopie A.-I., Focșan M., Ionuț I., Marc G., Vlase L., Gaina I.L., Vodnar D.C., Simon E., Barta G., Pîrnău A. (2020). Novel 2,4-Disubstituted-1,3-Thiazole Derivatives: Synthesis, Anti-Candida Activity Evaluation and Interaction with Bovine Serum Albumine. Molecules.

[71] Jeanvoine A., Rocchi S., Bellanger A., Reboux G., Millon L. (2019). Azole-resistant Aspergillus fumigatus: A global phenomenon originating in the environment?. Med. Mal. Infect..

[72] Pérez-Cantero A., López-Fernández L., Guarro-Artigas J., Capilla J. (2019). Azole resistance mechanisms in Aspergillus: Update and recent advances. Int. J. Antimicrob. Agents..

[73] Spivak E.S., Hanson K.E. (2018). Candida auris: An Emerging Fungal Pathogen. J. Clin. Microbiol..

[74] Rhodes J., Fisher M.C. (2019). Global epidemiology of emerging Candida auris. Curr. Opin. Microbiol..

[75] CLSI (2019). M100 Performance Standards for Antimicrobial Susceptibility Testing.

