# Supplementary material for: Synthesis of Novel Thiazole Derivatives Bearing β-Amino Acid and Aromatic Moieties as Promising Scaffolds for the Development of New Antibacterial and Antifungal Candidates Targeting Multidrug-Resistant Pathogens
Source: Molecules. 2021 Dec 23;27(1):74. doi: 10.3390/molecules27010074 (PMC8746625; doi:10.3390/molecules27010074)
Supplement: Supplementary file 1 [file molecules-27-00074-s001.zip › molecules-1497889-supplementary.pdf]

Supplementary Materials

Synthesis of Novel Thiazole Derivatives Bearing β-Amino Acid and Aromatic Moieties as Promising Scaffolds for the Development of New Antibacterial and Antifungal Candidates Targeting Multidrug-Resistant Pathogens

Dovilė Malūkaitė [1], Birutė Grybaitė [1], Rita Vaickelionienė [1,*], Giedrius Vaickelionis [1], Birutė Sapijanskaitė-Banevič [1], Povilas Kavaliauskas [1,2,3,4,5] and Vytautas Mickevičius [1]

[1] Department of Organic Chemistry, Kaunas University of Technology, Radvilėnų Rd. 19, LT-50254 Kaunas, Lithuania; dovile.malukaite@ktu.edu (D.M.); birute.grybaite@ktu.lt (B.G.); giedrius.vaickelionis@ktu.lt (G.V.); birute.sapijanskaite@ktu.lt (B.S.-B.); pok4001@med.cornell.edu (P.K); vytautas.mickevicius@ktu.lt (V.M.)
[2] Weill Cornell Medicine of Cornell University, 527 East 68th Street, New York, NY 10065, USA
[3] Institute for Genome Sciences, School of Medicine, University of Maryland, 655 W. Baltimore Street, Baltimore, MD 21201, USA
[4] Biological Research Center, Veterinary Academy, Lithuanian University of Health Sciences, Tilžės Str. 18, LT-47181 Kaunas, Lithuania.
[5] Institute of Infectious Diseases and Pathogenic Microbiology, Birštono str. 38A, Prienai, LT-59116, Lithuania.
* Correspondence: rita.vaickelioniene@ktu.lt; Tel.: +370 600 16958.

- **NMR Spectra (1-9, all in DMSO-d_6, *Figures S1-S42*)**
- **The list of fungal strains used for the study**

3-(1-(4-Phenylaminophenyl)thioureido)propanoic acid **(1)**

Figure S1. ^{1}H NMR of compound **1.**

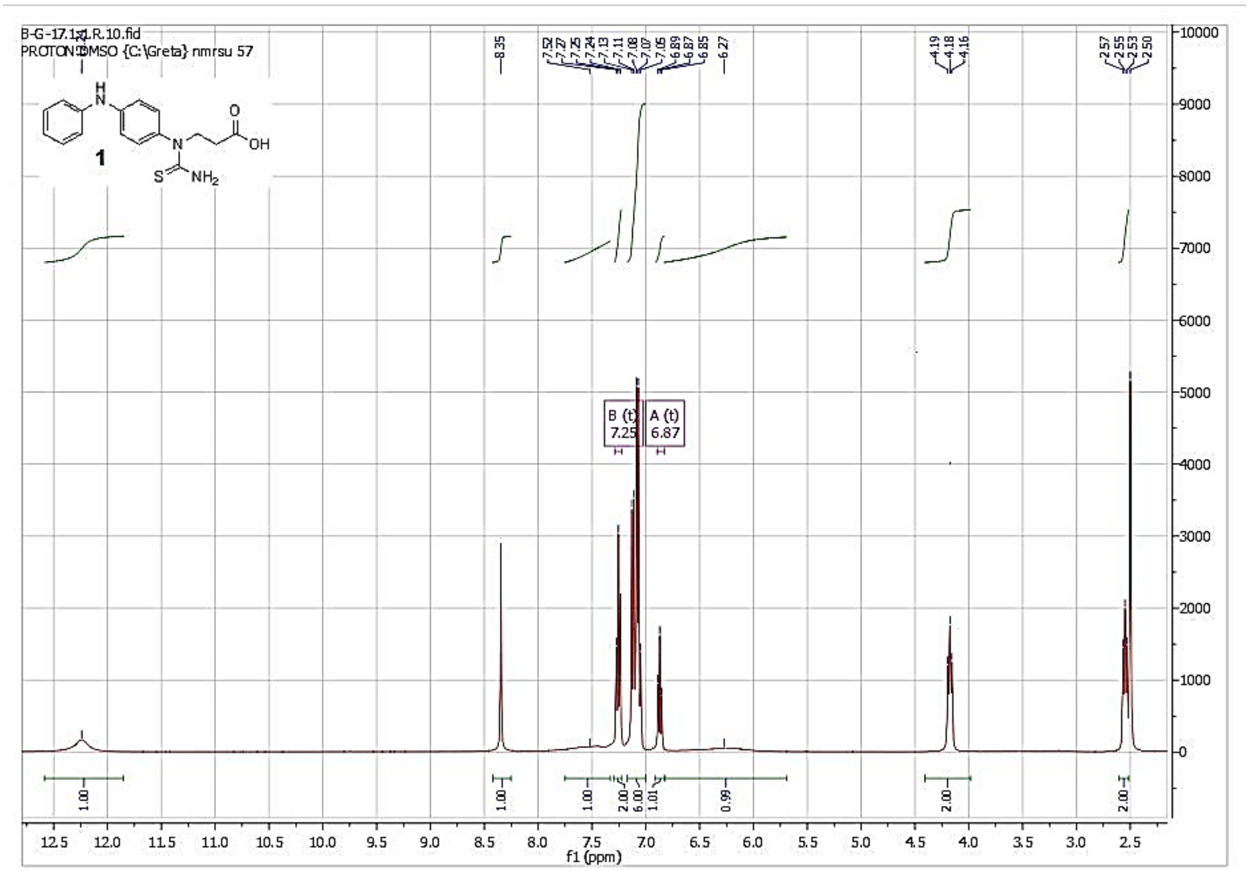

Figure S2: ^{13}C NMR of compound **1**

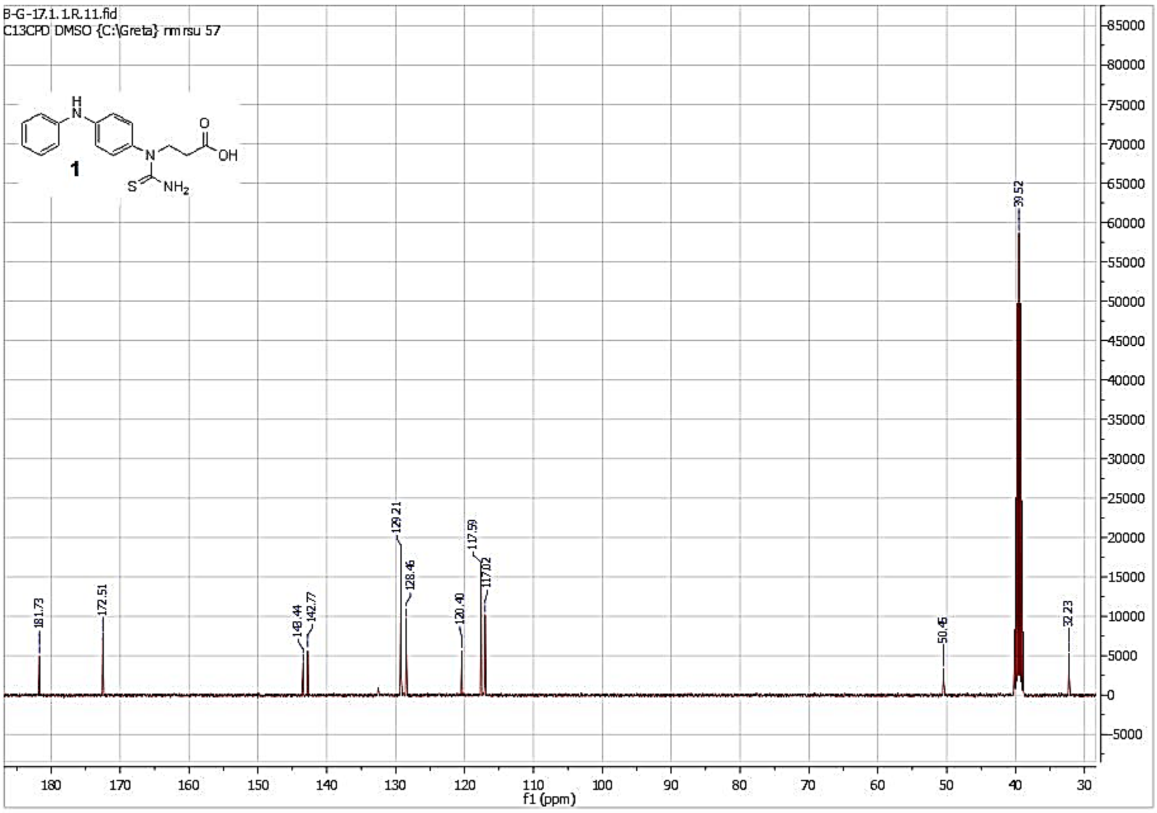

Synthesis of thiazoles 2-5

3-((4-(4-Fluorophenyl)thiazol-2-yl)(4-(phenylamino)phenyl)amino)propanoic acid **(2a):**

Figure S3: ^{1}H NMR of compound **2a**

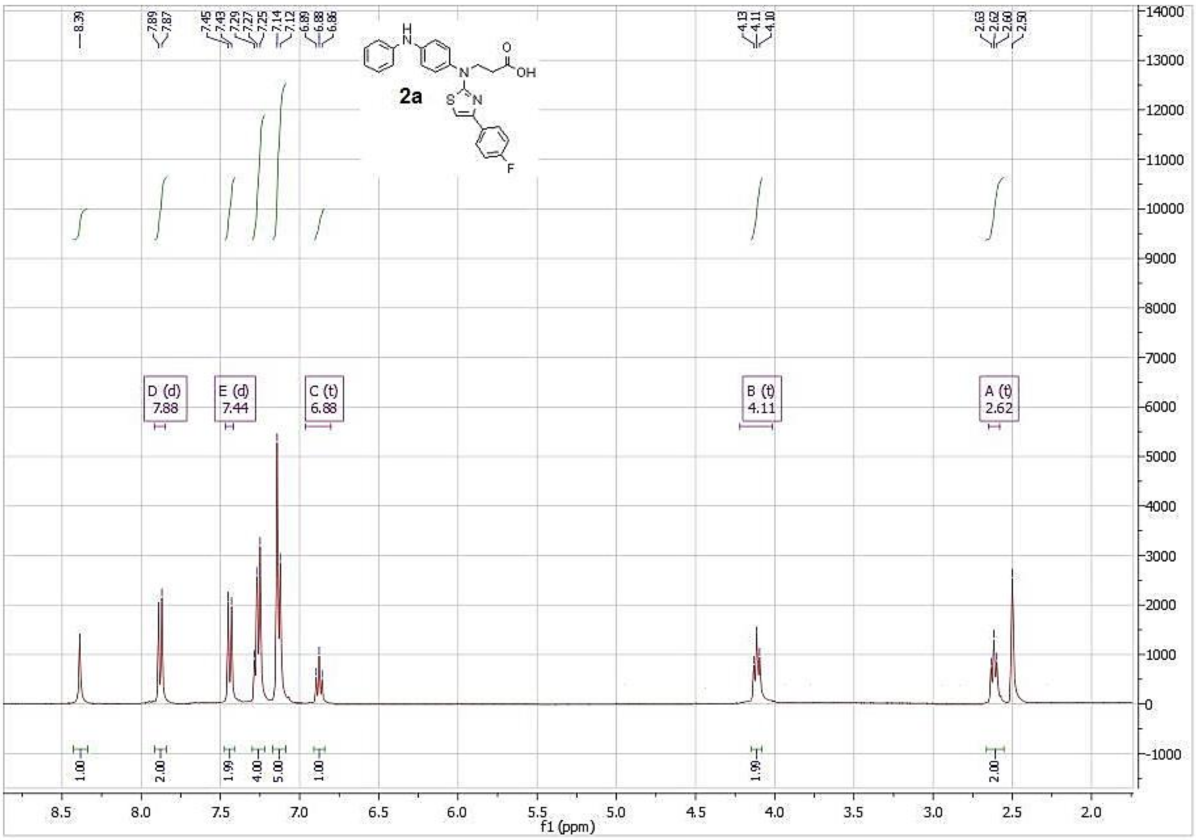

Figure S4: ^{13}C NMR of compound **2a**

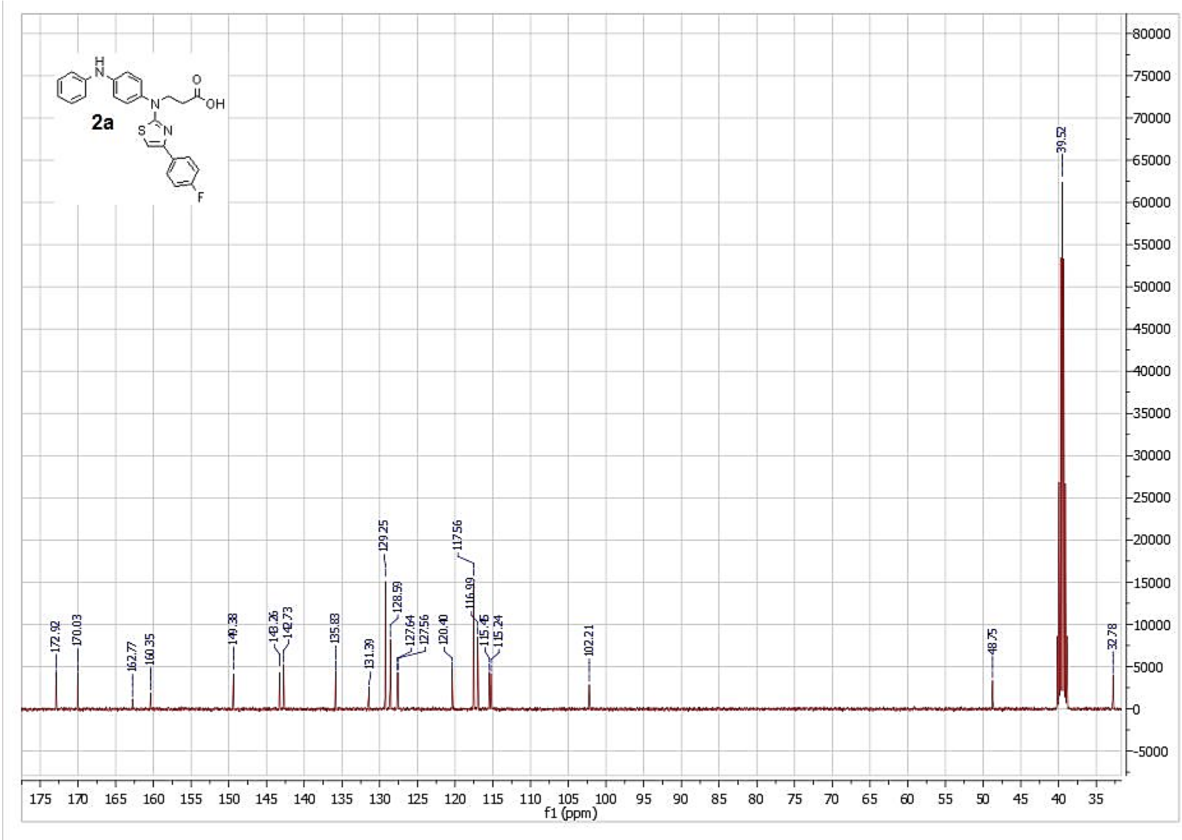

3-((4-(4-Chlorophenyl)thiazol-2-yl)(4-(phenylamino)phenyl)amino)propanoic acid **(2b):**
Figure S5: ^{1}H NMR of compound **2b**

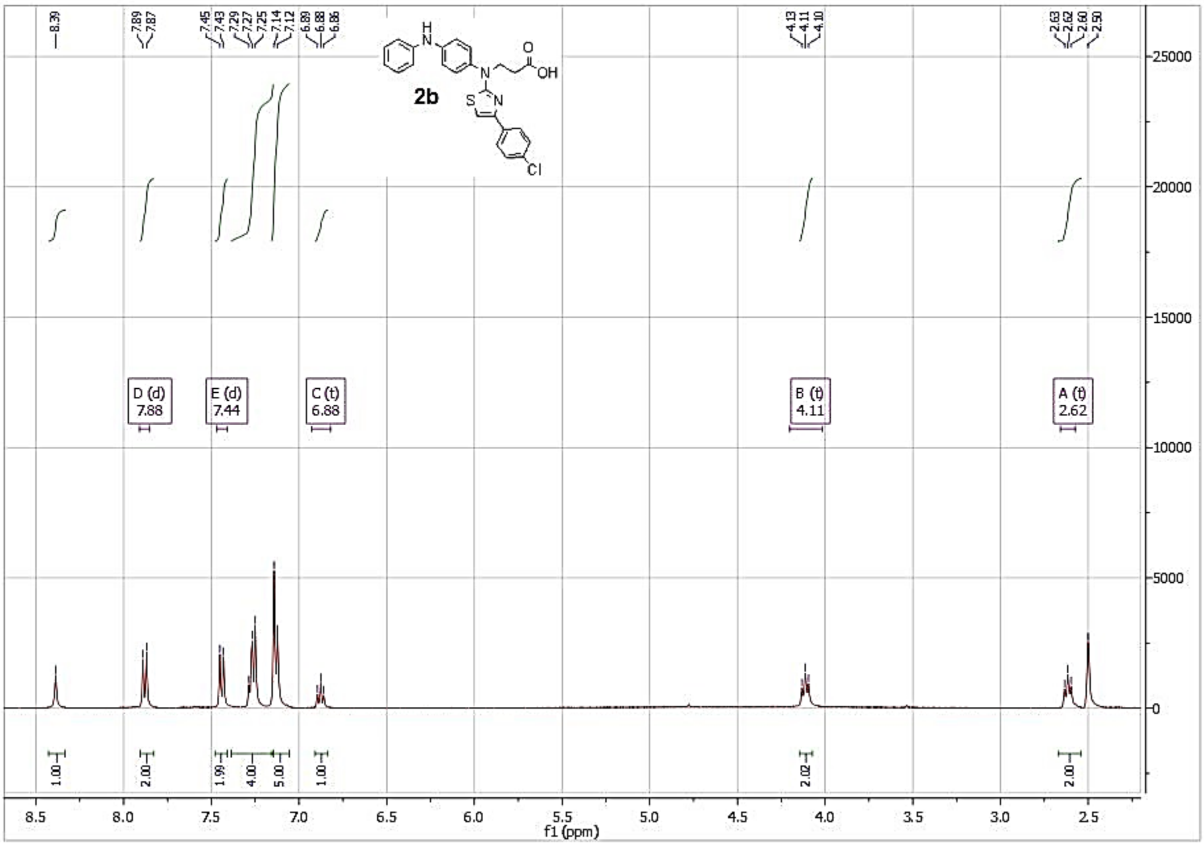

Figure S6: ^{13}C NMR of compound **2b**

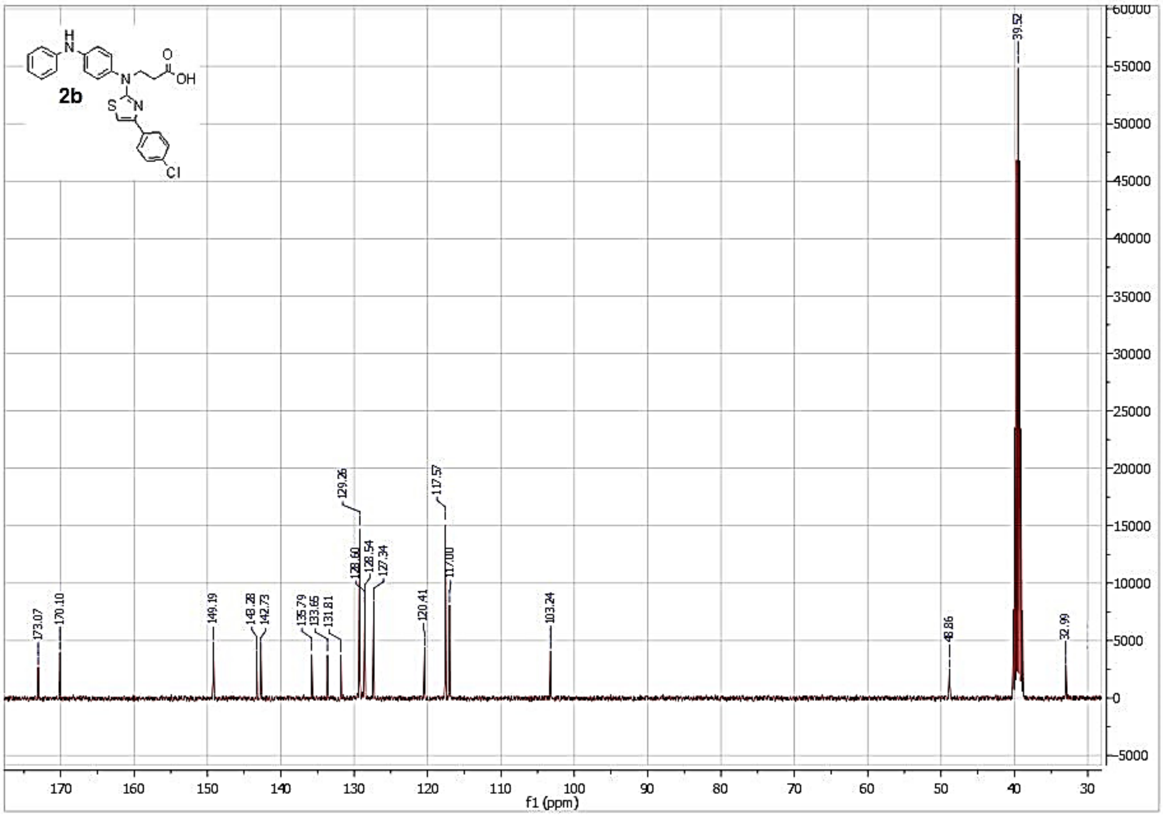

3-((4-(4-Bromophenyl)thiazol-2-yl)(4-(phenylamino)phenyl)amino)propanoic acid **(2c):**

Figure S7: ^{1}H NMR of compound **2c**

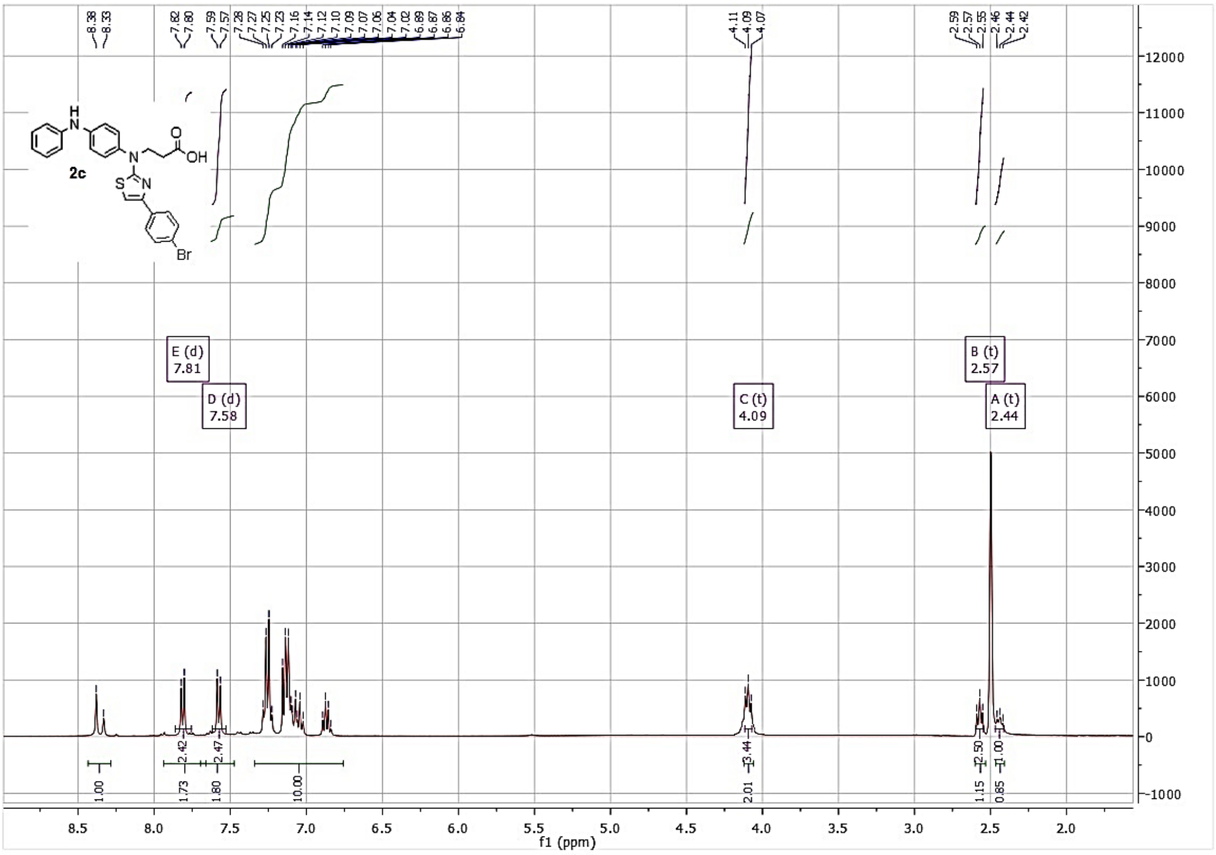

Figure S8: ^{13}C NMR of compound **2c**

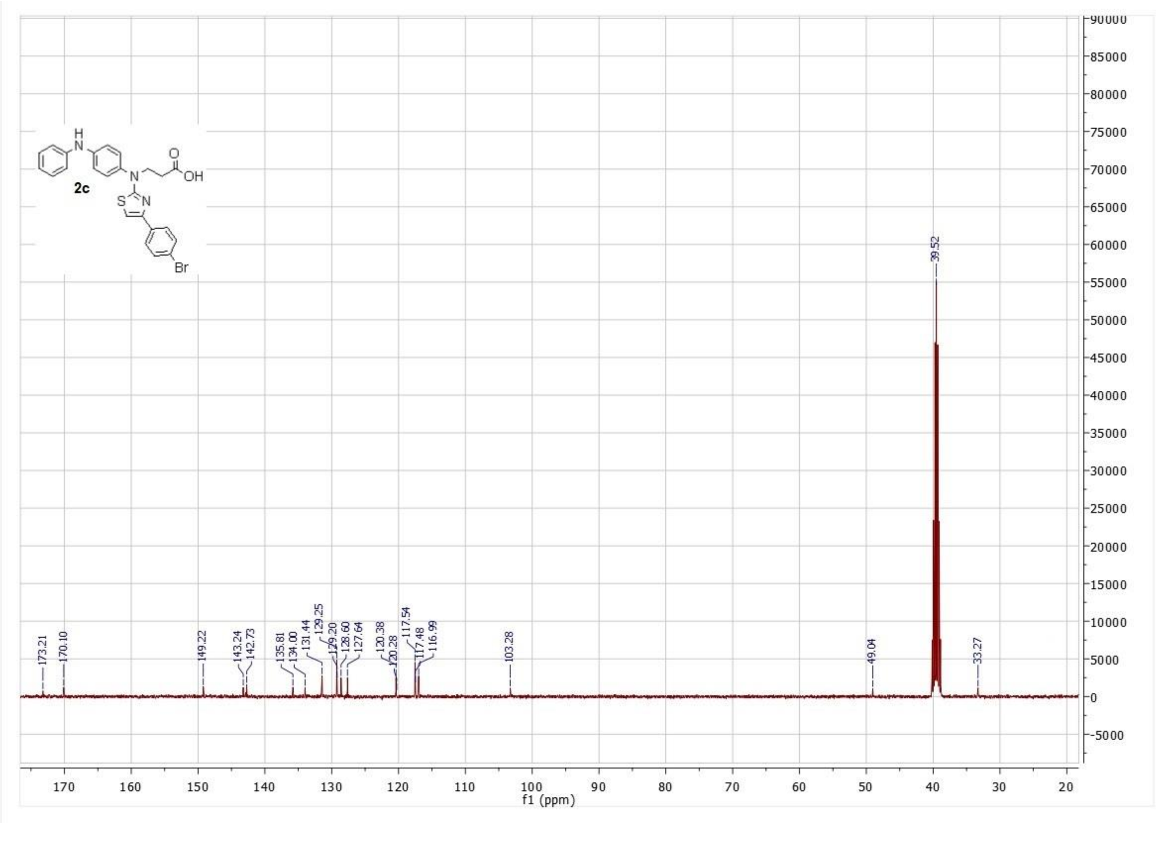

Methyl 3-((4-(4-chlorophenyl)thiazol-2-yl)(4-(phenylamino)phenyl)amino)propanoate **(3)**
Figure S9: ^{1}H NMR of compound **3**

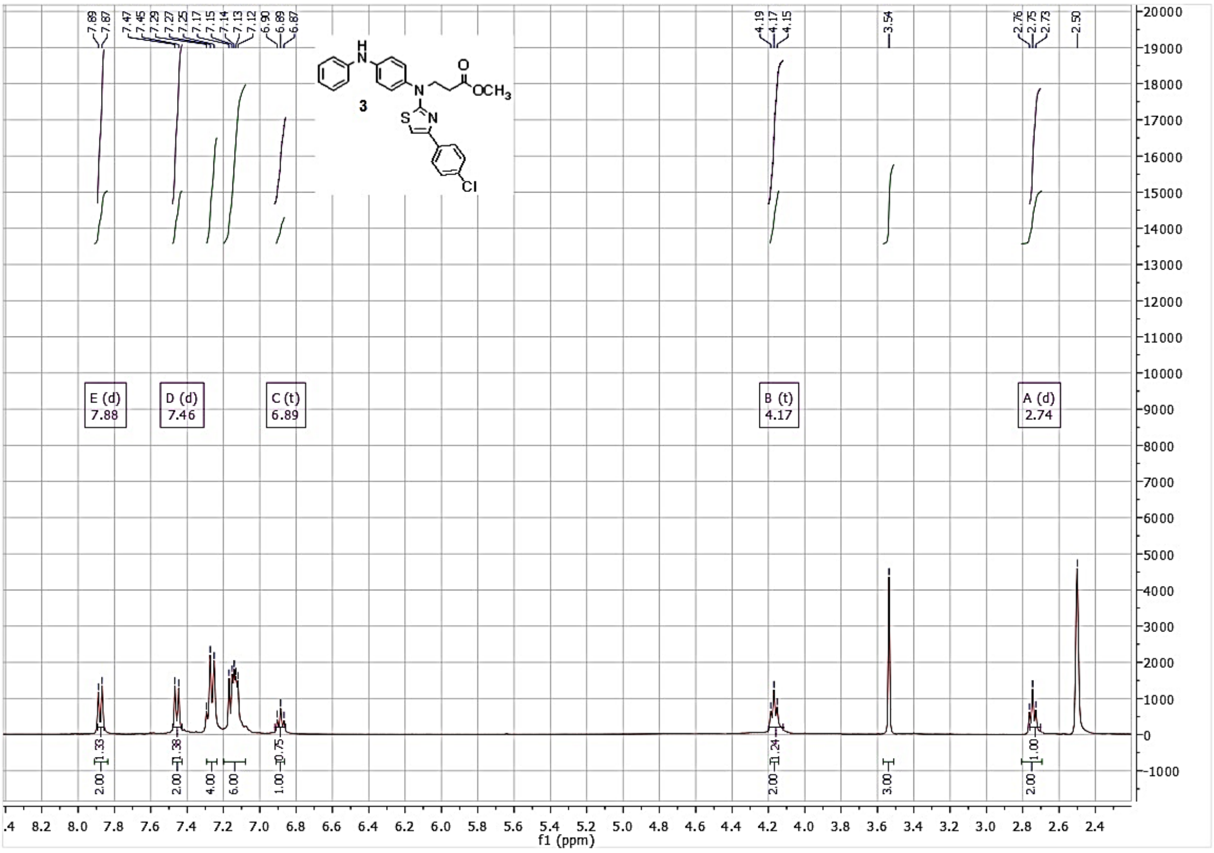

Figure S10: ^{13}C NMR of compound **3**

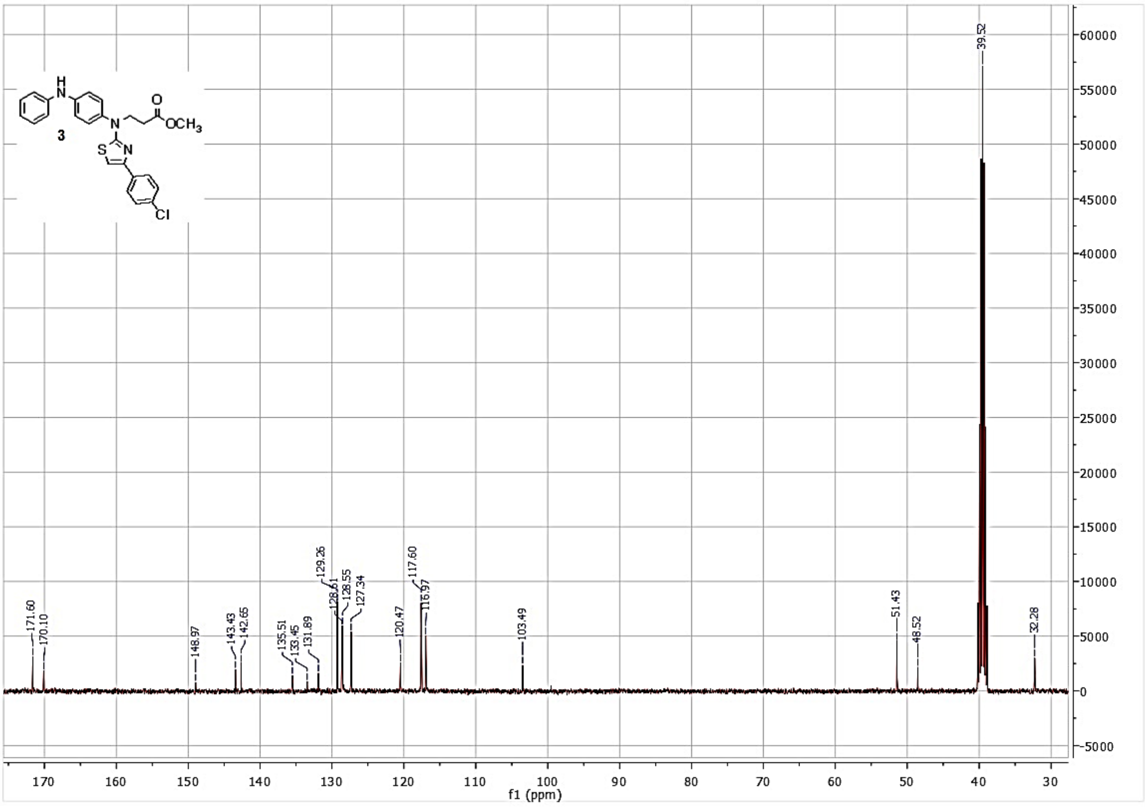

3-((4-(4-Chlorophenyl)thiazol-2-yl)(4-(phenylamino)phenyl)amino)propanehydrazide **(4)**

Figure S11: ^{1}H NMR of compound **4**

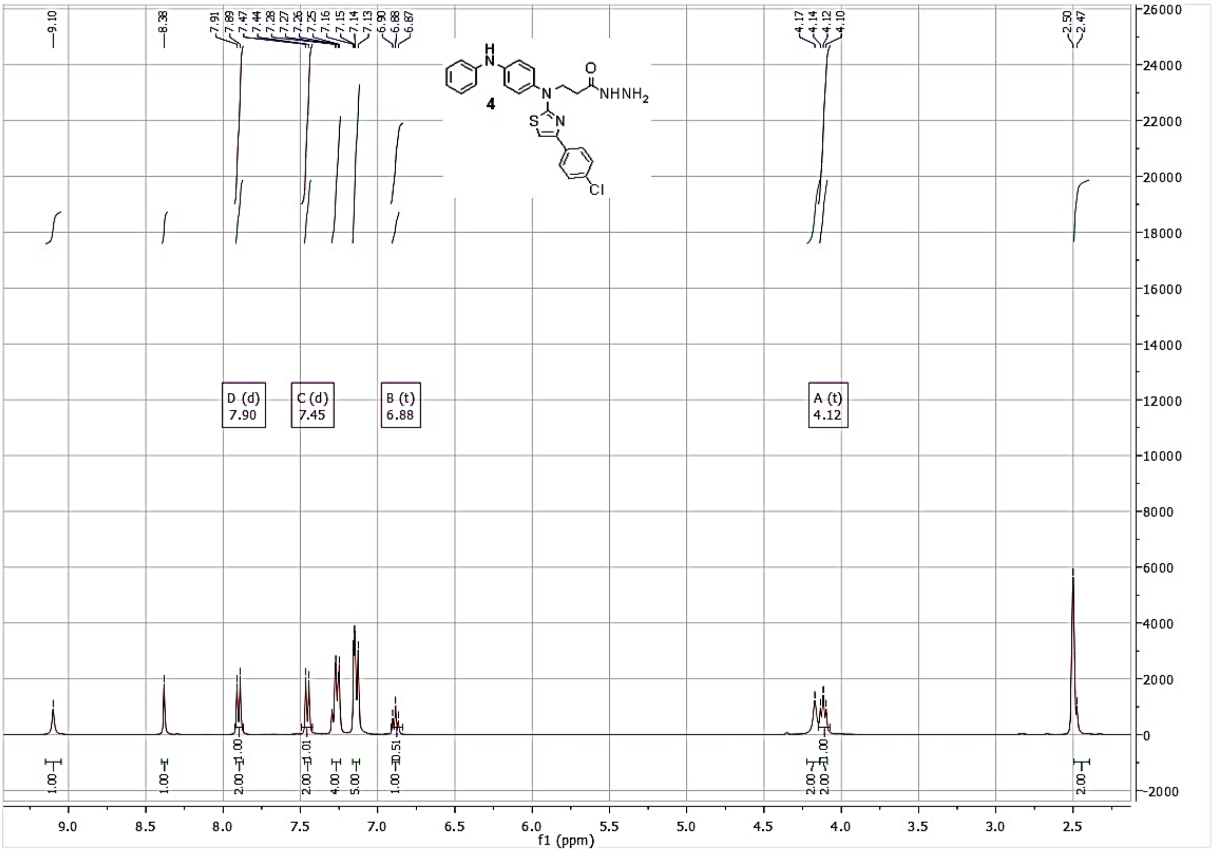

Figure S12: ^{13}C NMR of compound **4**

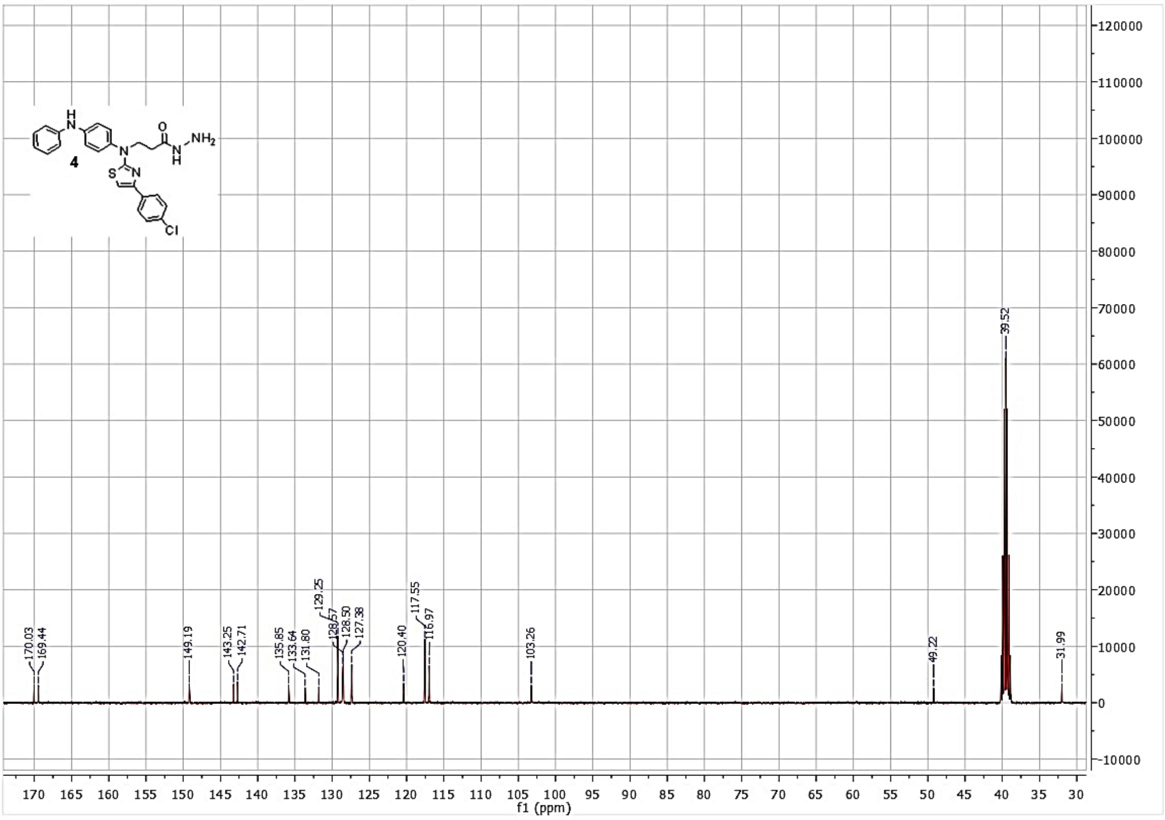

N-benzylidene-3-((4-(4-chlorophenyl)thiazol-2-yl)(4-(phenylamino)phenyl)amino)propanehydrazide **(5a)**

Figure S13: ^{1}H NMR of compound **5a**

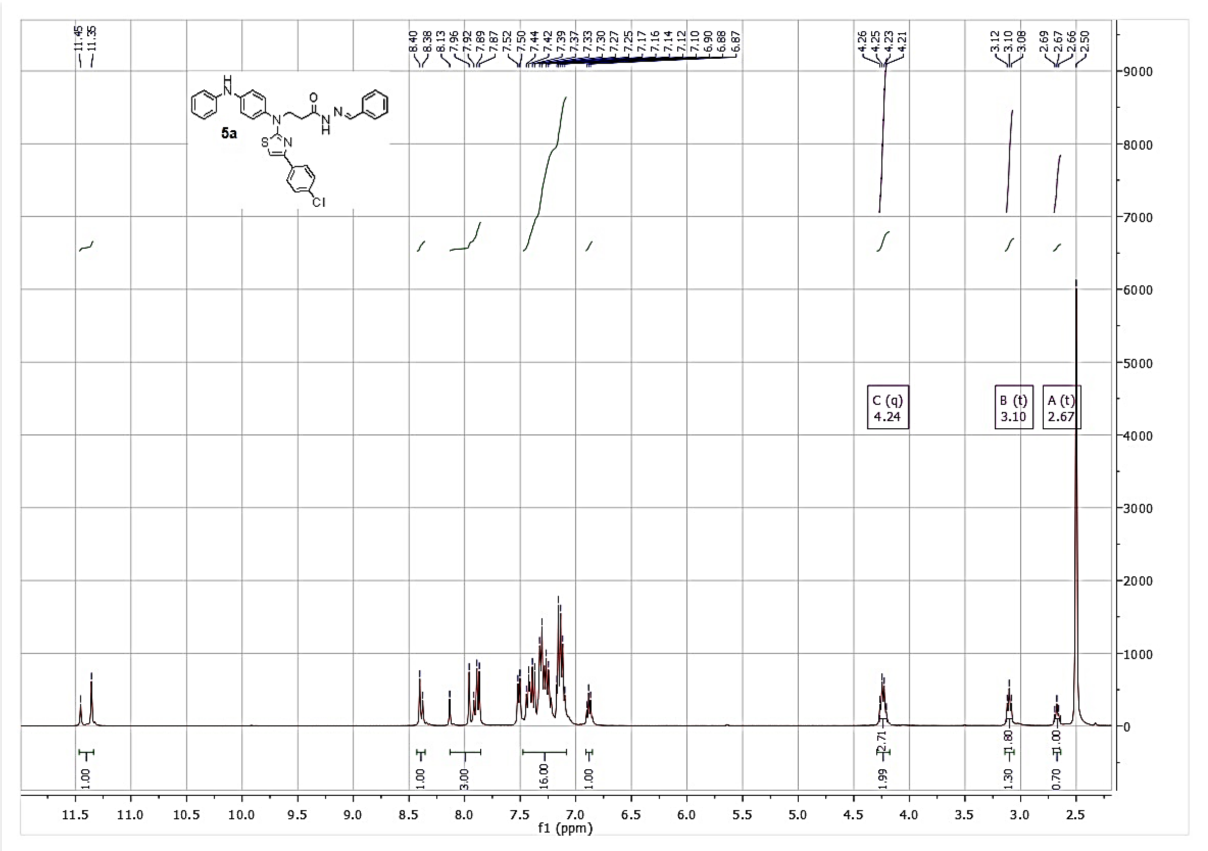

Figure S14: ^{13}C NMR of compound **5a**

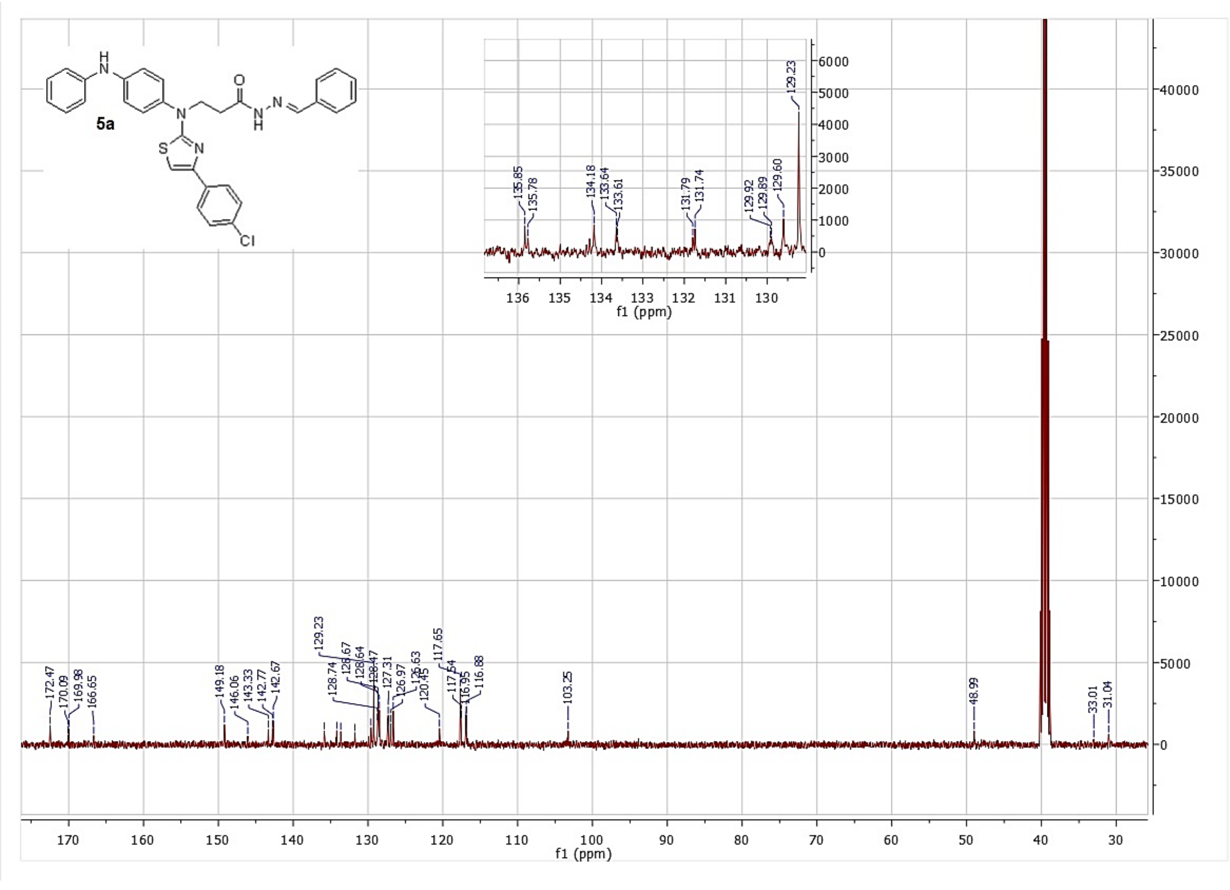

3-((4-(4-Chlorophenyl)thiazol-2-yl)(4-(phenylamino)phenyl)amino)-N'-(4-fluorobenzylidene)propanehydrazide **(5b)**

Figure S15: ^{1}H NMR of compound **5b**

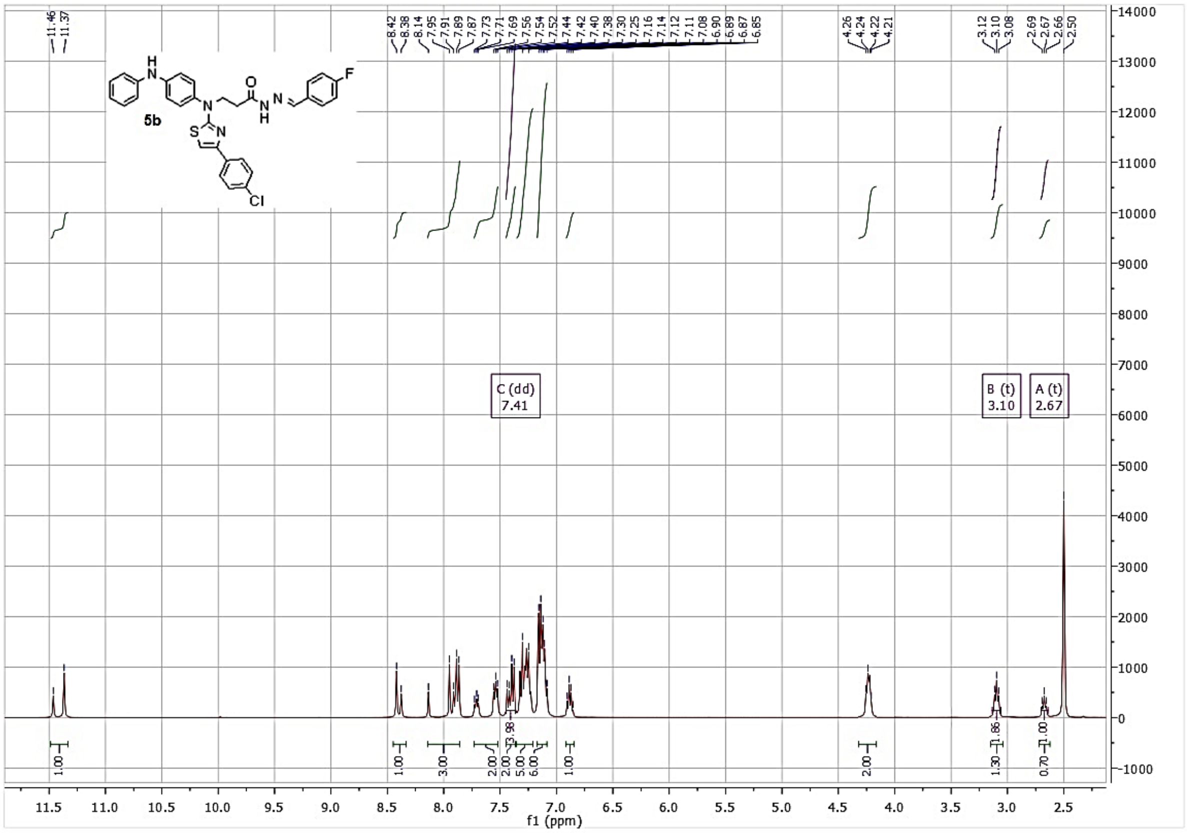

Figure S16: ^{13}C NMR of compound **5b**

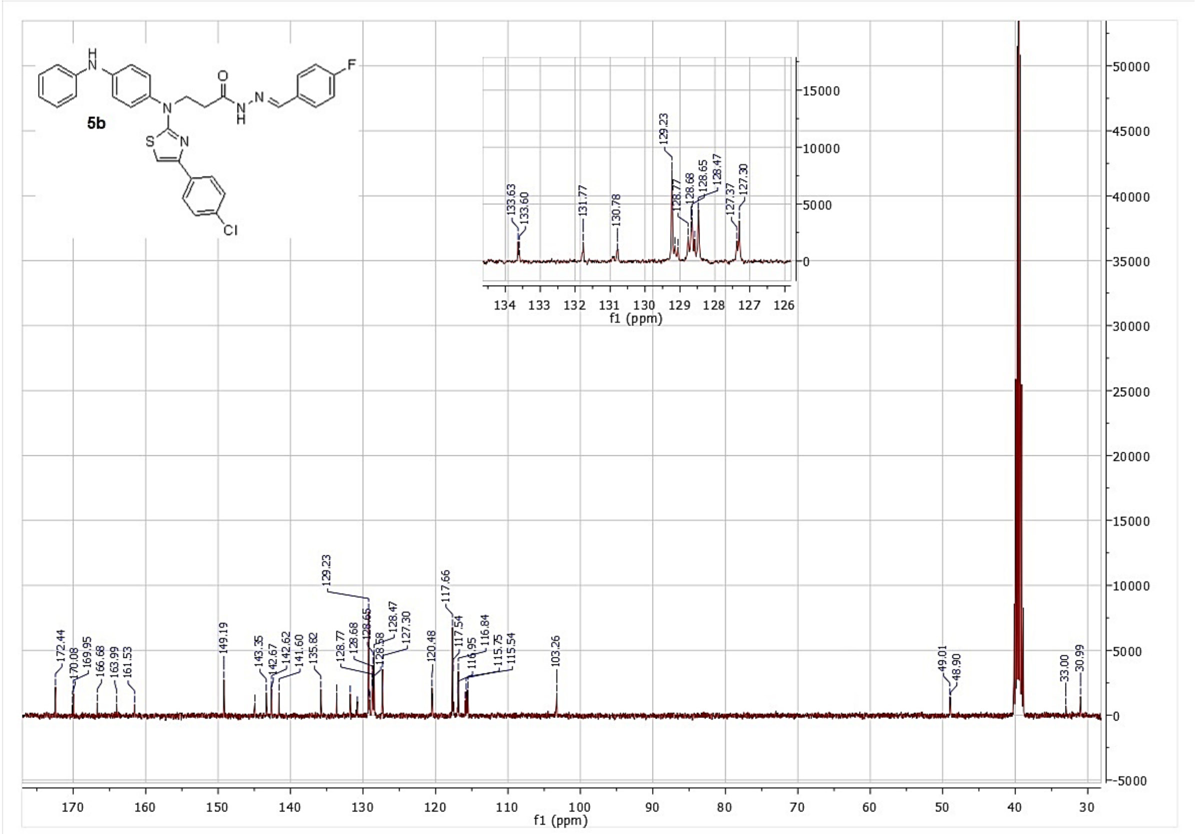

3-((4-(4-Chlorophenyl)thiazol-2-yl)(4-(phenylamino)phenyl)amino)-N'-(4-chlorobenzylidene)propanehydrazide **(5c)**

Figure S17: ^{1}H NMR of compound **5c**

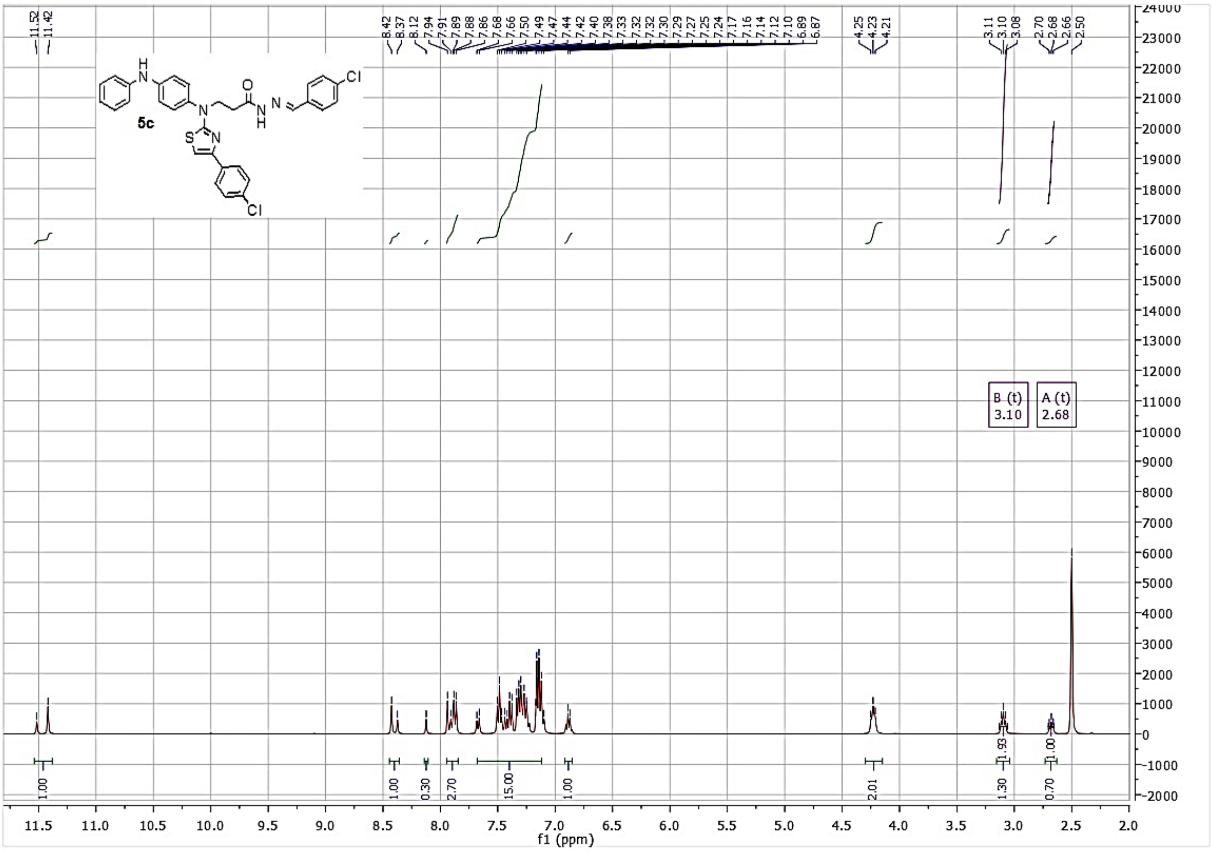

Figure S18: ^{13}C NMR of compound **5c**

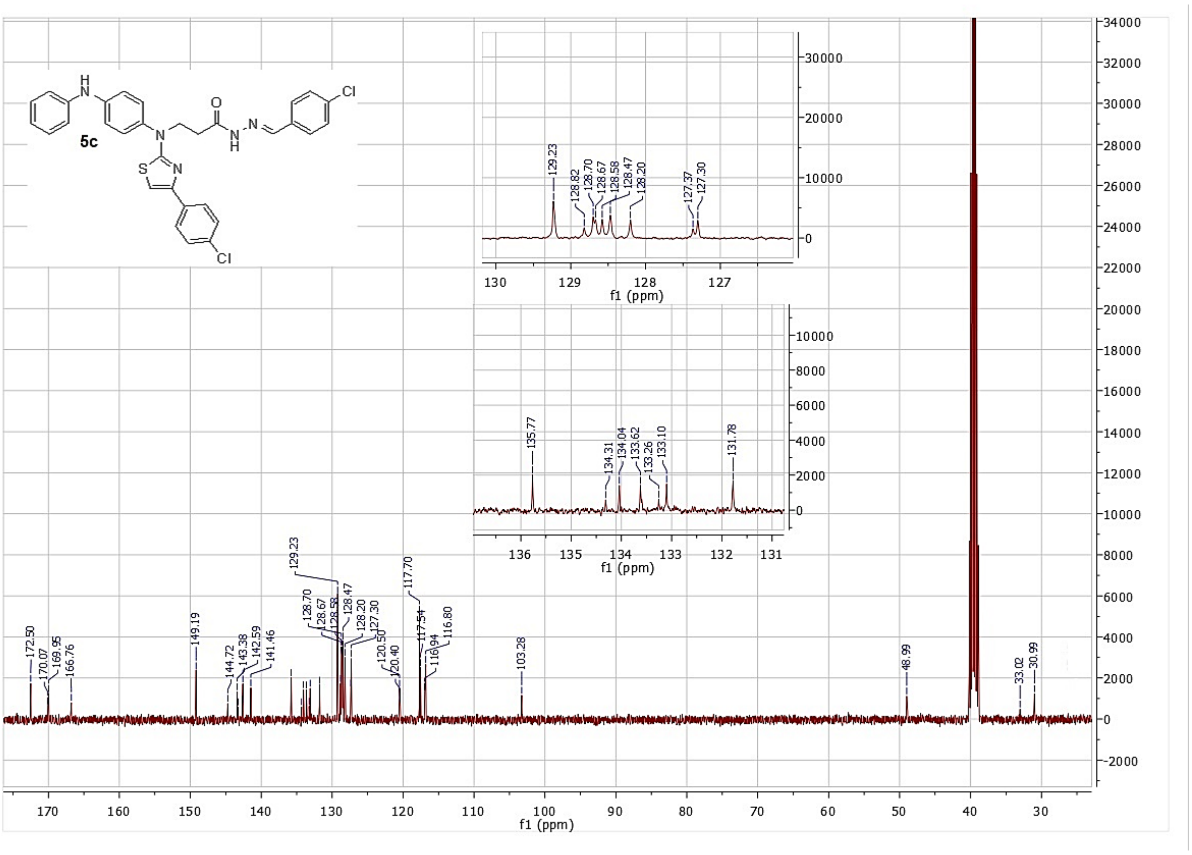

N'-(4-bromobenzylidene)-3-((4-(4-chlorophenyl)thiazol-2-yl)(4-(phenylamino)phenyl)amino)propanehydrazide **(5d)**

Figure S19: ^{1}H NMR of compound **5d**

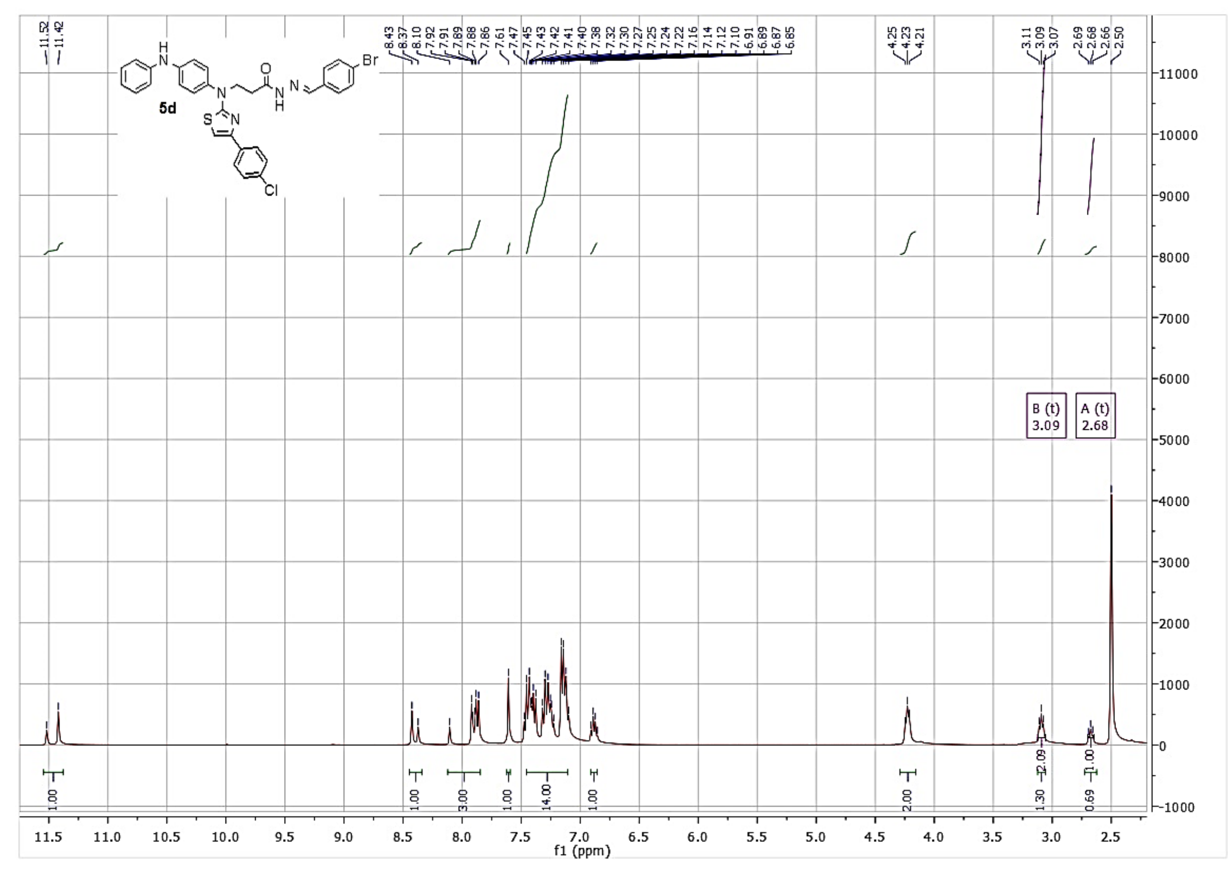

Figure S20: ^{13}C NMR of compound **5d**

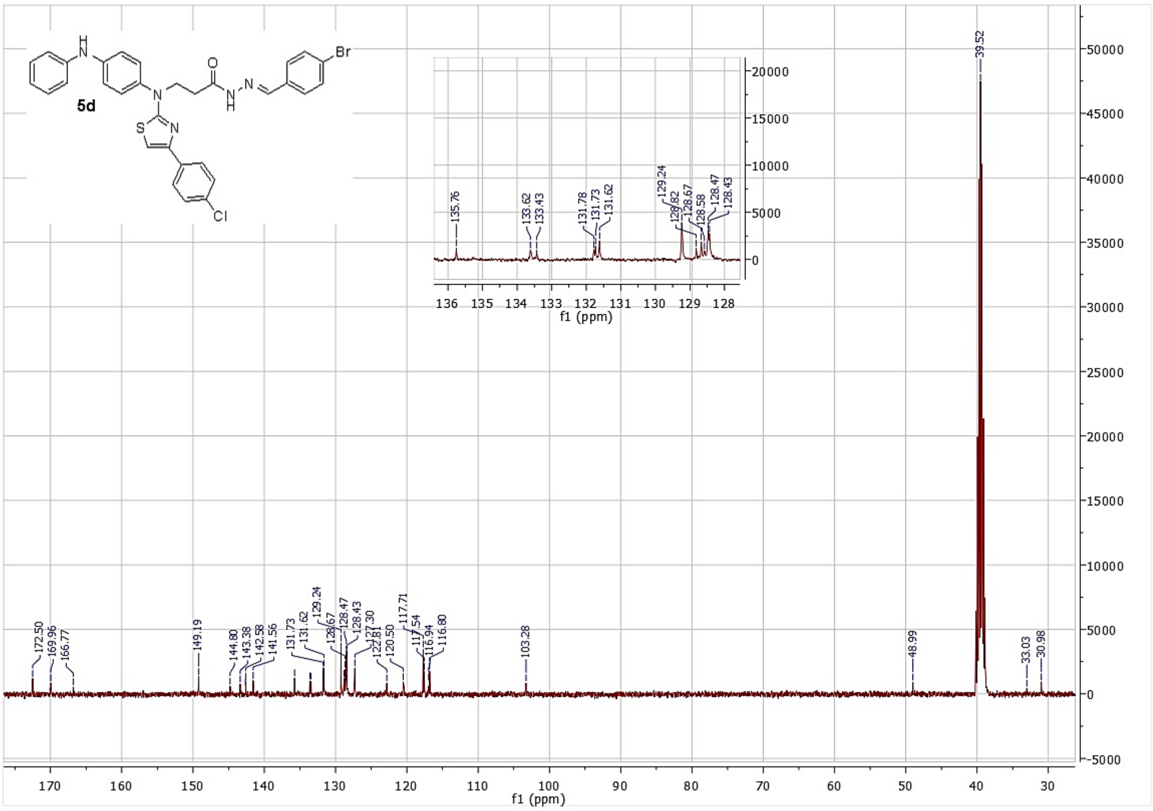

3-((4-(4-chlorophenyl)thiazol-2-yl)-N'-(4-nitrobenzylidene)-(4-(phenylamino)phenyl)amino)propanehydrazide **(5e)**

Figure S21: ^{1}H NMR of compound **5e**

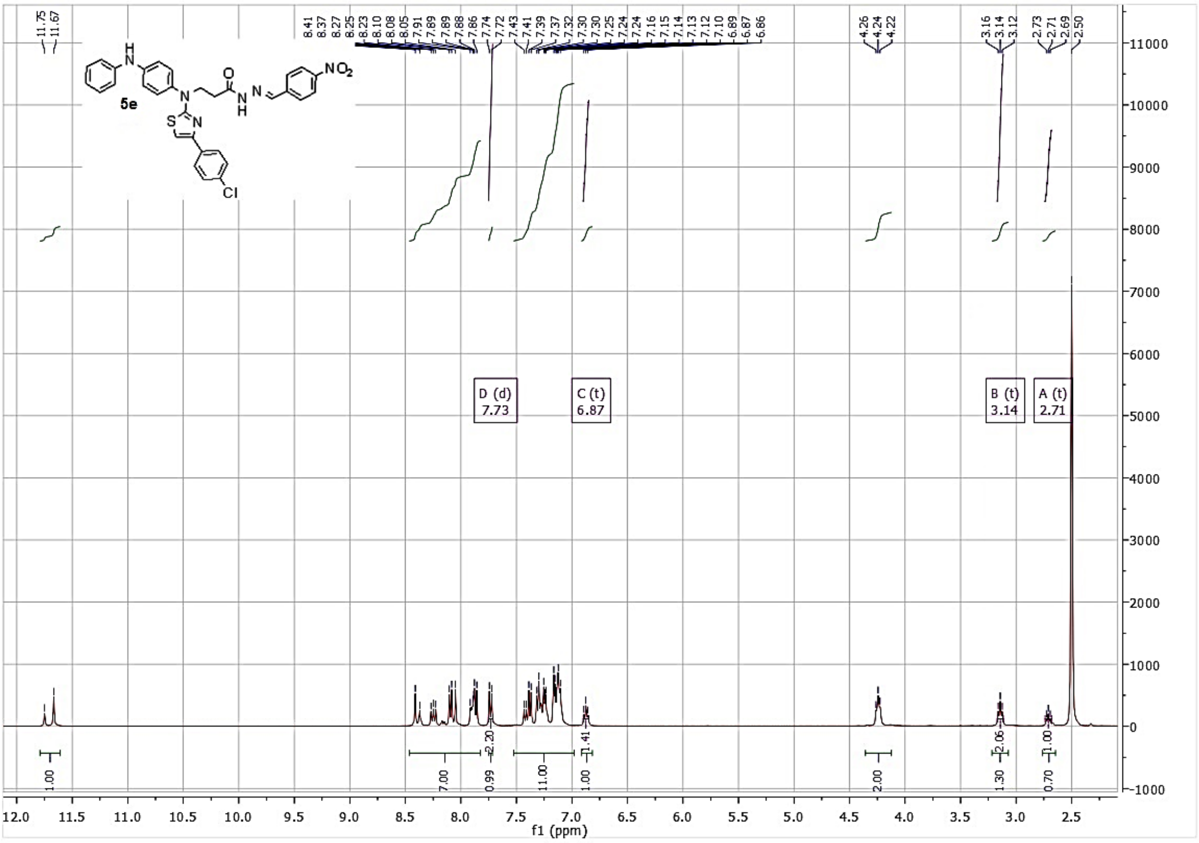

Figure S22: ^{13}C NMR of compound **5e**

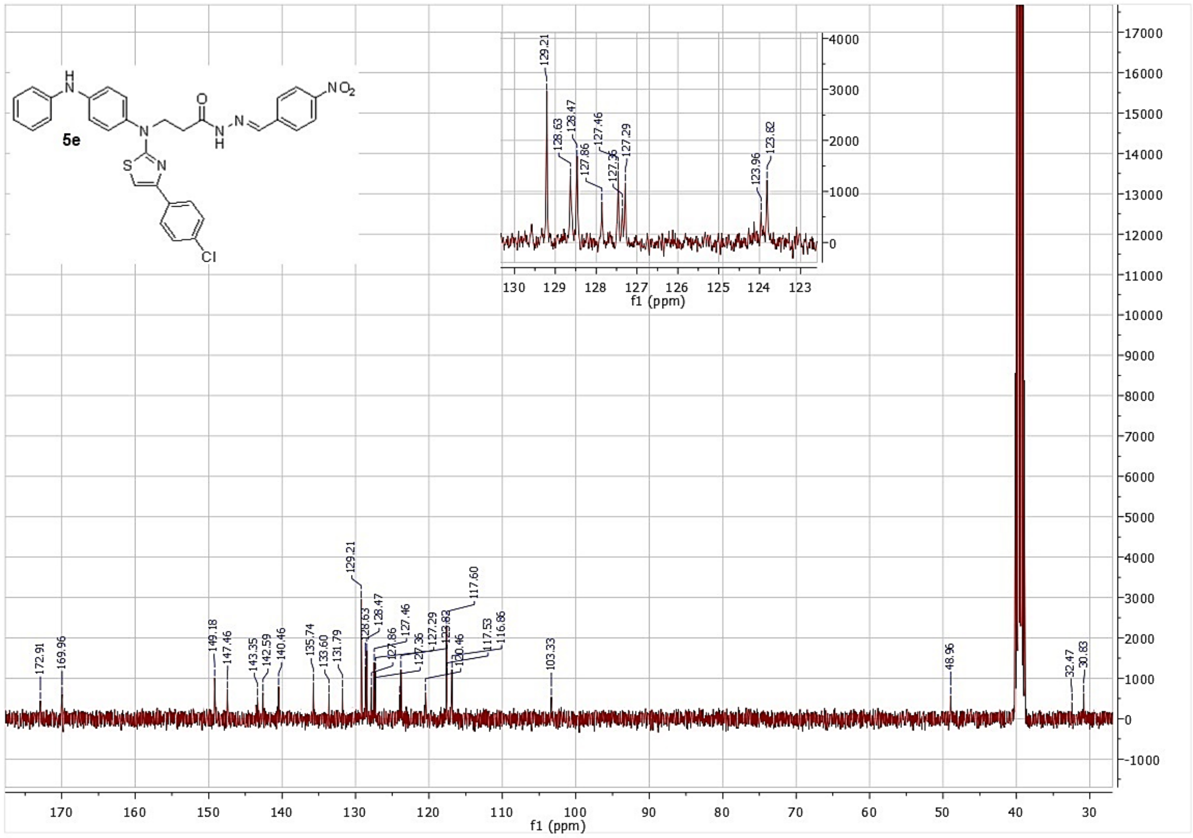

3-((4-(4-chlorophenyl)thiazol-2-yl)-N‘-(4-(dimethylamino)benzylidene)-(4-(phenylamino)phenyl)amino)-propanehydrazide **(5f)**

Figure S23: ^{1}H NMR of compound **5f**

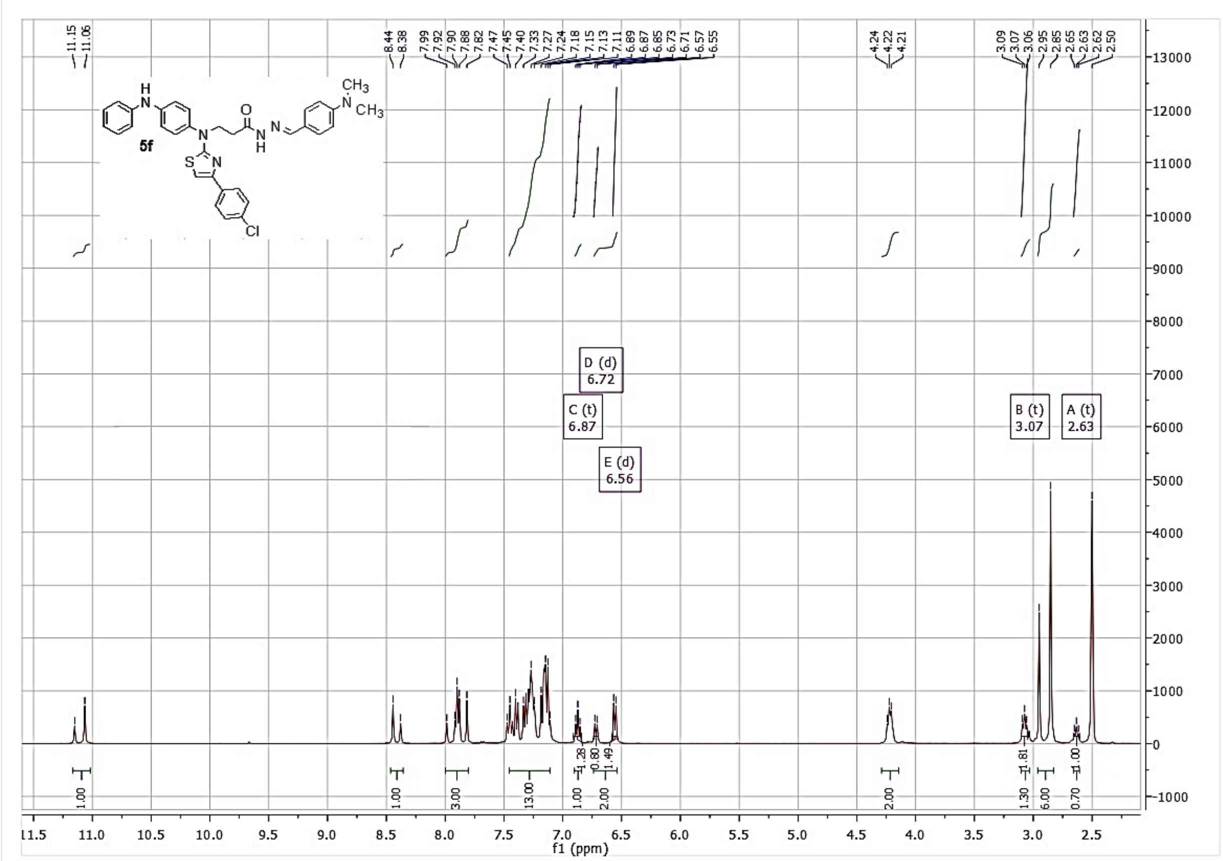

Figure S24: ^{13}C NMR of compound **5f**

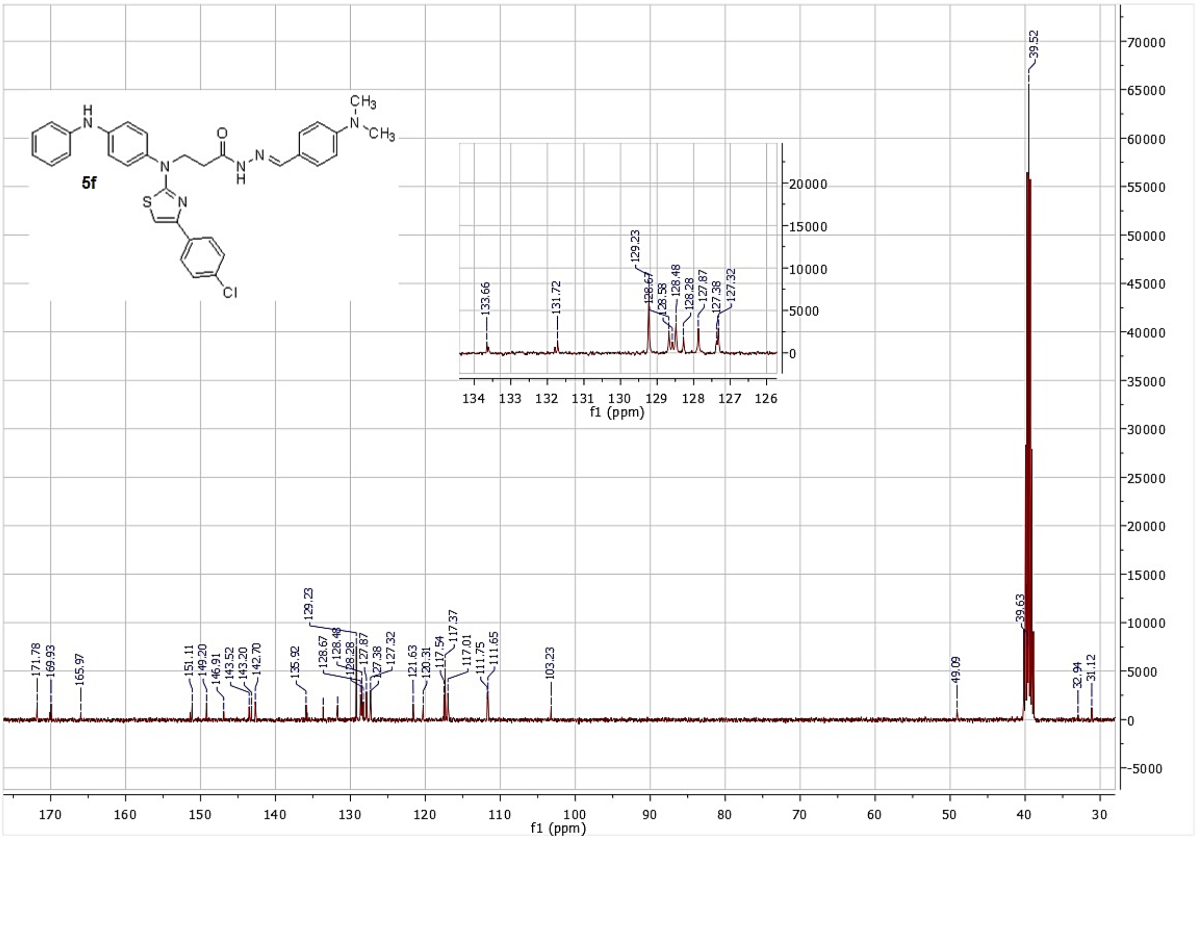

3-((4-(4-chlorophenyl)thiazol-2-yl)-N‘-(4-methylbenzylidene)-(4-(phenylamino)phenyl)amino)propanehydrazide **(5g)**

Figure S25: ^{1}H NMR of compound **5g**

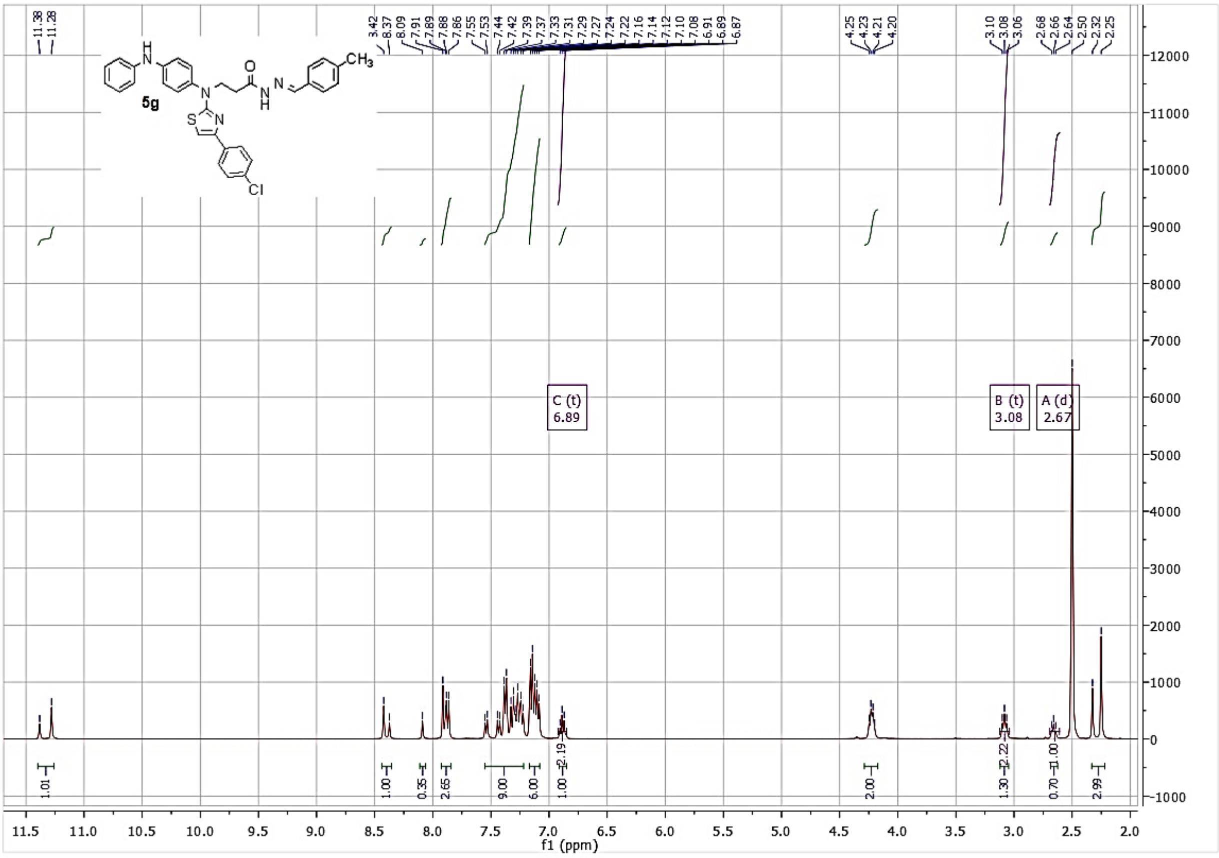

Figure S26: ^{13}C NMR of compound **5g**

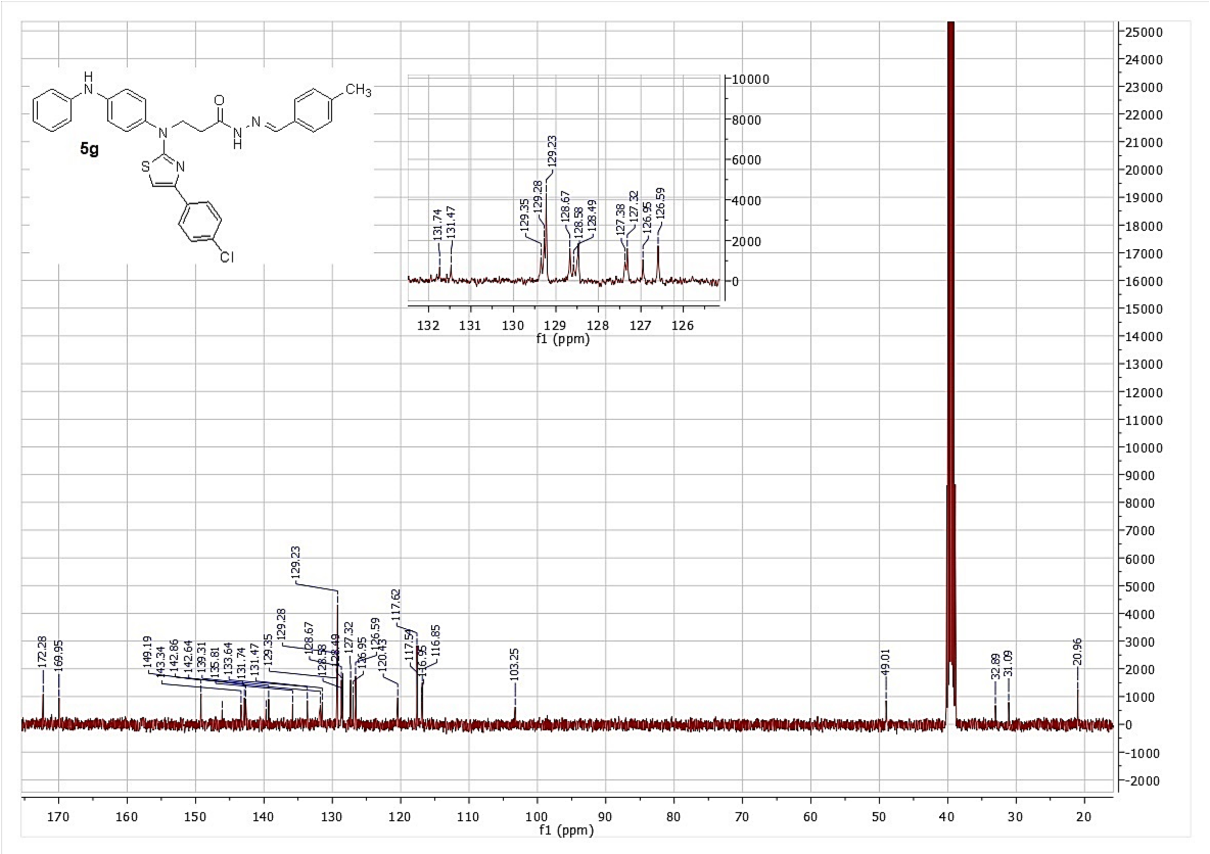

3-((4-(4-chlorophenyl)thiazol-2-yl)(4-(phenylamino)phenyl)amino)-N'-(thiophen-2-ylmethylene)propanehydrazide **(5h)**

Figure S27: ^{1}H NMR of compound **5h**

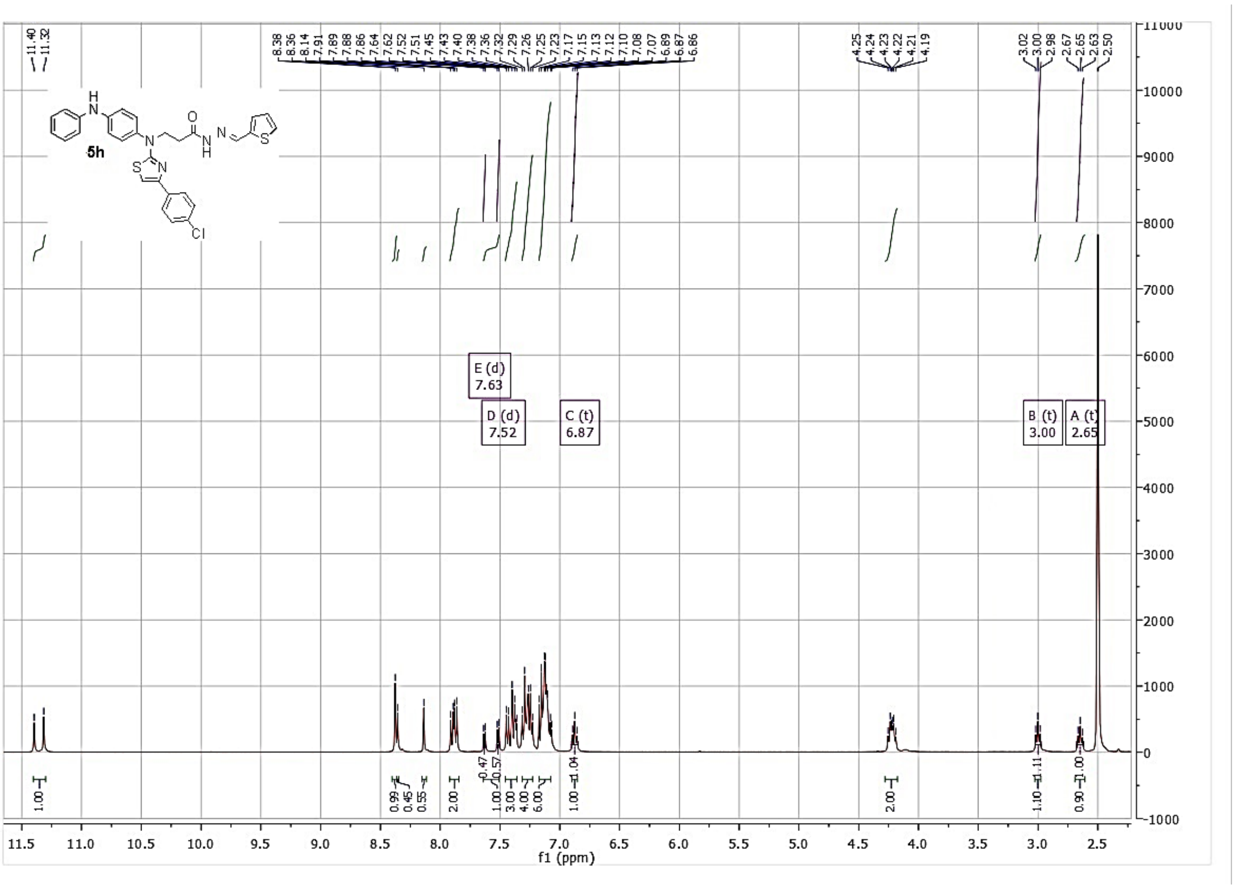

Figure S28: ^{13}C NMR of compound **5h**

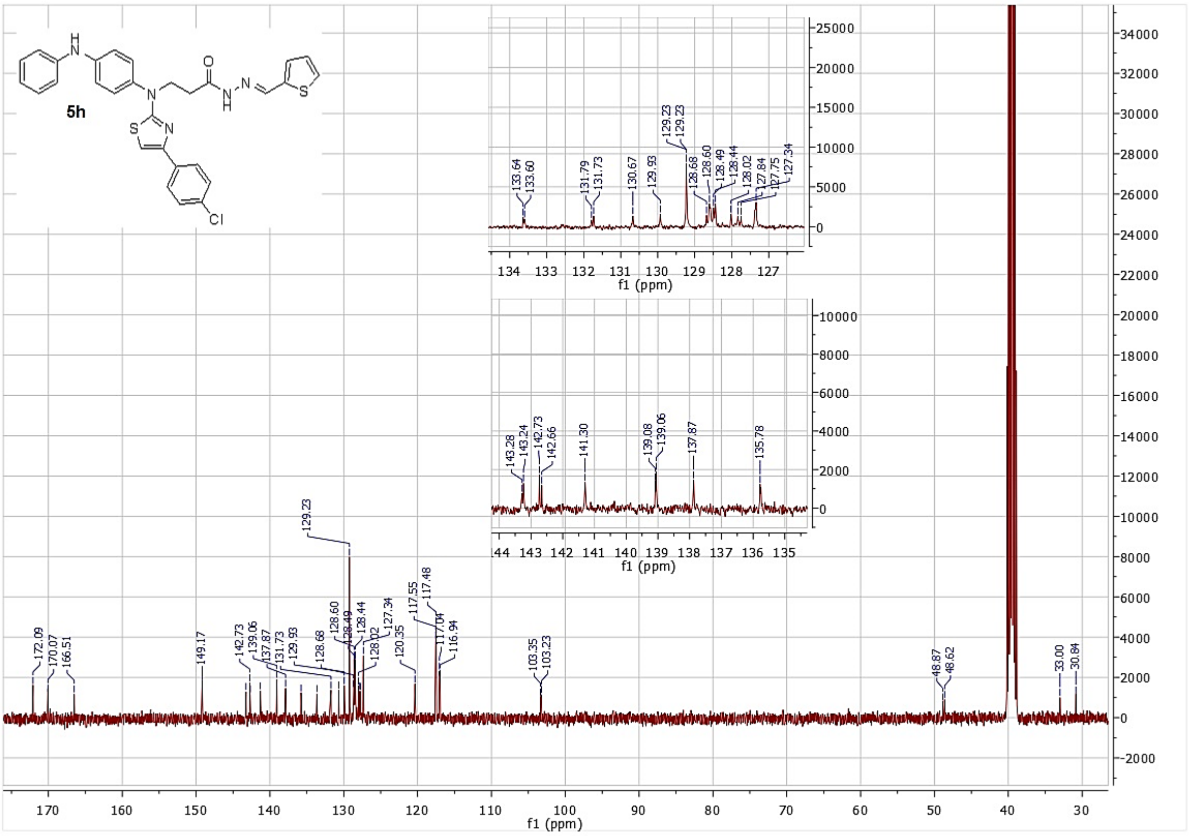

3-((4-(4-chlorophenyl)thiazol-2-yl)(4-(phenylamino)phenyl)amino)-N'-((5-nitrothiophen-2-yl)methylene)propanehydrazide **(5i)**

Figure S29: ^{1}H NMR of compound **5i**

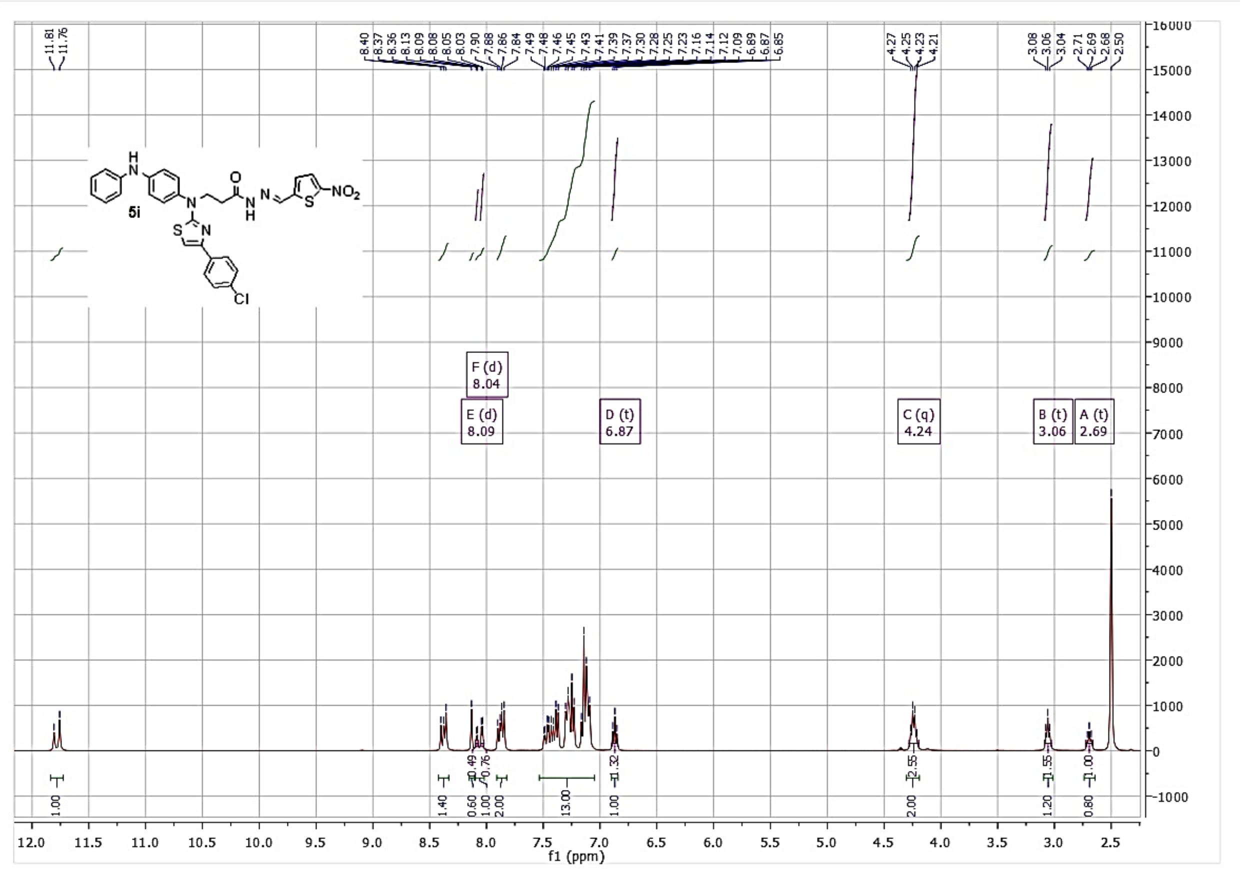

Figure S30: ^{13}C NMR of compound **5i**

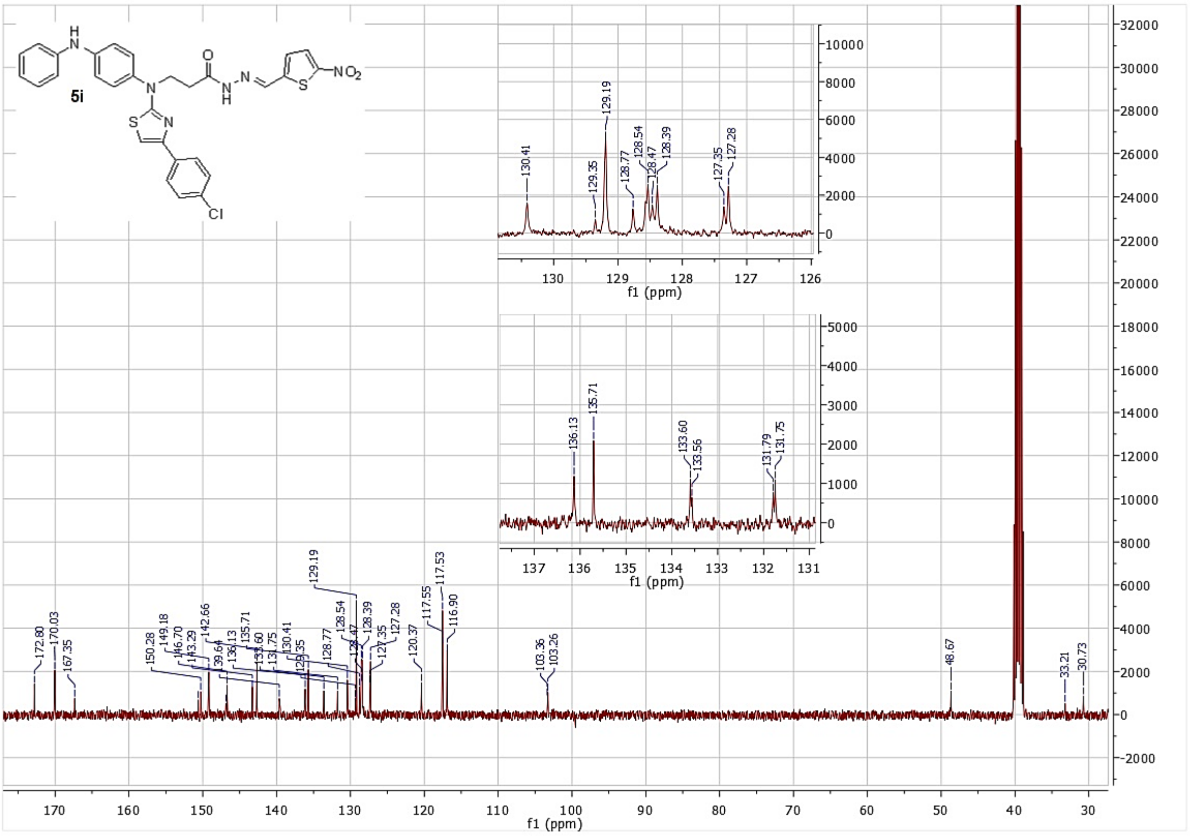

3-((4-(4-chlorophenyl)thiazol-2-yl)(4-(phenylamino)phenyl)amino)-N'-(propan-2-ylidene)propanehydrazide **(5j)**
Figure S31 ^{1}H NMR of compound **5j**

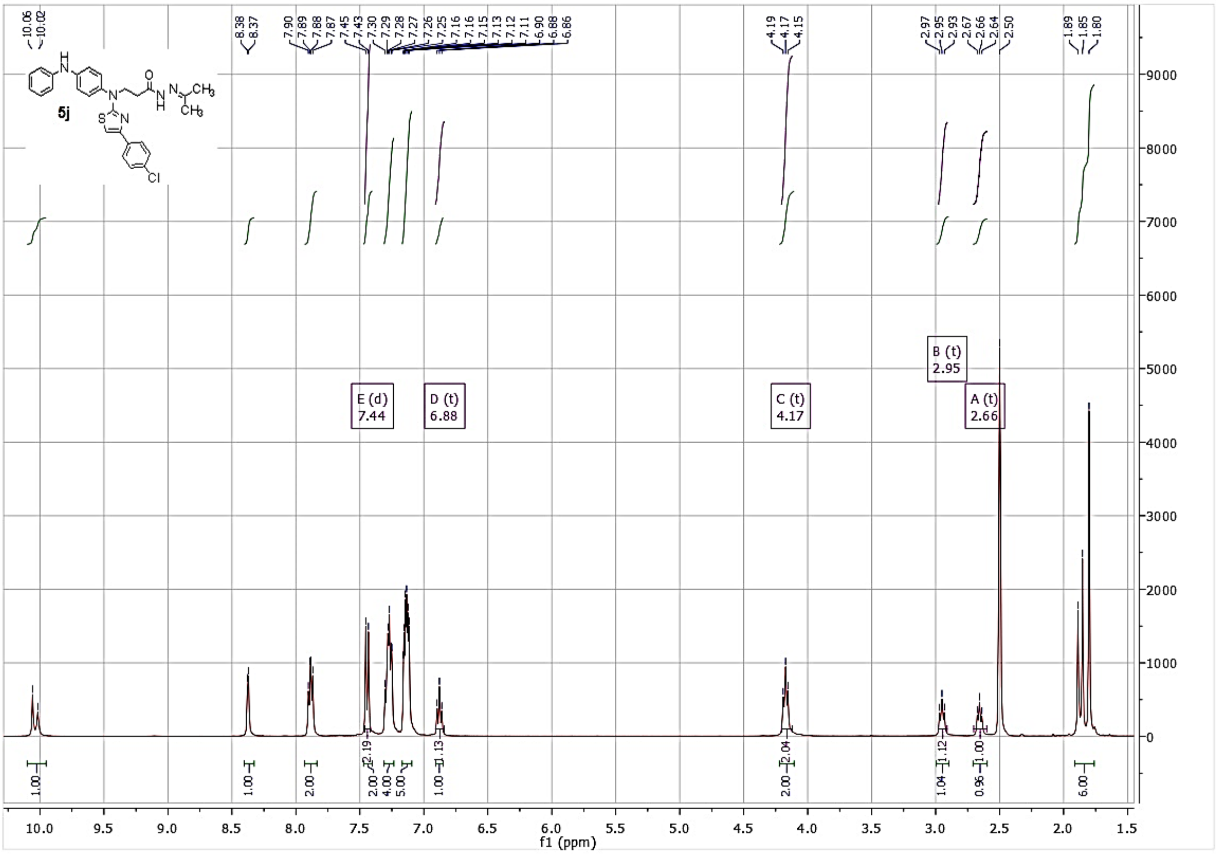

Figure S32: ^{13}C NMR of compound **5j**

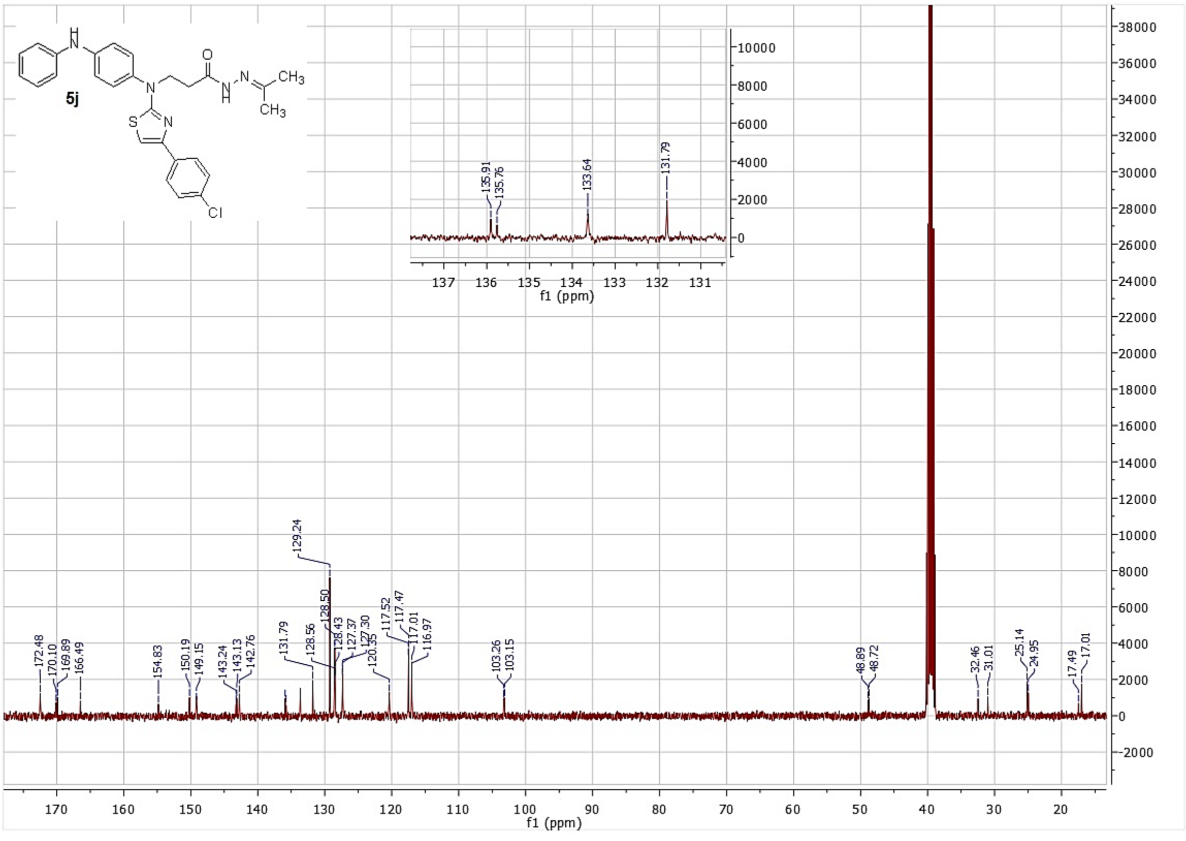

N'-(butan-2-ylidene)-3-((4-(4-chlorophenyl)thiazol-2-yl)(4-(phenylamino)phenyl)amino)propanehydrazide **(5k)**

Figure S33: ^{1}H NMR of compound **5k**

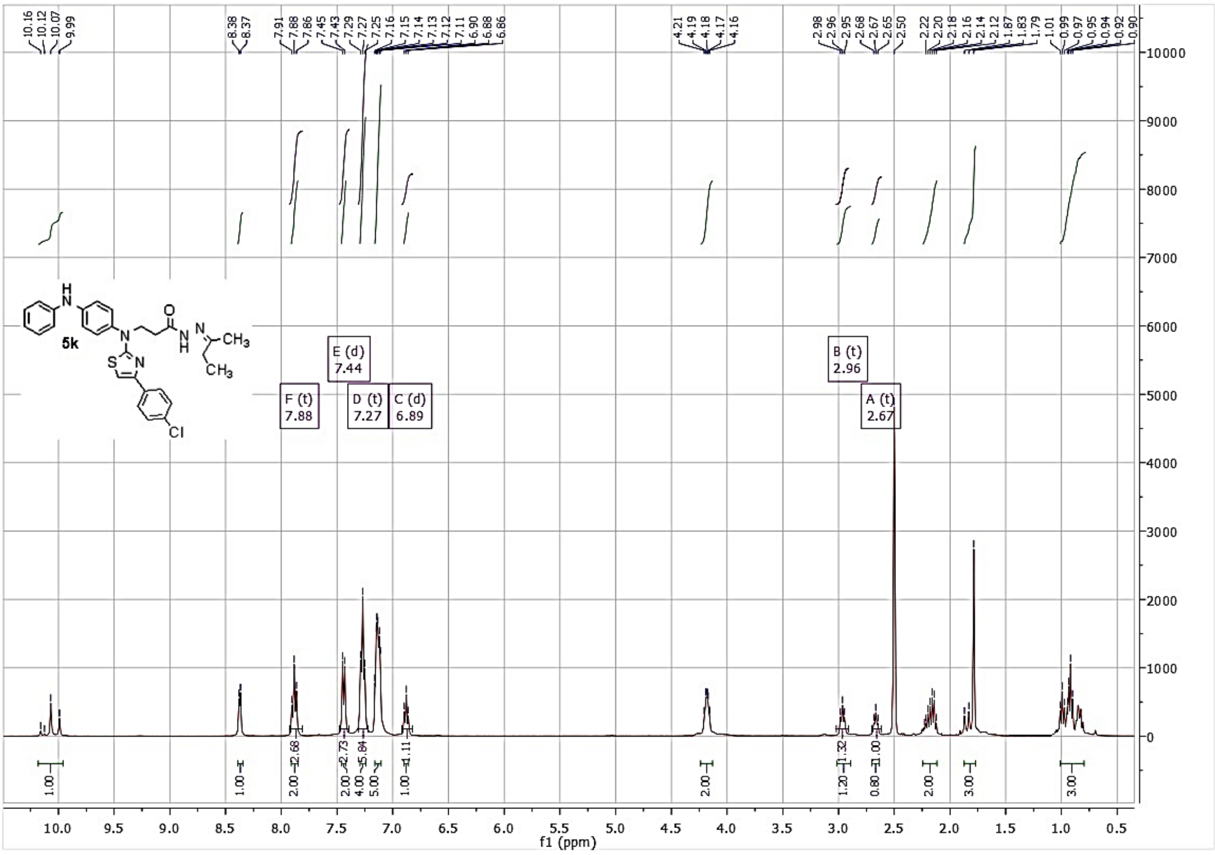

Figure S34: ^{13}C NMR of compound **5k**

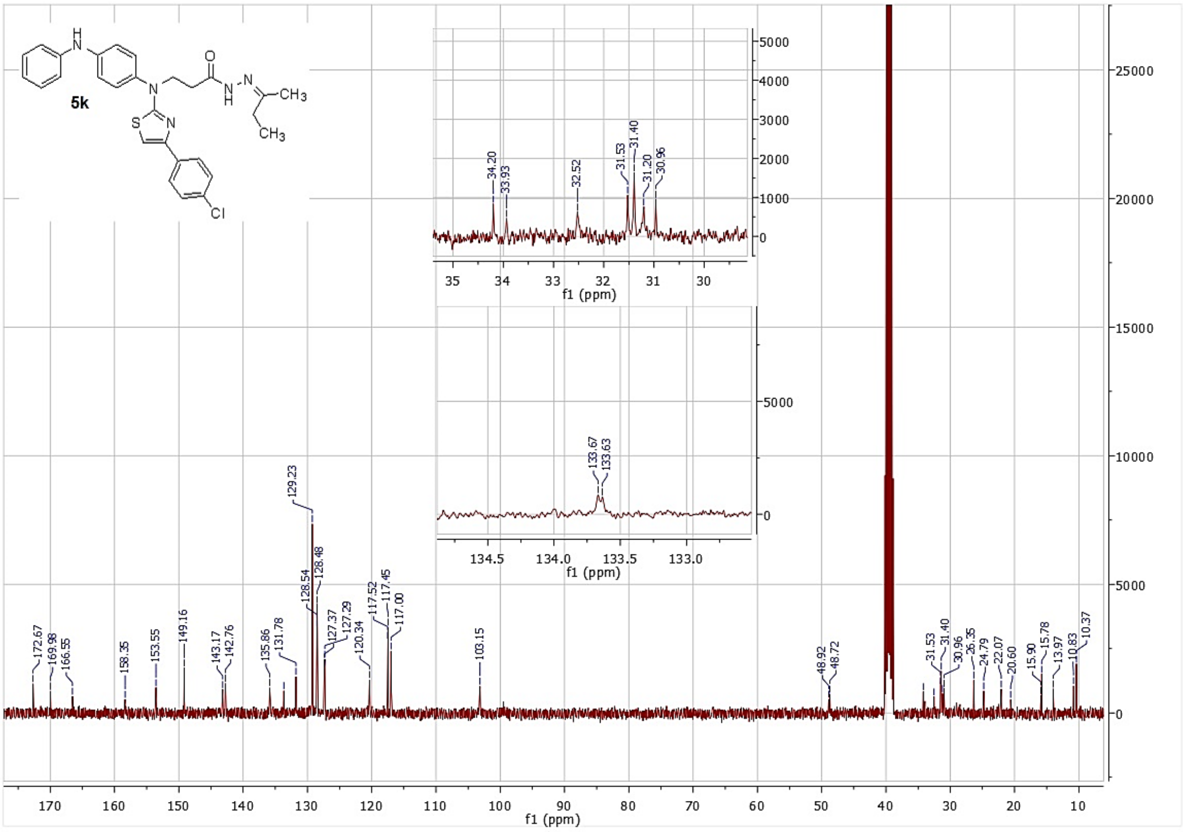

Synthesis of azoles 6-9

3-((4-(4-chlorophenyl)thiazol-2-yl)(4-(phenylamino)phenyl)amino)-1-(3,5-dimethyl-1H-pyrazol-1-yl)propan-1-one **(6)**

Figure S35: ^{1}H NMR of compound **6**

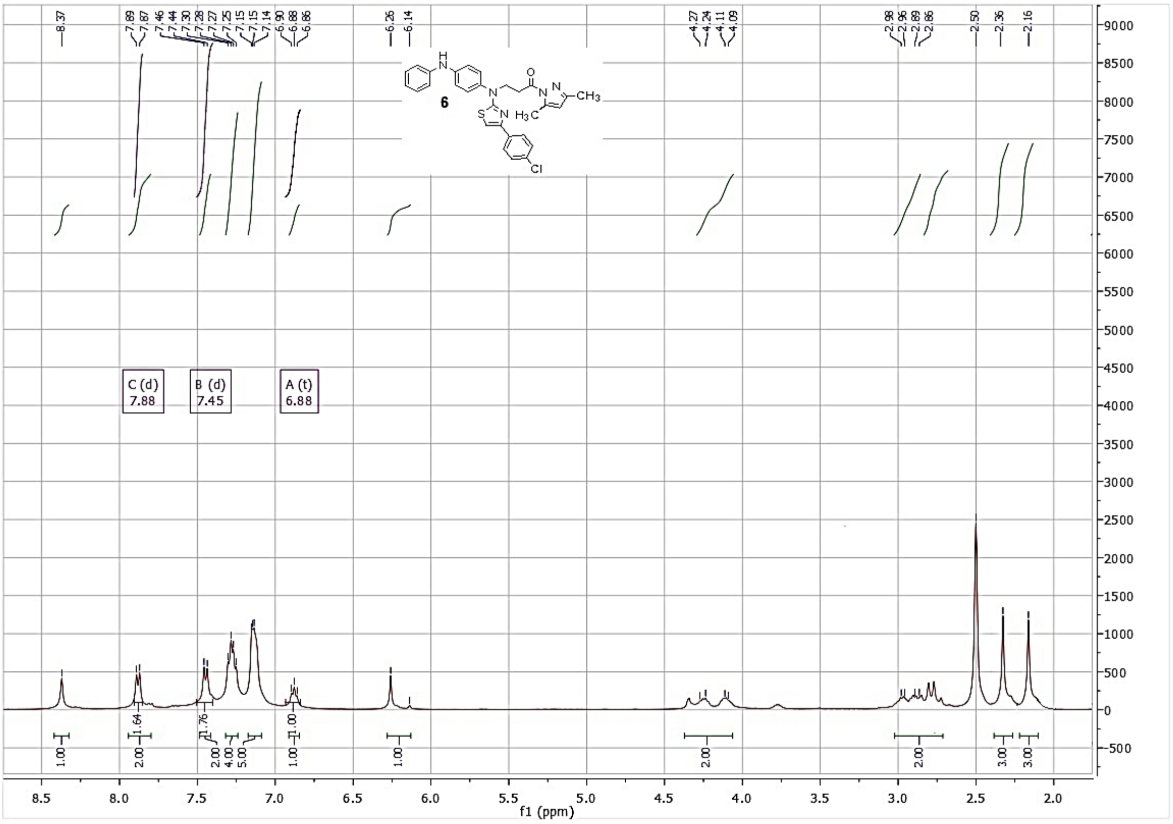

Figure S36: ^{13}C NMR of compound **6**

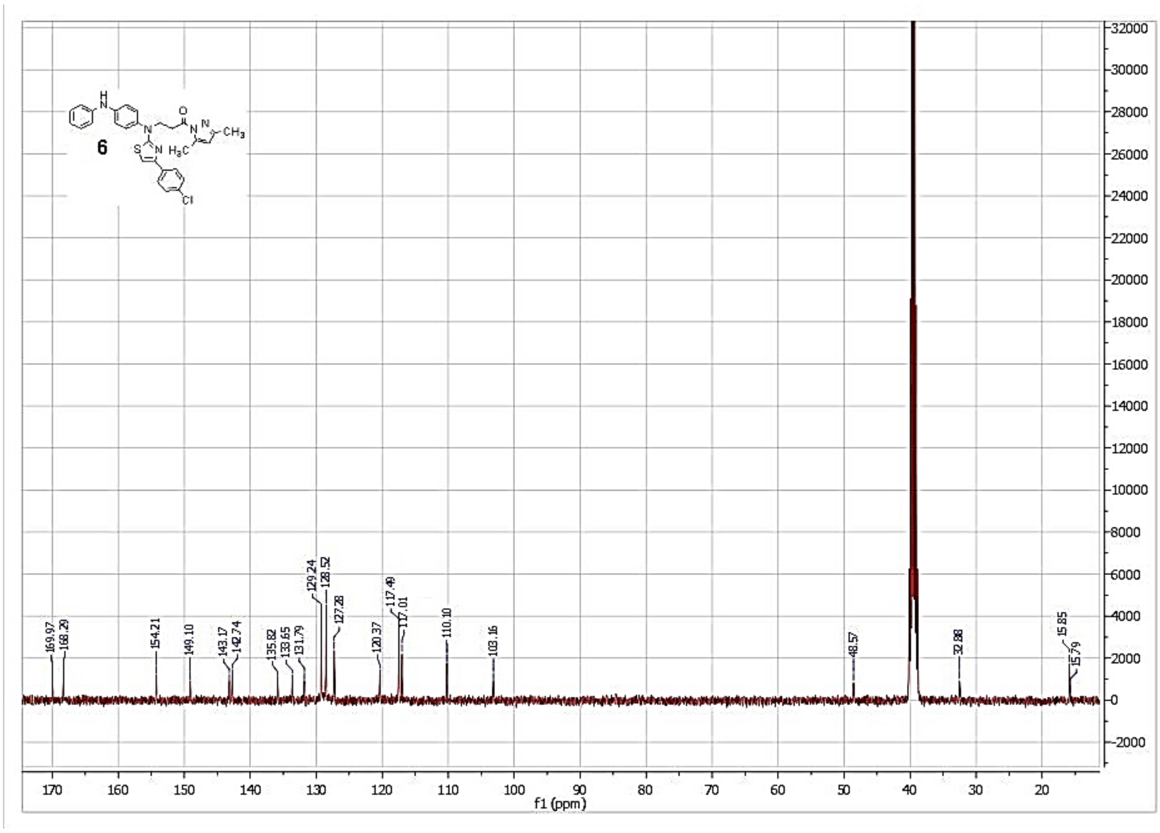

2-(3-((4-(4-chlorophenyl)thiazol-2-yl)(4-(phenylamino)phenyl)amino)-N-(2,5-dimethyl-1H-pyrrol-1-yl)propanamide **(7)**

Figure S37: ^{1}H NMR of compound **7**

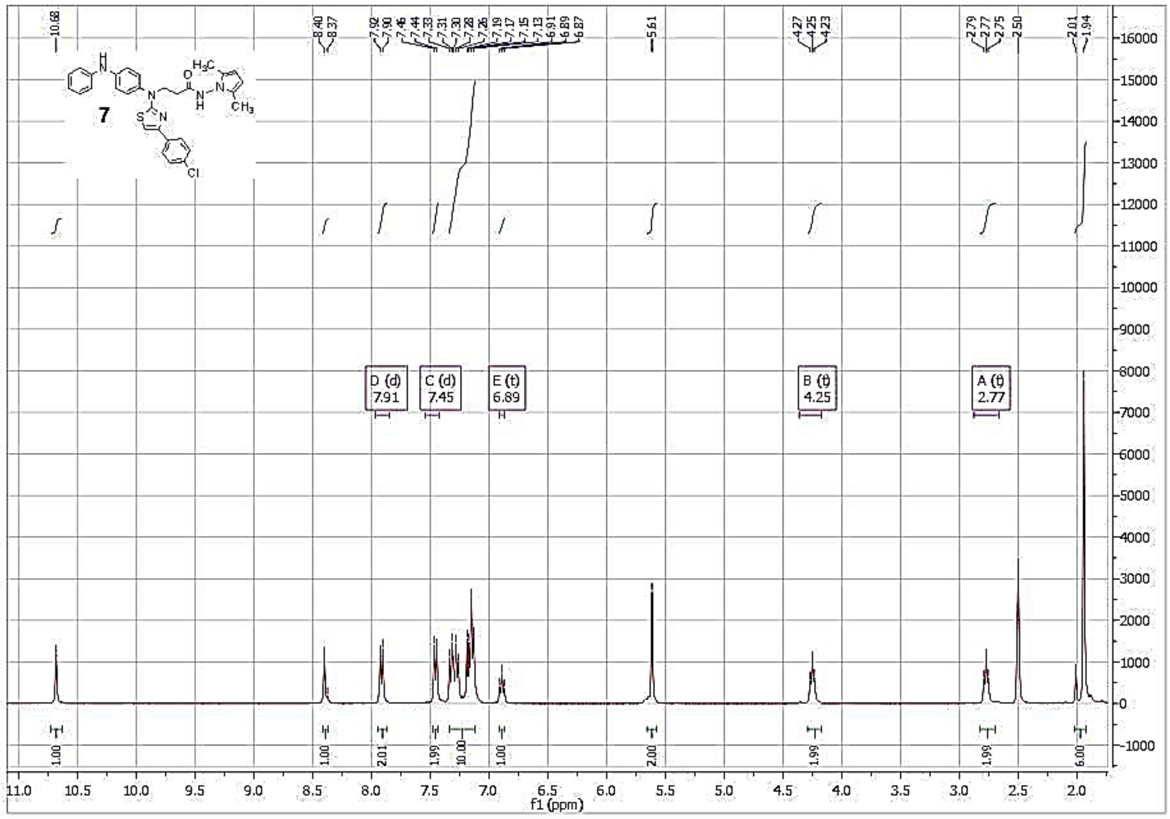

Figure S38: ^{13}C NMR of compound **7**

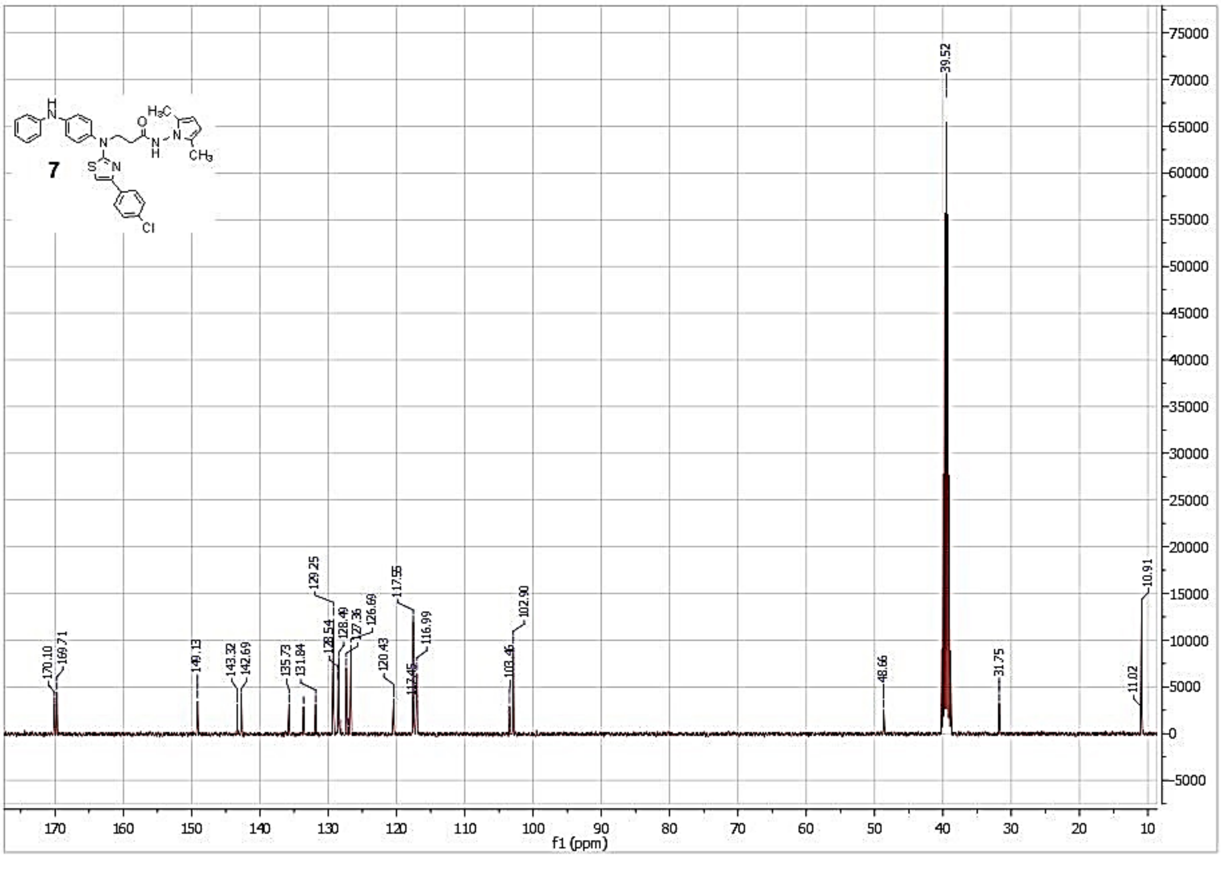

2-(3-((4-(4-chlorophenyl)thiazol-2-yl)(4-(phenylamino)phenyl)amino)propanoyl)-N-phenylhydrazine-1-carbothioamide **(8)**

Figure S39: ^{1}H NMR of compound **8**

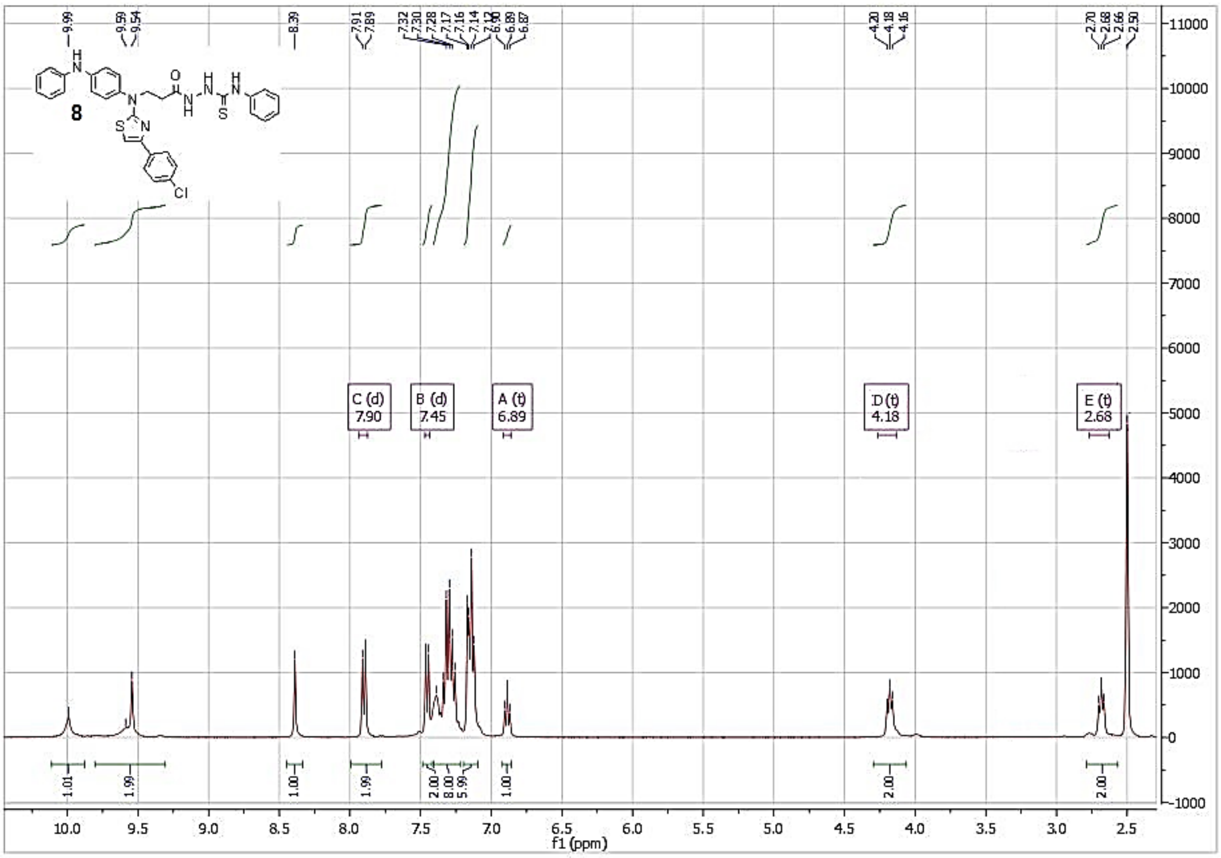

Figure S40: ^{13}C NMR of compound **8**

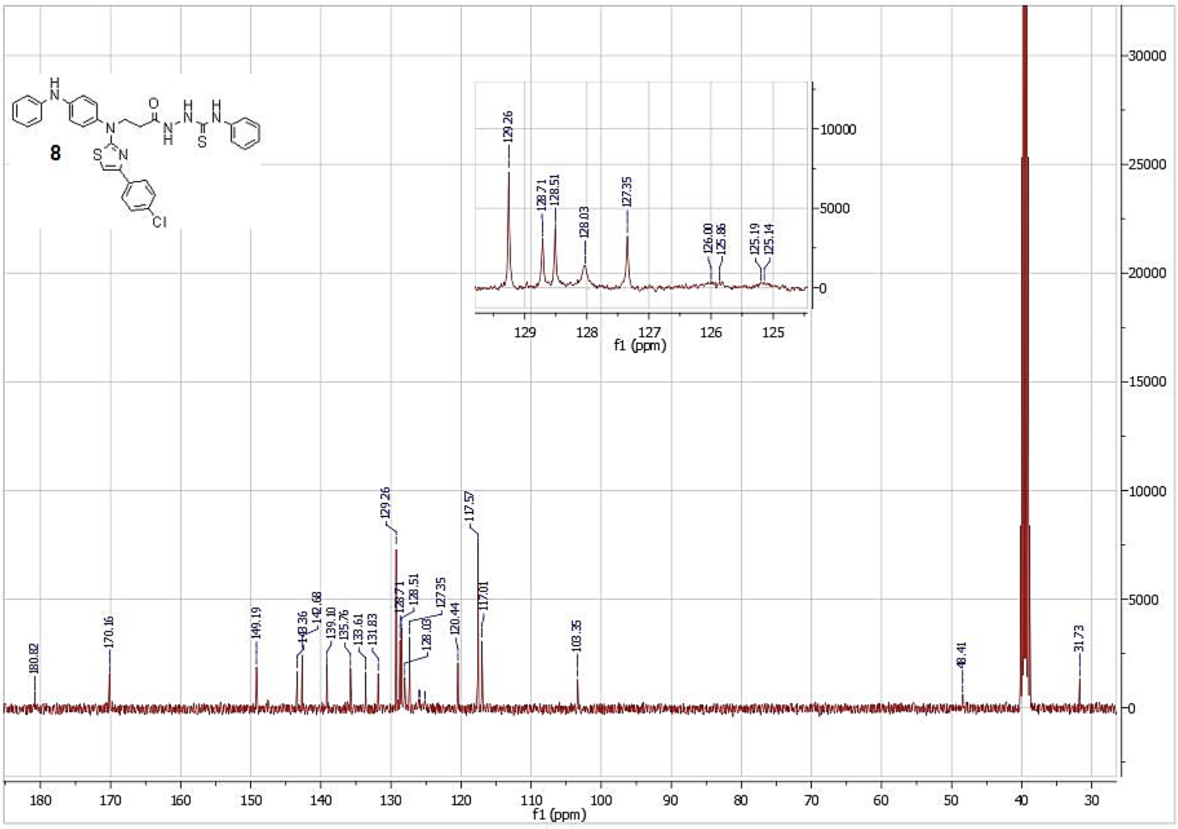

5-(2-((4-(4-chlorophenyl)thiazol-2-yl)(4-(phenylamino)phenyl)amino)ethyl)-4-phenyl-2,4-dihydro-3H-1,2,4-triazole-3-thione **(9)**

Figure S41: ^{1}H NMR of compound **9**

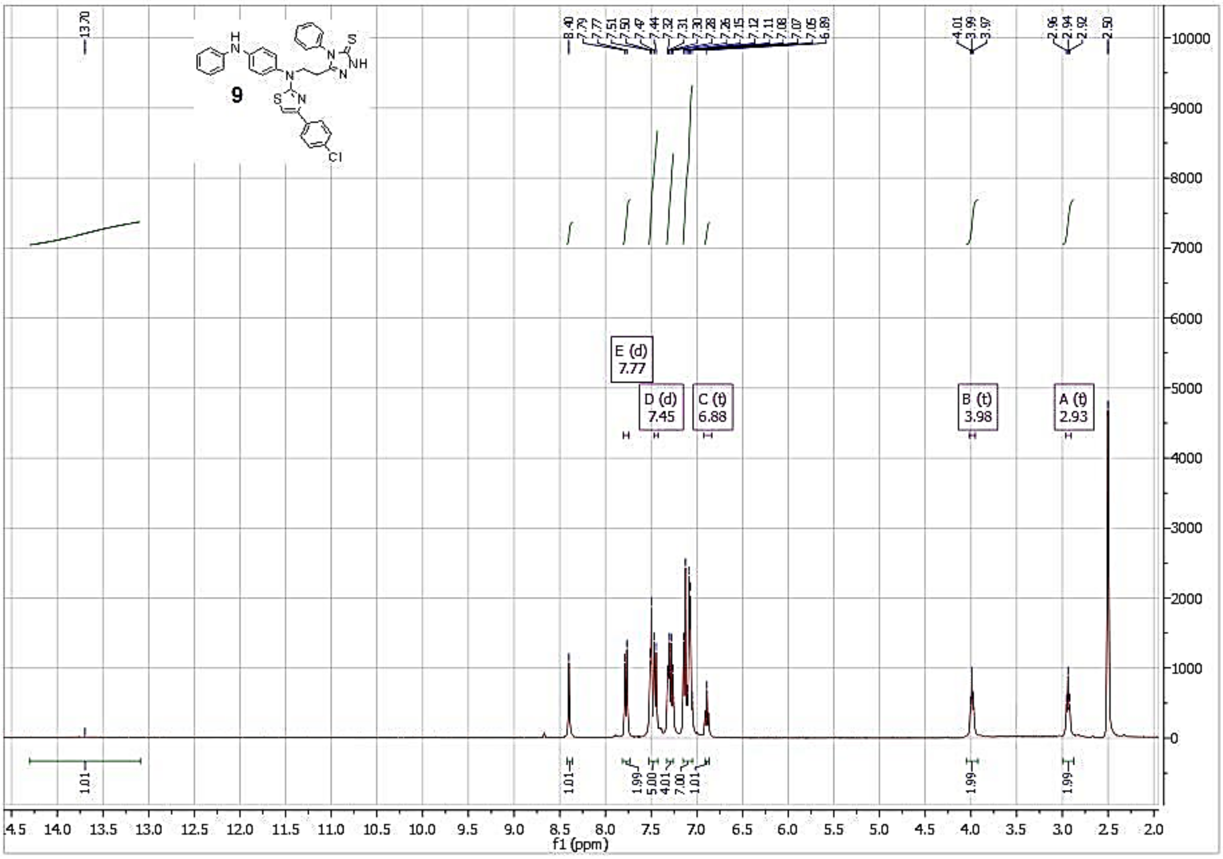

Figure S42: ^{13}C NMR of compound **9**

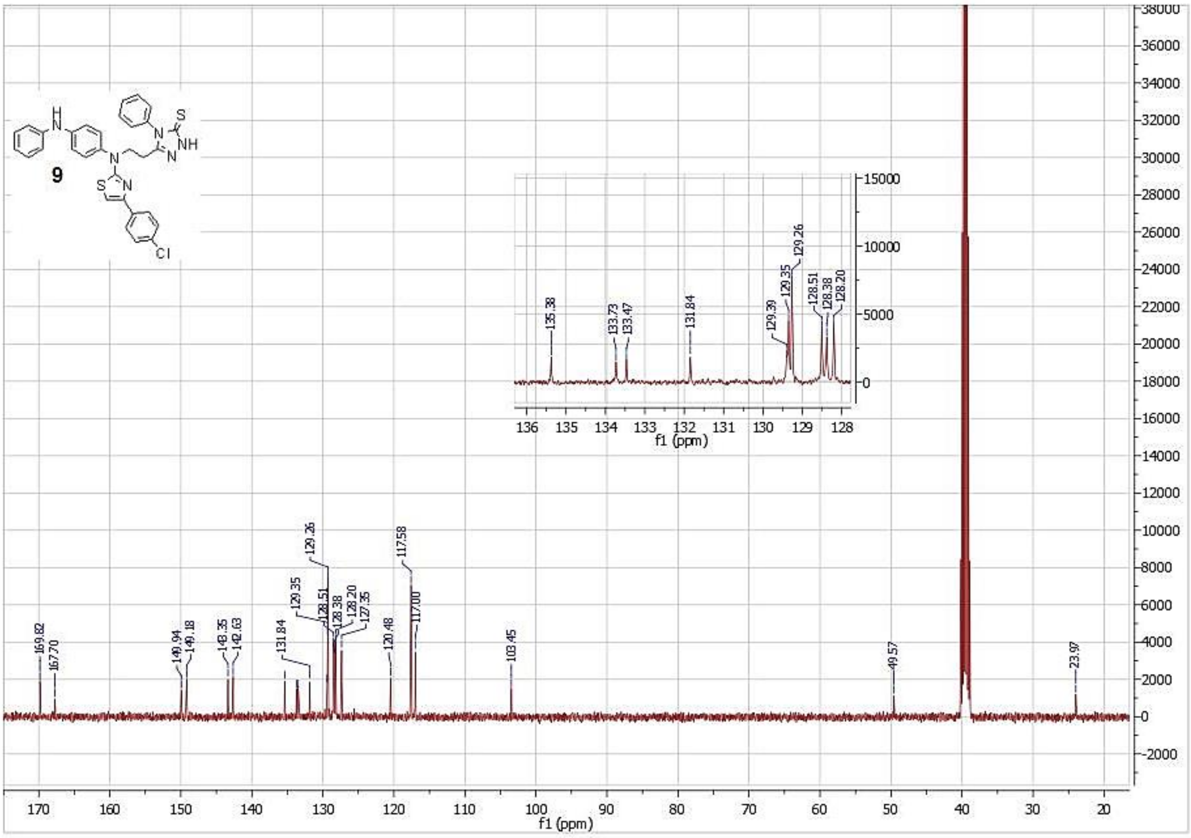

Table S1. The list of fungal strains used for the study

Fungi	Isolate number	Strain feature	Source
Aspergillus fumigatus	731	Azole resistance, L98H, TR34	CDC
Aspergillus fumigatus	732	Azole resistance, F495I, L98H, S297T, TR34	CDC
Aspergillus fumigatus	2122	Pan-susceptible	Laboratory strain
Candida auris	381	Azole resistant	CDC
Candida auris	382	Azole resistant	CDC
Candida auris	383	Azole resistant	CDC
Candida auris	384	Azole resistant	CDC
Candida duobushaemulonii	389	Azole resistant	CDC
Canddia kruseii	397	Azole resistant	CDC
Candida albicans	1214	Azole resistant	Laboratory strain

Abreviations: ATCC-American Type Culture Collection; CDC – Center for Disease Control.
